# I_2_ and the Deep Eutectic Solvent ChCl–Tartaric Acid Promote the Addition–Oxidative Cyclization of 2-Aminopyridines and Chalcones to Obtain Imidazo[1,2-*a*]pyridines

**DOI:** 10.3390/molecules31091416

**Published:** 2026-04-24

**Authors:** Juan Lopez de Leon, Nayely Melissa Cruces Velazco, Arlette Richaud, Francisco Méndez, Diego A. Alonso, Claudia Araceli Contreras-Celedón

**Affiliations:** 1 Departamento de Síntesis Orgánica, Instituto de Investigaciones Químico Biológicas, Universidad Michoacana de San Nicolás de Hidalgo, Morelia 58030, Mexico; juan.lopez@umich.mx (J.L.d.L.); 2431702g@umich.mx (N.M.C.V.); 2 Conditions Extrêmes et Matériaux: Haute Température et Irradiation, CEMHTI—UPR3079 CNRS, 1 Avenue de la Recherche Scientifique, 45100 Orléans, France; avrt@xanum.uam.mx; 3 Departamento de Química, División de Ciencias Básicas e Ingeniería, Universidad Autónoma Metropolitana-Iztapalapa, Ciudad de Mexico 09340, Mexico; 4 Department of Organic Chemistry and Organic Synthesis Institute (ISO), Alicante University, Apdo. 99, 03080 Alicante, Spain

**Keywords:** imidazo[1,2-*a*]pyridine, chalcone, 2-aminopyridine, deep eutectic solvent, iodine

## Abstract

The synthesis of nitrogen-containing heterocycles remains a subject of significant interest due to their applications in medicinal chemistry and materials science. This paper describes the preparation of imidazo[1,2-*a*]pyridine using a catalytic system consisting of the deep eutectic solvent (DES) choline chloride (ChCl)–tartaric acid (1:2) and I_2_ by reaction between 2-aminopyridines and chalcones (1,3-diphenylprop-2-en-1-ones). The proposed mechanism suggests the activation of the chalcone carbonyl by the DES, enhancing the polarization of the conjugated system which suffers electrophilic addition by I_2_ to the C=C bond. The resulting intermediate undergoes a nucleophilic attack by 2-aminopyridine followed by cyclization and iodine-promoted oxidation and aromatization to yield the corresponding imidazo[1,2-*a*]pyridine products. The role of the DES is crucial, as it facilitates carbonyl activation through hydrogen bond interactions, stabilizes reactive intermediates, and promotes protonation–deprotonation steps, thereby eliminating the need for metal catalysts or toxic organic solvents. Theoretical calculations at the PM6 level of theory suggest that the DES acts as a catalyst in this reaction, due to the nature of its components enabling the development of more sustainable synthetic strategies.

## 1. Introduction

**Figure 1:**
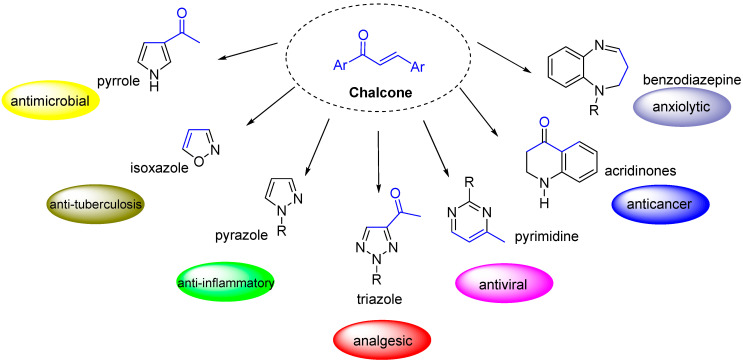
N-heterocyclic compounds obtained from chalcone and their probable biological activity. Numbers above the reaction arrows indicate literature references.

Functionalization reactions of α,β-unsaturated systems play a vital role in synthetic processes, as they facilitate the formation of C-C and C-N bonds [1,2]. This relevance is particularly notable in the field of research and development of therapeutic agents. One such α, β-unsaturated system that has been identified as a key structure in drug discovery is that of chalcones [3]. Multiple synthetic procedures have been developed for their preparation [4], as well as their functionalization through Aza-Michael addition, which has enabled the synthesis of various libraries of biologically active N-heterocyclic compounds [5]. These include pyrroles [6,7], isoxazoles [8,9], pyrazoles [10,11], triazoles [12], pyrimidines [13,14], and fused five-, six-, and seven-membered systems, such as imidazopyridines [15], acridinones [16,17], and benzodiazepines [18,19]. These heterocyclic compounds are of significant value due to their broad pharmacological activities as antimicrobials [20], antituberculosis [21], anti-inflammatory [22], analgesic [23], and antiviral agents [24], anticancer [25] and anxiolytic [26] among others (Figure 1).

**Figure 2:**
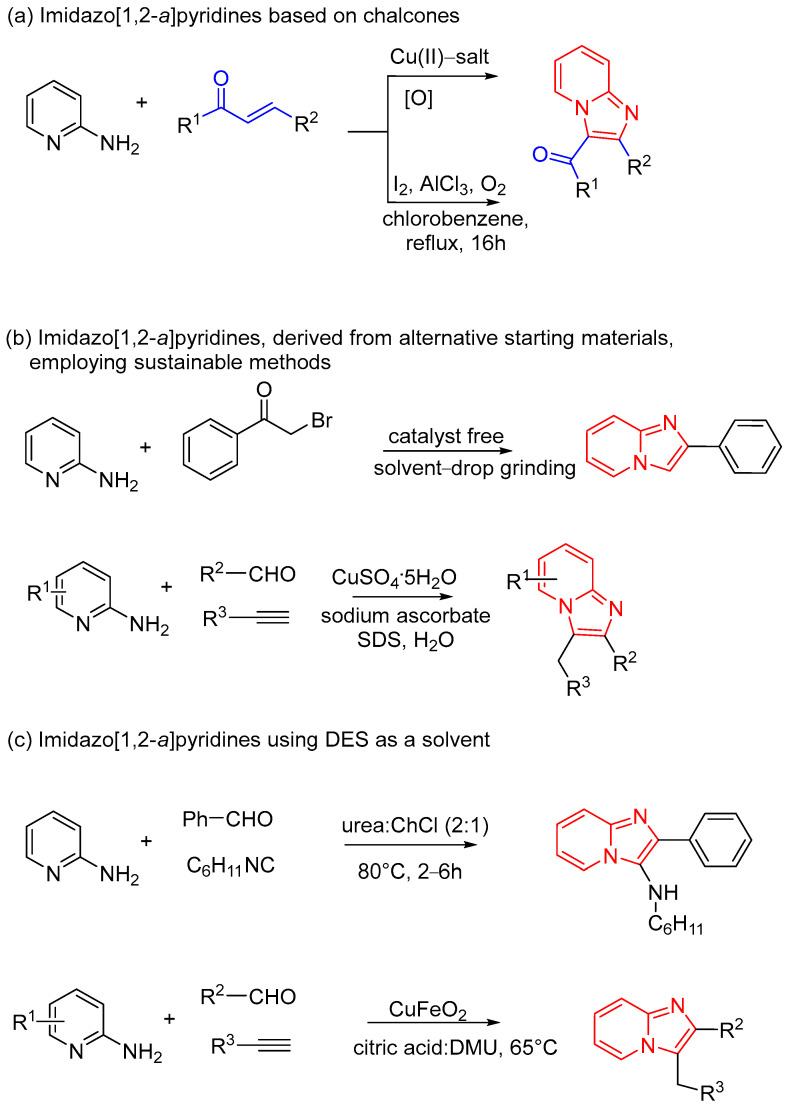
Synthetic methods of producing imidazo[1,2-*a*]pyridines. (**a**) Use of chalcones as starting materials; (**b**) Use of alternative starting materials; (**c**) Utilizing DES as a reaction medium.

Furthermore, imidazo[1,2-*a*]pyridines constitute a class of nitrogen-containing heterocyclic compounds that have garnered considerable attention due to their broad spectrum of biological activities [27,28]. Some of those compounds have demonstrated noteworthy antifungal [29], antibacterial [30], antiviral [31], and anti-inflammatory [32] properties, highlighting the potential of imidazo[1,2-*a*]pyridine as a valuable scaffold for pharmaceutical innovation. The synthetic procedures used to obtain the mentioned active imidazo[1,2-*a*]pyridine derivatives include photochemical reactions [33], multicomponent processes [34], biocatalytic processes [35], oxidative cyclization [36], C-H bond activation [37], cycloadditions [38], electrochemical reactions [39], and ring openings [40]. The application of chalcones as key precursors in the synthesis of imidazo[1,2-*a*]pyridine has been explored through various methodologies, including protocols employing metal catalysts [41] and I_2_/Lewis acids [42] (Figure 2a). Further improvements for the synthesis of this type of compound include the use of sustainable solvents, such as water [43,44] (Figure 2b) or DES [45,46,47] (Figure 2c). The use of DESs as alternative reaction media is particularly interesting due to their tunable physicochemical properties, low vapor pressure, facile preparation, and potential dual role as solvent and catalyst [48]. Their composition can be readily adjusted through the selection of suitable hydrogen-bond donors and acceptors, allowing modulation of key parameters such as polarity, acidity, viscosity, and hydrogen-bonding capacity according to the requirements of a given transformation. In synthetic applications, DESs can enhance substrate activation, stabilize charged or polar intermediates, and promote proton transfer processes, which often leads to improved selectivity and reduced dependence on additional catalytic systems. Furthermore, their generally simple preparation, often from inexpensive and readily available components, together with the possibility of recycling and reuse, makes DESs particularly attractive within the context of sustainable and metal-free synthetic methodologies [49,50].

**Scheme 1:**
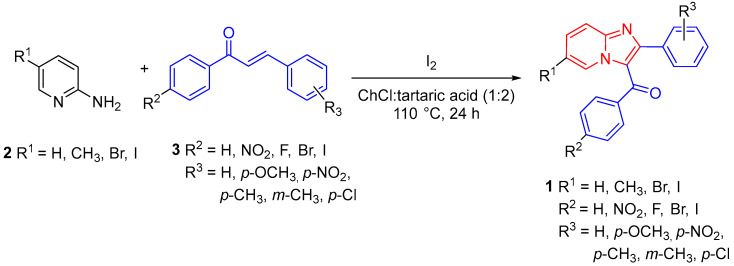
Generalized reaction for imidazo[1,2-*a*]pyridines’ synthesis.

Conversely, metal-free synthetic proposals that meet sustainability criteria pose a significant challenge for refining synthetic methodologies targeting imidazo[1,2-*a*]pyridine [47]. Iodine, classified as a weak Lewis acid, acts as a non-metallic catalyst, demonstrating remarkable efficacy in the formation of C-C and C-N bonds in heterocyclic synthesis [51,52]. Furthermore, it can be obtained from renewable sources, such as seaweed. This paper presents the experimental results of I_2_ catalysis in the presence of DES in the synthesis of imidazo[1,2-*a*]pyridine. Chalcones and 2-aminopyridines were used as raw materials for this study (Scheme 1). Theoretical calculations at the PM6 level of theory are also presented.

## 2. Results and Discussion

This study began with the optimization of the conditions for the reaction between 2-aminopyridine (**2a**) and 1,3-diphenylpropenone (**3a**) in the presence of an equimolar amount of molecular iodine as an oxidizing agent and various DESs as reaction media. The reaction was carried out at 110 °C for 24 h (Table 1, entries 1–9). Under these conditions, limited yields were observed for the expected product when using the DESs ChCl–tartaric acid (1:2), ChCl–*p*-TsOH (1:1), ChCl–glycerol (1:2), and ChCl–DMU (1:2), respectively (Table 1, entries 3–5, and 7). In contrast, when DESs such as ChCl–oxalic acid (1:2), ChCl–malonic acid, and ChCl–urea (1:2) were used, only traces of **1aa** were obtained (Table 1, entries 1, 2, and 6). On the other hand, with the DESs glycerol–K_2_CO_3_ (2:1) and ChCl–ZnCl_2_ (1:2), no reaction product was observed (entries 8 and 9).

Although the results of these assays were not as expected, due to the formation of unidentified undesirable by-products, they did illustrate the effect of the nature of the DES on the reaction under study. Acidic DES [53], as in the case of the ChCl–oxalic acid (1:2) and ChCl–malonic acid (1:2) assays (Table 1, entries 1 and 2), did not favor the formation of product **1aa**, and only traces were detected. On the other hand, moderate yields of **1aa** of 23% and 19% were obtained with the DESs ChCl–tartaric acid (1:2) and ChCl–*p*-TsOH (1:1) (Table 1, entries 3 and 4). Given that the results could not be clearly correlated with the acidity of the different DESs used, it seems clear that there is a synergistic effect between the acidity of the DES and the oxidative role of iodine to promote activation of the carbonyl group in chalcone **3a**, thereby facilitating the subsequent oxidative cyclization. When the reaction was carried out in a low-acidic or neutral DES, such as ChCl–glycerol (1:2) and ChCl–DMU (1:2), product **1aa** was still obtained, although in reduced yields of 15% and 11%, respectively (Table 1, entries 5 and 7). These results suggest that, while the polarity of the eutectic medium and the presence of iodine facilitate oxidative cyclization, insufficient acidity limits amine protonation and reduces reaction efficiency. In contrast, in basic DES, such as glycerol–K_2_CO_3_ (2:1), the formation of **1aa** was completely suppressed. This suppression can be attributed to the lack of protonation of the amine intermediate, a critical step for successful cyclization. Similarly, no product formation was observed in the case of ChCl–ZnCl_2_ (1:2) (Table 1, entry 9), which may be explained by the strong tendency of Zn^2+^ ions to coordinate with the carbonyl moiety of chalcone which decreases the nucleophilicity of the double bond towards a plausible initial electrophilic addition of iodine [54]. These results highlight the pivotal role of the acid–base balance of the DES in controlling the efficiency of the transformation. While moderate acidity promotes the process by enabling both carbonyl activation and amine protonation, conditions of excessive acidity, strong basicity, or competitive coordination (e.g., in metal-containing DESs) are detrimental, resulting in partial or complete inhibition of product formation. In addition, as shown in Table 1 (entries 1–9), the nature of the DES has a significant impact on the reactivity of these species, enabling the modulation of the different reaction steps to achieve the desired product. Specifically, acidic DESs (Table 1, entries 1–5) showed superior yields compared to DESs formed by choline chloride and basic components (Table 1, entries 6–8), suggesting a synergy between the acidic nature of the hydrogen donor component and the stabilizing capacity of the eutectic medium at critical stages of the reaction. This behavior may be attributed to the ability of acidic organic DES to facilitate the activation of the α,β-unsaturated system and stabilize the reactive intermediate species during the cyclization process. Our results contrast with those observed in DES with different acidity and hydrogen-bonding characteristics (e.g., ChCl–urea or ChCl–glycerol), where lower conversion and selectivity towards the heterocyclic product were detected. Based on these findings, we proceeded to evaluate the influence of iodine equivalents on the formation of **1aa**. For this purpose, a mixture of ClCh–tartaric acid (1:2) was used as the reaction medium (Table 1, entries 9–18). Experimental data demonstrated that iodine concentrations between 0.5 and 1 equivalent promote the formation of **1aa** (see entries 12 and 13 of Table 1). Conversely, the use of two equivalents of iodine reduced the yield of **1aa** due to increased iodination of the 2-aminopyridine, resulting in the formation of 5-iodo-2-aminopyridine **2d**, which was isolated and characterized spectroscopically. The impact of 2-aminopyridine concentration on the reaction was then evaluated. The results showed that an excess of nucleophile significantly favored cyclization, increasing the yield of 39% and 60% in the production of **1aa** when using two and three equivalents of **2a**, respectively (Table 1, entries 15 and 16). This result suggests that using an excess of amine not only shifts the equilibrium toward adduct formation but also reduces the incidence of secondary reactions induced by excess iodine, such as iodination of the starting materials. In the absence of I_2_, only the 1,4-conjugate addition product of 2-aminopyridine to the chalcone is observed. This finding is consistent with the mechanism proposed and highlights the essential role of iodine in the cyclization.

**Scheme 2:**
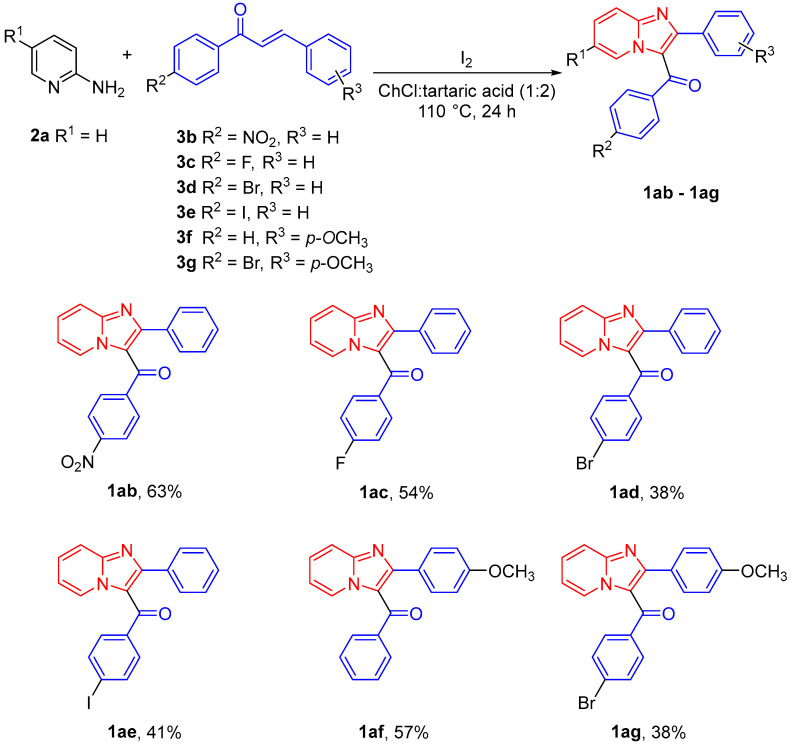
Isolated yields in the synthesis of substituted imidazo[1,2-*a*]pyridines **1ab**–**1ag**.

In a subsequent phase of the investigation, the influence of electron-donating and -withdrawing substituents on the optimized reaction conditions was evaluated. A series of 1,3-diphenylpropanones (**3b**–**3g**) reacted with 2-aminopyridine (**2a**), resulting in the formation of the corresponding imidazo[1,2-*a*]pyridines (**1ab**–**1ag**). The isolated yields for each derivative are presented in Scheme 2, confirming the influence of the electronic nature of the substituents on cyclization efficiency.

**Scheme 3:**
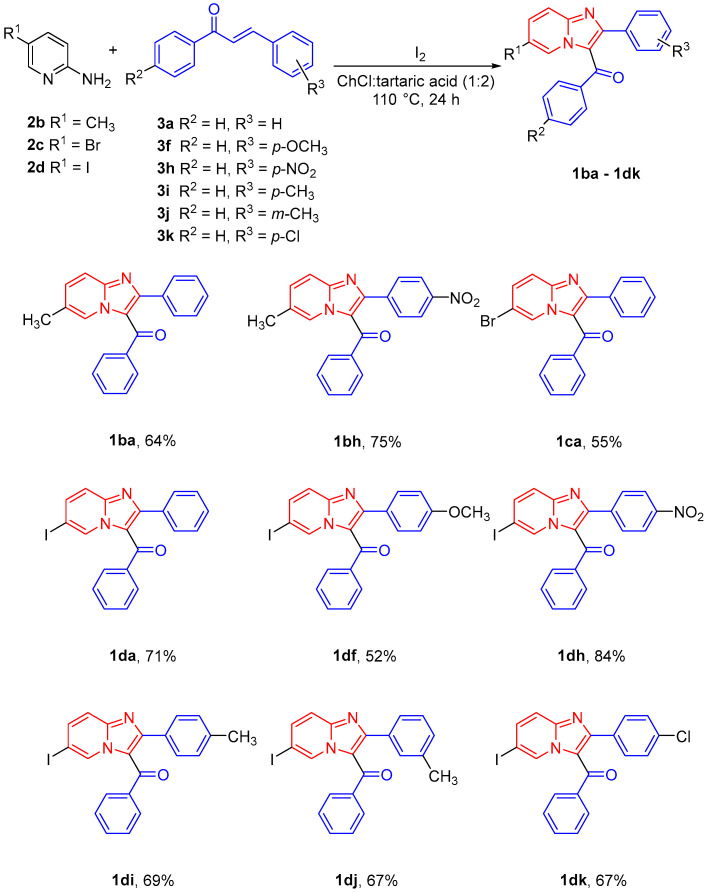
Isolated yields in the preparation of substituted imidazo[1,2-*a*]pyridines **1ba**–**1dk**.

In the case of derivative **3b**, containing a *p*-NO_2_ substituent, the formation of compound **1ab** was achieved in a 63% yield. In contrast, the halogenated chalcones **3c**–**3e** generated the products **1ac**–**1ae** in moderate yields due to the formation of by-products derived from competitive iodination of the starting materials. In the case of **3f**, with a *p*-OMe substituent on the β-aryl ring, a yield of 57% of **1af** was obtained. For the disubstituted chalcone **3g**, **1ag** was obtained in a yield of 38%, illustrating the relevance of the deactivating effect of bromine versus the donor character of methoxy, which limits the overall efficiency of the reaction. The *p*-OMe group acting as a substituent on ring β of chalcone **3f** increases the electron density at the C=C double bond, thereby facilitating the initial electrophilic addition of I_2_. Electronegative groups, such as the NO_2_ group in compound **3b**, increase the electrophilicity of the system and facilitate the subsequent nucleophilic attack on the iodinated intermediate. Therefore, the yields obtained reflect the effect of the substituents at different stages of the mechanism. The investigation next evaluated how substituents on the 2-aminopyridine ring **2** affect imidazo[1,2-*a*]pyridine **1** formation. The substituents used are listed in Scheme 3.

As expected, a pronounced reactivity dependence is related to the electronic and steric effects of substituted 2-aminopyridines **2** and chalcones **3**. In experiments conducted with 5-methyl-2-aminopyridine (**2b**) and **3a**, an increase in the nucleophilicity of the amino group was observed, attributable to the electron-donating effect of the methyl group, resulting in a higher yield of derivative **1ba** (64%). Notably, when the combination of **2b** with (*E*)-3-(4-nitrophenyl)-1-phenylprop-2-en-1-one (**3h**) was employed, a yield of 75% was achieved for **1bh**. This outcome confirms that the use of the activated nucleophile **2b** and the polarized electrophile **3h** promotes all the reaction steps towards the final heterocyclic compound. Bromine and iodine-substituted 2-aminopyridines (**2c** and **2d**) also yield imidazo[1,2-*a*]pyridines **1** efficiently, though the yield for **1ca** is limited to 55% due to the halogen’s electron-withdrawing effect on amino group reactivity. These results show that 5-bromo-2-aminopyridine **2c** is less nucleophilic than 5-iodo-2-aminopyridine **2d**. This observation can be attributed to the property of iodine to exhibit a weaker inductive effect and greater polarizability, which favours a higher electron density on the nitrogen of the amino group in **2d**. In contrast, in 5-bromo-2-aminopyridine **2c**, bromine has a deactivating effect, which reduces the nucleophilicity of the amino group.

**Scheme 4:**
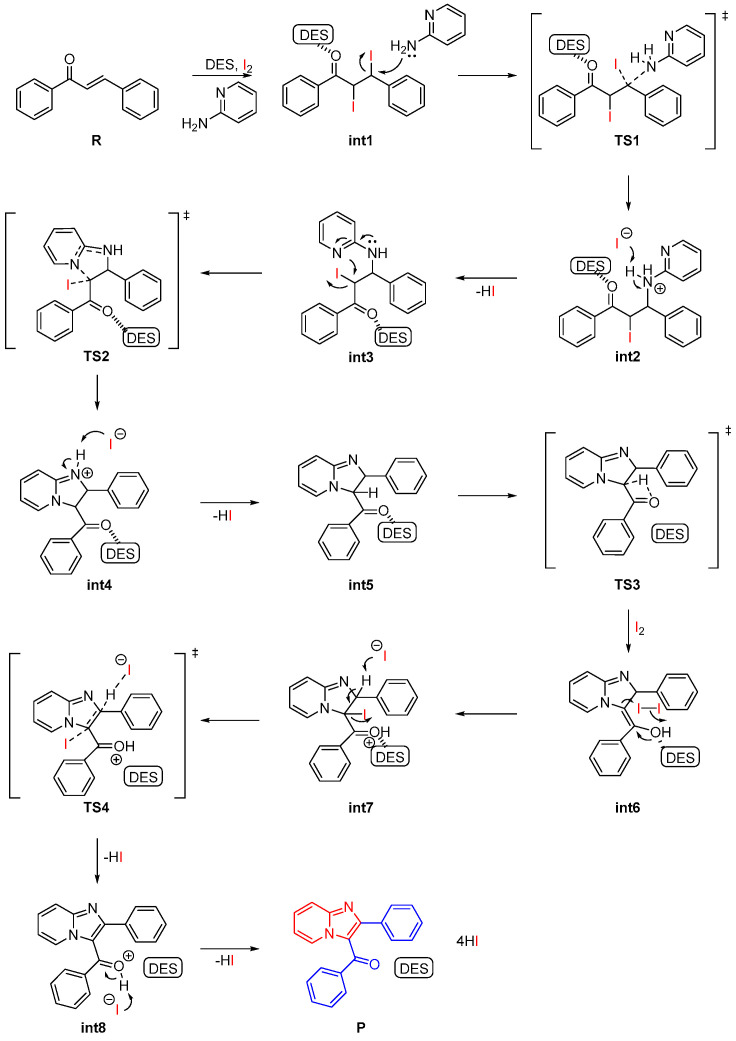
Proposed reaction mechanism for the synthesis of imidazo[1,2-*a*]pyridines **1** in DES ChCl–tartaric acid (1:2).

Scheme 4 shows the reaction mechanism for the synthesis of imidazo[1,2-*a*]pyridines **1** in the presence of the DES ChCl–tartaric acid (1:2).

**Figure 3:**
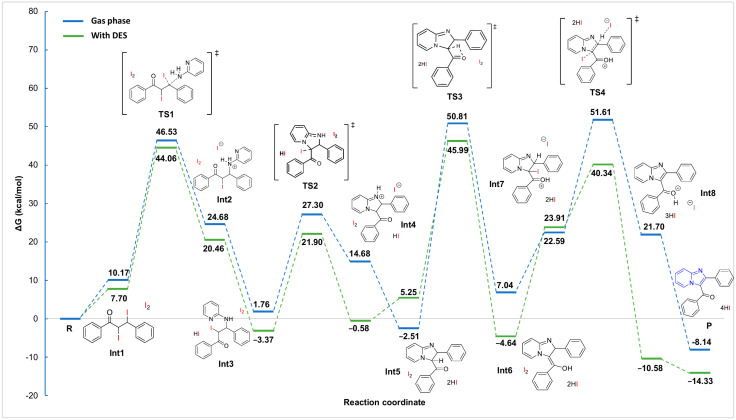
Gibbs free energy profiles for the formation of imidazopyridine **1aa**. The blue and green lines represent the reaction pathway in the gas phase and in the ChCl**–**tartaric acid (1:2) DES medium, respectively.

**Figure 4:**
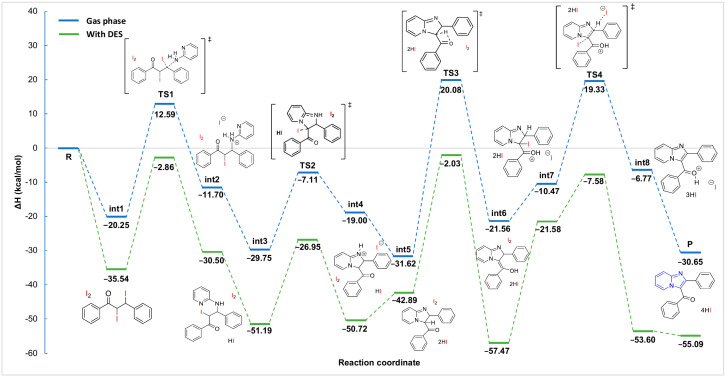
Enthalpy profiles for the formation of the imidazopyridine **1aa**. The blue and green lines represent the reaction pathway in the gas phase and in the ChCl**–**tartaric acid (1:2) DES medium, respectively.

Initially, the DES activates the carbonyl group of 1,3-diphenylpropenone (**3**) through acid–base interactions, enhancing the polarization of the conjugated system. Scheme 4 and Figure 3 and Figure 4 confirm that the addition of iodine to chalcone produces the di-iodination product. This intermediate 1 undergoes a nucleophilic attack by 2-aminopyridine (**2a**–**2d**), leading to a nucleophilic substitution process that provides the corresponding intermediate 2 in the reaction pathway (Scheme 4). The nucleophilicity of the -NH_2_ group is modulated by substituents at the 5-position of the 2-aminopyridine **2**, where electron-donating groups, such as methyl, enhance reactivity and yield, while halogen substituents exert inductive effects stabilizing intermediates and controlling the overall reaction efficiency. Subsequently, the intermediate adduct undergoes intramolecular cyclization, in which the aromatic nitrogen atom of the 2-aminopyridine (**2**) performs a nucleophilic substitution on the α-carbon to the activated carbonyl. In this step, the DES ChCl–tartaric acid (1:2), by increasing the acidity, facilitates protonation of intermediates and stabilizes them. Finally, iodine acts as an oxidant, promoting the aromatization of the ring, leading to the products imidazo[1,2-*a*]pyridine **1aa**–**1dk**.

To analyze the reaction mechanism and the effect of DES on the reaction, the potential energy surface was obtained in the gas phase and explicit DES medium (ChCl–tartaric acid (1:2)). The geometric structures of reagents (R), intermediates (int), transition states (TS), and product (P) were optimized at the PM6 level of theory using the GAUSSIAN09 program [55]. Previously, we used the same theoretical level to study the Michael addition reaction of pyrrole to maleimide, which agreed well with the experimental results [49]. In addition, Nakata and Maeba used the PM6 level of theory and obtained good results in the design of drugs and in materials science [56]. The optimized structures obtained for the reagents (R), intermediates (int), and product (P) were local minima with positive vibrational frequencies, while the transition states (TS) exhibited a first-order saddle point with a negative frequency. The nature of the transition states was confirmed by the calculation of the intrinsic reaction coordinate (IRC) [57,58].

Figure 3 and Figure 4 show the Gibbs free energy and enthalpy profiles for the reaction, respectively. The overall reaction R→P is spontaneous and exothermic: $∆G_{r}^{0}$ = −8.14 kcal/mol and $∆H_{r}^{0}$ = −30.65 kcal/mol for the gas phase and $∆G_{r}^{0}$ = −14.33 kcal/mol and $∆H_{r}^{0}$ = −55.09 kcal/mol in the DES ChCl–tartaric acid (1:2). The rate-limiting step of the reaction is the enolization of intermediate 5 (int5) to obtain intermediate 6 (int6) via proton transfer in transition state 3 (TS3): $∆G^{‡}$ = 53.32 kcal/mol and $∆H^{‡}$ = 51.70 kcal/mol for the gas phase and $∆G^{‡}$ = 40.74 kcal/mol and $∆H^{‡}$ = 40.86 kcal/mol in the DES ChCl–tartaric acid (1:2). Kaweetirawatt et al. showed that keto-enol tautomerization exhibits free energy barriers with $∆G^{‡}$ > 50 kcal/mol, suggesting that the energy cost is due to the hybridization change of the bonds involved [59]. Therefore, the reaction in DES is more favoured than in the gas phase, and the DES ChCl–tartaric acid (1:2) and iodine favor the enthalpy-driven binding of aminopyridine to chalcone in the reaction. It is interesting to mention that we have previously observed the same behavior in the aza-Michael addition of aniline to maleimide [60].

## 3. Materials and Methods

### 3.1. Experimental

Thin-layer chromatography (TLC) analyses were performed on commercial aluminum plates coated with a 0.25 mm layer of Merck silica gel 60F_254_ and visualized under UV light (254 nm) or iodine vapor. Column chromatography was performed using SiO_2_ (F60, 230–400 mesh). Infrared (IR) spectra were recorded on a Nicolet iS10 spectrometer (Thermo Scientific, Waltham, MA, USA) equipped with an ATR (attenuated total reflection) accessory. Characteristic absorption bands (ν_max_) are reported in wavenumbers (cm^−1^). Melting points were determined using a Melting Point Meter MPM-H2 (Paul Marienfeld GmbH & Co. KG, Lauda–Königshofen, Germany) apparatus and are reported uncorrected in degrees Celsius (°C). ^1^H and ^13^C NMR spectra were recorded on a Varian Mercury 400 MHz spectrometer (Varian Inc., Palo Alto, CA, USA) using CDCl_3_ solutions. Deuterated chloroform (CDCl_3_) was used as received, with chemical shifts (δ) reported in ppm relative to residual solvent signals [δ 7.26 (^1^H) and δ 77.16 (^13^C)]. Standard NMR abbreviations were used: s (singlet), d (doublet), t (triplet), m (multiplet). The synthesis of the 1,3-diphenylpropenones **3a**–**3k** was accomplished through the utilization of conventional methodologies employed for this objective [61]. The 2-Aminopyridines **2a**–**2d**, ChCl, tartaric acid, and iodine were obtained from commercial suppliers and used without further purification.

#### 3.1.1. Preparation of Deep Eutectic Solvent ChCl–Tartaric Acid (1:2)

Choline chloride (2.2 mmol) and tartaric acid (4.4 mmol) were mixed in a 10 mL round-bottomed flask and heated in a sand bath on a hot plate magnetic stirrer for 30 min at 130 °C until a clear liquid appeared. The colorless liquid was used directly for reactions without purification. Other deep eutectic solvents tested in this study were prepared similarly.

#### 3.1.2. Typical Experimental Procedure for the Synthesis of Imidazo[1,2-*a*]pyridines: Synthesis of **1aa**

In an open round-bottomed flask containing 1 g of ChCl/tartaric acid (1:2) (DES), 1 mmol of 1,3-diphenylpropenone (**3a**), 3 mmol of 2-aminopyridine (**2a**), and 1 mmol of iodine were added and stirred at 110 °C for 24 h. The reaction progress was monitored by TLC. After completion of the reaction, water was added, and the mixture was neutralized with a saturated aqueous solution of NaHCO_3_. The resulting mixture was washed with EtOAc (3 × 10 mL). The organic phases were combined and treated with anhydrous magnesium sulfate, filtered, and concentrated under reduced pressure to afford the crude reaction product. The product was purified by flash chromatography on silica gel, using a Hex/EtOAc mixture (7:3 or 1:1 *v*/*v*) as eluent.

#### 3.1.3. Characterization Data of Compounds

*Phenyl(2-phenylimidazo[1,2-a]pyridin-3-yl)methanone* (**1aa**): Yellow solid (60% yield); mp: 128–130 °C (lit.1 mp 130–132 °C); rf: 0.56 (Hex/EtOAc 1:1). ^1^H NMR (400 MHz, CDCl_3_): δ 9.55 (dd, *J* = 7.0, 1.2 Hz, 1H), 7.80 (d, *J* = 9.0 Hz, 1H), 7.56–7.49 (m, 3H), 7.32 (dd, *J* = 8.1, 1.6 Hz, 2H), 7.25 (t, *J* = 7.4 Hz, 1H), 7.16–7.05 (m, 6H); ^13^C NMR (101 MHz, CDCl_3_): δ 187.5, 155.1, 147.5, 138.8, 134.1, 131.9, 130.3, 129.7, 129.3, 128.4, 128.4, 127.9, 120.1, 117.6, 114.8; IR (ν/cm^−1^): 2360.44, 1600.63, 1386.57, 1326.79, 1222.65; LRMS (EI, 70 eV) *m*/*z* (%): 298 (100), 297 (72), 269 (54), 221 (48), 299 (23), 192 (17), 270 (15), 149 (14), 193 (8); HRMS (QTOF) *m*/*z* [M]+: Calculated for C_20_H_14_N_2_O 298.1106, found 298.1086.

*(4-Nitrophenyl)(2-phenylimidazo[1,2-a]pyridin-3-yl) methanone* (**1ab**): Yellow crystals (63% yield); mp: 172–174 °C; rf: 0.54 (Hex/EtOAc 1:1). ^1^H NMR (400 MHz, CDCl_3_): δ 9.65 (d, *J* = 6.9 Hz, 1H), 7.92 (d, *J* = 8.8 Hz, 3H), 7.66 (t, *J* = 7.9 Hz, 1H), 7.59 (d, *J* = 8.9 Hz, 2H), 7.27 (d, *J* = 6.8 Hz, 2H), 7.22 (t, *J* = 7.0 Hz, 1H), 7.16 (d, *J* = 7.4 Hz, 1H), 7.09 (t, *J* = 7.3 Hz, 2H); ^13^C NMR (101 MHz, CDCl_3_): δ 184.8, 155.6, 149.2, 147.5, 144.2, 133.0, 130.9, 130.4, 130.3, 129.3, 128.7, 128.2, 123.0, 119.9, 117.6, 115.8; IR (ν/cm^−1^): 3108.69, 1596.77, 1515.78, 1324.86, 1220.72; LRMS (EI, 70 eV) *m*/*z* (%): 343 (100), 207 (89), 342 (43), 281 (38), 221 (35), 314 (28), 344 (23), 268 (22), 208 (19), 192 (19); HRMS (QTOF) *m*/*z* [M]+: Calculated for C_20_H_13_N_3_O_3_ 343.0957, found 343.0951.

*(4-Fluorophenyl)(2-phenylimidazo[1,2-a]pyridin-3-yl) methanone* (**1ac**): White solid (54% yield); mp: 141 °C; rf: 0.5 (Hex/EtOAc 7:3). ^1^H NMR (400 MHz, CDCl_3_): δ 9.49 (d, *J* = 7.0 Hz, 1H), 7.84 (d, *J* = 9.0 Hz, 1H), 7.59–7.48 (m, 3H), 7.32 (d, *J* = 6.8 Hz, 2H), 7.21–7.16 (m, 1H), 7.15–7.07 (m, 3H), 6.76 (t, *J* = 8.7 Hz, 2H); ^13^C NMR (101 MHz, CDCl_3_): δ 185.8, 166.2, 163.7, 154.3, 147.2, 134.8, 134.8, 133.4, 132.2, 132.1, 130.3, 129.9, 128.8, 128.3, 128.1, 119.8, 117.4, 115.1, 115.0, 114.9; IR (ν/cm^−1^): 2923.56, 1602.56, 1384.64, 1330.64, 1224.58; LRMS (EI, 70 eV) *m*/*z* (%): 316 (100), 315 (75), 287 (65), 221 (31), 317 (26), 288 (18), 158 (16), 192 (16); HRMS (QTOF) *m*/*z* [M]+: Calculated for C_20_H_13_FN_2_O 316.1012, found 316.0999.

*(4-Bromophenyl)(2-phenylimidazo[1,2-a]pyridin-3-yl) methanone* (**1ad**): Yellow crystals (38% yield); mp: 116–119 °C; rf: 0.49 (Hex/EtOAc 7:3). ^1^H NMR (400 MHz, CDCl_3_): δ 9.53 (d, *J* = 7.0 Hz, 1H), 7.88 (d, *J* = 9.0 Hz, 1H), 7.61–7.55 (m, 1H), 7.35 (d, *J* = 8.6 Hz, 2H), 7.31 (d, *J* = 6.9 Hz, 2H), 7.22 (d, *J* = 8.6 Hz, 3H), 7.14 (t, *J* = 7.4 Hz, 3H); ^13^C NMR (101 MHz, CDCl_3_): δ 186.1, 154.4, 147.2, 137.4, 133.2, 131.2, 131.1, 130.4, 130.1, 128.9, 128.4, 128.2, 126.9, 119.8, 117.4, 115.3; IR (ν/cm^−1^): 2923.56, 2360.44, 1600.63, 1384.64, 1328.71, 1222.65; LRMS (EI, 70 eV) *m*/*z* (%): 376 (100), 378 (100), 377 (79), 375 (60), 221 (56), 349 (43), 347 (43), 192 (27), 207 (24), 379 (22), 268 (21), 193 (13), 348 (12), 350 (11), 189 (11), 188 (11); HRMS (QTOF) *m*/*z* [M]+: Calculated for C_20_H_13_BrN_2_O 376.0211, found 376.0200.

*(4-Iodophenyl)(2-phenylimidazo[1,2-a]pyridin-3-yl) methanone* (**1ae**): Pale yellow solid (41% yield); mp: 137–141 °C; rf: 0.57 (Hex/EtOAc 1:1). ^1^H NMR (400 MHz, CDCl_3_): δ 9.53 (d, *J* = 7.0 Hz, 1H), 7.87 (d, *J* = 9.0 Hz, 1H), 7.61–7.55 (m, 1H), 7.43 (d, *J* = 8.5 Hz, 2H), 7.32–7.28 (m, 2H), 7.20 (t, *J* = 8.6 Hz, 3H), 7.16–7.10 (m, 3H); ^13^C NMR (101 MHz, CDCl_3_): δ 186.3, 154.6, 147.2, 138.0, 137.1, 133.3, 131.0, 130.4, 130.0, 128.8, 128.5, 128.1, 119.8, 117.4, 115.2, 99.3; IR (ν/cm^−1^): 2923.56, 1610.27, 1392.35, 1326.79, 1226.5; LRMS (EI, 70 eV) *m*/*z* (%): 207 (100), 424 (81), 281 (43), 423 (33), 221 (23), 395 (23), 208 (21), 425 (18), 253 (13); HRMS (QTOF) *m*/*z* [M]+: Calculated for C_20_H_13_IN_2_O 424.0073, found 424.0073.

*(2-(4-Methoxyphenyl)imidazo[1,2-a]pyridin-3-yl)(phenyl) methanone* (**1af**): Brown oil (57% yield); rf: 0.56 (Hex/EtOAc 7:3). ^1^H NMR (400 MHz, CDCl_3_): δ 9.52 (d, *J* = 7.0 Hz, 1H), 7.86 (d, *J* = 9.0 Hz, 1H), 7.55 (dd, *J* = 15.1, 7.5 Hz, 3H), 7.29 (t, *J* = 8.6 Hz, 3H), 7.12 (q, *J* = 7.5 Hz, 3H), 6.62 (d, *J* = 8.9 Hz, 2H), 3.72 (s, 3H); ^13^C NMR (101 MHz, CDCl_3_): δ 187.4, 160.1, 153.7, 146.9, 138.5, 132.1, 131.7, 129.9, 129.7, 128.4, 128.0, 125.6, 119.6, 117.1, 115.0, 113.5, 55.4; IR (ν/cm^−1^): 2931.27, 1596.77, 1388.5, 1328.71, 1251.58; LRMS (EI, 70 eV) *m*/*z* (%): 328 (100), 327 (43), 299 (38), 207 (35), 329 (25), 251 (20), 281 (15), 208 (14), 164 (13), 300 (10); HRMS (QTOF) *m*/*z* [M]+: Calculated for C_21_H_16_N_2_O_2_ 328.1212, found 328.1208.

*(4-Bromophenyl)(2-(4-methoxyphenyl)imidazo[1,2-a]pyridin-3-yl)methanone* (**1ag**): Yellow solid (38% yield); mp: 108–110 °C; rf: 0.38 (Hex/EtOAc 7:3). ^1^H NMR (400 MHz, CDCl_3_): δ 9.52 (d, *J* = 6.9 Hz, 1H), 7.80 (d, *J* = 9.0 Hz, 1H), 7.57–7.51 (m, 1H), 7.36 (d, *J* = 8.6 Hz, 2H), 7.27–7.20 (m, 4H), 7.09 (t, *J* = 6.9 Hz, 1H), 6.65 (d, *J* = 8.9 Hz, 2H), 3.75 (s, 3H); ^13^C NMR (101 MHz, CDCl_3_): δ 186.1, 160.2, 154.9, 147.6, 137.6, 131.7, 131.2, 131.1, 129.7, 128.4, 126.5, 126.0, 119.6, 117.4, 114.9, 113.6, 55.5; IR (ν/cm^−1^): 2923.56, 1602.56, 1398.14, 1241.93, 1224.58; LRMS (EI, 70 eV) *m*/*z* (%): 207 (100), 281 (42), 208 (24), 406 (24), 408 (22), 209 (14), 253 (14), 407 (12); HRMS (QTOF) *m*/*z* [M]+: Calculated for C_21_H_15_BrN_2_O_2_ 406.0317, found 406.0325.

*(6-Methyl-2-phenylimidazo[1,2-a]pyridin-3-yl)(phenyl) methanone* (**1ba**): Pale yellow solid (64% yield); mp: 165 °C (mp 163–164 °C); rf: 0.55 (Hex/EtOAc 7:3). ^1^H NMR (400 MHz, CDCl_3_): δ 9.35 (s, 1H), 7.71 (d, *J* = 8.9 Hz, 1H), 7.49 (d, *J* = 6.9 Hz, 2H), 7.37 (d, *J* = 9.0 Hz, 1H), 7.30 (d, *J* = 6.6 Hz, 2H), 7.23 (t, *J* = 7.4 Hz, 1H), 7.13–7.03 (m, 5H), 2.43 (s, 3H); ^13^C NMR (101 MHz, CDCl_3_): δ 187.4, 154.6, 146.3, 138.8, 134.0, 132.3, 131.8, 130.3, 129.7, 128.3, 127.8, 126.2, 124.8, 119.9, 116.7, 18.6; IR (ν/cm^−1^): 3064.33, 2360.44, 1600.63, 1388.5, 1326.79, 1236.15; LRMS (EI, 70 eV) *m*/*z* (%): 312 (100), 311 (68), 283 (57), 235 (50), 313 (25), 156 (19), 284 (15), 207 (9), 236 (9); HRMS (QTOF) *m*/*z* [M]+: Calculated for C_21_H_16_N_2_O 312.1263, found 312.1255.

*(6-Methyl-2-(4-nitrophenyl)imidazo[1,2-a]pyridin-3-yl)(phenyl)methanone* (**1bh**): Yellow crystals (75% yield); mp: 198–199 °C; rf: 0.37 (Hex/EtOAc 1:1). ^1^H NMR (400 MHz, CDCl_3_): δ 9.32 (s, 1H), 7.95 (d, *J* = 8.9 Hz, 2H), 7.82 (d, *J* = 9.2 Hz, 1H), 7.54–7.46 (m, 5H), 7.35–7.29 (m, 1H), 7.13 (t, *J* = 7.8 Hz, 2H), 2.48 (s, 3H); ^13^C NMR (101 MHz, CDCl_3_): δ 186.8, 150.3, 147.6, 145.8, 139.7, 138.4, 133.5, 132.8, 131.1, 129.7, 128.3, 126.2, 126.1, 123.1, 120.4, 116.7, 18.7; IR (ν/cm^−1^): 2917.77, 1604.48, 1513.85, 1342.21, 1330.64, 1236.15; LRMS (EI, 70 eV) *m*/*z* (%): 357 (100), 207 (72), 281 (39), 328 (34), 327 (34), 356 (33), 280 (27), 282 (26), 358 (24), 298 (17), 235 (15), 208 (15), 234 (13), 283 (12), 326 (11), 310 (11), 209 (11), 205 (9); HRMS (QTOF) *m*/*z* [M]+: Calculated for C_21_H_15_N_3_O_3_ 357.1113, found 357.1107.

*(6-Bromo-2-phenylimidazo[1,2-a]pyridin-3-yl)(phenyl) methanone* (**1ca**): White solid (55% yield); mp: 137–139 °C (mp 135–137 °C); rf: 0.64 (Hex/EtOAc 1:1). ^1^H NMR (400 MHz, CDCl_3_): δ 9.70 (s, 1H), 7.69 (d, *J* = 9.5 Hz, 1H), 7.58 (d, *J* = 9.4 Hz, 1H), 7.50 (d, *J* = 8.4 Hz, 2H), 7.34–7.24 (m, 3H), 7.18–7.04 (m, 5H); ^13^C NMR (101 MHz, CDCl_3_): δ 187.3, 154.9, 145.8, 138.2, 133.5, 132.6, 132.1, 130.2, 129.6, 128.5, 128.4, 127.9, 120.1, 118.0, 109.4; IR (ν/cm^−1^): 2919.7, 1612.2, 1382.71, 1328.71, 1216.86; LRMS (EI, 70 eV) *m*/*z* (%): 376 (100), 378 (99), 377 (79), 375 (60), 349 (38), 347 (38), 297 (37), 299 (35), 301 (34), 379 (21), 192 (20), 148 (18); HRMS (QTOF) *m*/*z* [M]+: Calculated for C_20_H_13_BrN_2_O 376.0211, found 298.9811

*(6-Iodo-2-phenylimidazo[1,2-a]pyridin-3-yl)(phenyl) methanone* (**1da**): Yellow solid (71% yield); mp: 160–161 °C; rf: 0.52 (Hex/EtOAc 1:1). ^1^H NMR (400 MHz, CDCl_3_): δ 9.80 (s, 1H), 7.70 (dd, *J* = 9.3, 1.8 Hz, 1H), 7.58 (d, *J* = 9.2 Hz, 1H), 7.50 (dd, *J* = 8.3, 1.3 Hz, 2H), 7.31 (dd, *J* = 8.3, 1.4 Hz, 2H), 7.28–7.25 (m, 1H), 7.17–7.06 (m, 5H); ^13^C NMR (101 MHz, CDCl_3_): δ 187.4, 154.7, 146.0, 138.3, 137.3, 133.6, 133.2, 132.2, 130.3, 129.7, 128.6, 128.0, 119.8, 118.5, 77.9; IR (ν/cm^−1^): 3039.26, 2362.37, 1598.7, 1380.78, 1332.57, 1247.72; LRMS (EI, 70 eV) *m*/*z* (%): 424 (100), 423 (43), 347 (27), 395 (25), 425 (23), 297 (22), 207 (21), 267 (13), 192 (10), 212 (10); HRMS (QTOF) *m*/*z* [M]+: Calculated for C_20_H_13_IN_2_O 424.0073, found 424.0069.

*(6-Iodo-2-(4-methoxyphenyl)imidazo[1,2-a]pyridin-3-yl)(phenyl)methanone* (**1df**): Brown solid (52% yield); mp: 135–139 °C; rf: 0.47 (Hex/EtOAc 1:1). ^1^H NMR (400 MHz, CDCl_3_) δ 9.77 (s, 1H), 7.74 (dd, *J* = 9.2, 1.7 Hz, 1H), 7.66 (d, *J* = 10.1 Hz, 1H), 7.53 (d, *J* = 7.0 Hz, 2H), 7.36–7.25 (m, 4H), 7.15 (t, *J* = 7.8 Hz, 2H), 6.63 (d, *J* = 8.9 Hz, 2H), 3.73 (s, 3H); ^13^C NMR (101 MHz, CDCl_3_): δ 187.3, 160.3, 153.1, 145.3, 138.0, 137.9, 133.1, 132.4, 131.8, 129.7, 128.1, 124.9, 119.2, 117.9, 113.7, 78.2, 55.4; IR (ν/cm^−1^): 3046.98, 1602.56, 1382.71, 1330.64, 1245.79; LRMS (EI, 70 eV) *m*/*z* (%): 454 (100), 207 (39), 455 (24), 453 (23), 425 (23), 281 (15), 377 (13), 227 (13), 327 (11), 208 (8); HRMS (QTOF) *m*/*z* [M]+: Calculated for C_21_H_15_IN_2_O_2_ 454.0178, found 454.0169.

*(6-Iodo-2-(4-nitrophenyl)imidazo[1,2-a]pyridin-3-yl) (phenyl)methanone* (**1dh**): Pale green solid (84% yield); mp: 218–220 °C; rf: 0.37 (Hex/EtOAc 1:1). ^1^H NMR (400 MHz, CDCl_3_): δ 9.76 (s, 1H), 7.94 (d, *J* = 9.0 Hz, 2H), 7.75 (d, *J* = 9.3 Hz, 1H), 7.60 (d, *J* = 9.3 Hz, 1H), 7.51–7.46 (m, 4H), 7.33 (t, *J* = 7.4 Hz, 1H), 7.16–7.11 (m, 2H); ^13^C NMR (101 MHz, CDCl_3_): δ 186.9, 151.4, 147.5, 146.0, 140.1, 138.1, 137.9, 133.2, 132.9, 131.0, 129.7, 128.3, 123.0, 120.3, 118.7, 78.8; IR (ν/cm^−1^): 3050.83, 1621.84, 1509.99, 1334.5, 1216.86; LRMS (EI, 70 eV) *m*/*z* (%): 207 (100), 469 (80), 281 (40), 439 (24), 208 (21), 470 (18), 468 (17), 440 (15), 191 (15), 392 (14), 282 (14), 209 (13), 253 (12), 267 (11), 296 (11), 295 (10), 283 (10); HRMS (QTOF) *m*/*z* [M]+: Calculated for C_20_H_12_IN_3_O_3_ 468.9923, found 468.9921.

*(6-Iodo-2-(p-tolyl)imidazo[1,2-a]pyridin-3-yl)(phenyl) methanone* (**1di**): Pale yellow solid (69% yield); mp: 149–152 °C; rf: 0.36 (Hex/EtOAc 1:1). ^1^H NMR (400 MHz, CDCl_3_): δ 9.76 (s, 1H), 7.66 (dd, *J* = 9.2, 1.7 Hz, 1H), 7.54 (d, *J* = 8.3 Hz, 1H), 7.50 (d, *J* = 7.0 Hz, 2H), 7.27 (t, *J* = 7.4 Hz, 1H), 7.18 (d, *J* = 8.3 Hz, 2H), 7.10 (d, *J* = 8.3 Hz, 2H), 6.87 (d, *J* = 7.8 Hz, 2H), 2.21 (s, 3H); ^13^C NMR (101 MHz, CDCl_3_): δ 187.4, 154.7, 146.0, 138.5, 138.3, 137.1, 133.1, 132.0, 130.5, 130.2, 129.7, 128.6, 127.9, 119.6, 118.4, 77.7, 21.3; IR (ν/cm^−1^): 2921.63, 1612.2, 1471.42, 1380.78, 1326.79, 1214.93; LRMS (EI, 70 eV) *m*/*z* (%): 207 (100), 438 (78), 281 (49), 437 (32), 208 (21), 409 (20), 439 (18), 282 (16), 311 (13), 209 (13), 191 (13), 253 (12); HRMS (QTOF) *m*/*z* [M]+: Calculated for C_21_H_15_IN_2_O 438.0229, found 438.0225.

*(6-Iodo-2-(m-tolyl)imidazo[1,2-a]pyridin-3-yl)(phenyl) methanone* (**1dj**): Pale yellow solid (67% yield); mp: 97–99 °C; rf: 0.51 (Hex/EtOAc 1:1). ^1^H NMR (400 MHz, CDCl_3_): δ 9.79 (s, 1H), 7.68 (dd, *J* = 9.2, 1.7 Hz, 1H), 7.56 (d, *J* = 9.3 Hz, 1H), 7.50 (d, *J* = 7.0 Hz, 2H), 7.27 (t, *J* = 7.4 Hz, 1H), 7.14–7.06 (m, 4H), 7.01–6.91 (m, 2H), 2.11 (s, 3H); ^13^C NMR (101 MHz, CDCl_3_): δ 187.5, 154.9, 146.0, 138.5, 137.6, 137.2, 133.3, 133.2, 132.1, 131.1, 129.5, 129.4, 127.9, 127.4, 119.8, 118.4, 77.8, 21.1; IR (ν/cm^−1^): 3037.34, 2360.44, 1596.77, 1369.21, 1332.57, 1245.79; LRMS (EI, 70 eV) *m*/*z* (%): 207 (100), 438 (81), 281 (49), 437 (38), 409 (23), 208 (21), 439 (18), 282 (16), 209 (13), 155 (13), 191 (13), 311 (12), 253 (12), 283 (10); HRMS (QTOF) *m*/*z* [M]+: Calculated for C_21_H_15_IN_2_O 438.0229, found 438.0225.

*(2-(4-Chlorophenyl)-6-iodoimidazo[1,2-a]pyridin-3-yl)(phenyl)methanone* (**1dk**): White solid (67% yield); mp: 168 °C; rf: 0.58 (Hex/EtOAc 7:3). ^1^H NMR (400 MHz, CDCl_3_): δ 9.77 (s, 1H), 7.73 (d, *J* = 9.3 Hz, 1H), 7.62 (d, *J* = 9.3 Hz, 1H), 7.50 (d, *J* = 7.1 Hz, 2H), 7.35 (t, *J* = 7.5 Hz, 1H), 7.25 (d, *J* = 8.5 Hz, 2H), 7.18–7.13 (m, 2H), 7.07 (d, *J* = 8.8 Hz, 2H); ^13^C NMR (101 MHz, CDCl_3_): δ 187.1, 152.6, 145.7, 138.1, 137.8, 135.1, 133.2, 132.6, 131.6, 131.5, 129.7, 128.3, 128.2, 119.7, 118.3, 78.4; IR (ν/cm^−1^): 3043.12, 1600.63, 1380.78, 1328.71, 1214.93; LRMS (EI, 70 eV) *m*/*z* (%): 458 (100), 460 (36), 459 (32), 207 (32), 457 (31), 429 (23), 381 (23), 331 (20), 281 (13), 165 (11), 191 (11), 295 (10); HRMS (QTOF) *m*/*z* [M]+: Calculated for C_20_H_12_ClIN_2_O 457.9683, found 457.9683.

### 3.2. Calculation Methods

The equilibrium geometries of all species involved in the reaction, including the reagents 2-aminopyridine (**2a**), chalcone (**3a**), iodine, the ChCl/tartaric acid deep eutectic solvent (DES), the transition states (TS1–TS4), the intermediates (Int1–Int8), and the final product (P), were obtained at the PM6 level of theory using the Gaussian 09 program [62]. PM6 has advantages over other semi-empirical methods for obtaining some properties ($∆H_{r}^{0}$, geometries, H bonds, etc.) [55]. Calculations were carried out both in the gas phase and in the presence of the ChCl–tartaric acid as the deep eutectic solvent (DES) modeled explicitly using a 1:2 molar ratio of chloride to tartaric acid in agreement with the experimental conditions. The frequency analysis was conducted for the optimized geometries of the species, verifying that all frequencies were positive and that only one negative frequency was obtained for the transition states. Additionally, intrinsic reaction coordinate (IRC) calculations were made at the same theoretical level, confirming that the transition state represents the highest energy structure along the minimum energy pathway connecting reagents and products.

## 4. Conclusions

We have outlined a sustainable, metal-free protocol for synthesizing various imidazo[1,2-*a*]pyridines containing either electron-donating or electron-withdrawing substituents. This method involves reacting different 2-aminopyridines with chalcones in a DES ChCl–tartaric acid (1:2):I_2_ catalytic system. The acidity and polarity of the DES are pivotal in activating the carbonyl group and stabilizing the intermediates. This efficiently facilitates the amino-functionalization of the chalcone, the cyclization, and the oxidative aromatization. Research has shown that the electronic and steric properties of the substituents, in both the aminopyridine and the chalcone, have a significant impact on reaction efficiency and product yield. Furthermore, iodine acts as an effective electrophile and oxidant, promoting ring closure and aromatization without the need for metal catalysts or volatile organic solvents. This methodology underscores the significant potential of the DES ChCl–tartaric acid (1:2):I_2_ catalytic system in the synthesis of imidazo[1,2-*a*]pyridines.

## Figures and Tables

**Table 1:** Imidazo[1,2-*a*]pyridine (**1aa**) synthesis optimization using different DESs ^a^.

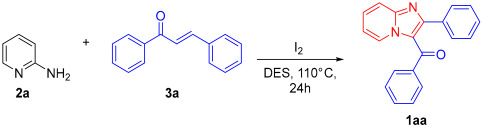				
Entry	DES	2a (Equiv)	I_2_ (Equiv)	1aa Yield (%) ^b^
1	ChCl–oxalic acid (1:2)	1	1	traces
2	ChCl–malonic acid (1:2)	1	1	traces
3	ChCl–tartaric acid (1:2)	1	1	23
4	ChCl–*p*-TsOH(1:1)	1	1	19
5	ChCl–glycerol (1:2)	1	1	15
6	ChCl–urea (1:2)	1	1	traces
7	ChCl–DMU (1:2)	1	1	11
8	Glycerol–K_2_CO_3_ (2:1)	1	1	N.D. ^c^
9	ChCl–ZnCl_2_ (1:2)	1	1	N.D. ^c^
10	ChCl–tartaric acid (1:2) ^d^	1	1	Traces
11	ChCl–tartaric acid (1:2) ^e^	1	1	18
12	ChCl–tartaric acid (1:2)	1	1	23
13	ChCl–tartaric acid (1:2)	1	0.5	22
14	ChCl–tartaric acid (1:2)	1	2	20
15	ChCl–tartaric acid (1:2)	2	1	39
16	ChCl–tartaric acid (1:2)	3	1	60
17	ChCl–tartaric acid (1:2)	4	1	58
18	ChCl–tartaric acid (1:2)	5	1	41

^a^ Reaction conditions: **3a** (1.0 mmol, 0.208 g), **2a**, I_2_ (**2a** and I_2_ were used in the amounts indicated in the table.), DES (1.0 mL), 110 °C, 24 h. ^b^ Isolated yield after column chromatography (silica, hexane/EtOAc). ^c^ Not detected. ^d^ Reaction time of 3 h. ^e^ Reaction time of 8 h. Abbreviations: ChCl: choline chloride; *p*-TsOH: *p*-toluenesulfonic acid; DMU: dimethyl urea.

## Data Availability

Data are contained within the article and Supplementary Materials.

## Supplementary Materials

The following supporting information can be downloaded at: https://www.mdpi.com/article/10.3390/molecules31091416/s1.
