# Supplementary material for: I2 and the Deep Eutectic Solvent ChCl–Tartaric Acid Promote the Addition–Oxidative Cyclization of 2-Aminopyridines and Chalcones to Obtain Imidazo[1,2-a]pyridines
Source: Molecules. 2026 Apr 24;31(9):1416. doi: 10.3390/molecules31091416 (PMC13164598; doi:10.3390/molecules31091416)
Supplement: Supplementary file 1 [file molecules-31-01416-s001.zip › molecules-4198741-supplementary.pdf]

SUPPORTING INFORMATION

I_2 and Deep Eutectic Solvent ChCl: tartaric acid promote the addition-oxidative cyclization of 2-aminopyridines and chalcones to obtain imidazo[1,2-*a*]pyridines

Juan Lopez de Leon,[1] Nayely Melissa Cruces Velazco,[1] Arlette Richaud,[2] Francisco Méndez,[3,*] Diego A. Alonso[4,*] and Claudia Araceli Contreras-Celedón[1,*]

[1] Departamento de Síntesis Orgánica, Instituto de Investigaciones Químico Biológicas, Universidad Michoacana de San Nicolás de Hidalgo, Morelia 58030, México

[2] Conditions Extrêmes et Matériaux: Haute Température et Irradiation, CEMHTI - UPR3079 CNRS. 1 Avenue de la Recherche Scientifique, 45100 Orléans, France

[3] Departamento de Química, División de Ciencias Básicas e Ingeniería Universidad Autónoma Metropolitana-Iztapalapa, Ciudad de México, 09340, México.

[4] Department of Organic Chemistry and Organic Synthesis Institute (ISO), Alicante University, Apdo. 99, 03080 Alicante, Spain.

*****Correspondence: claudia.contreras@umich.mx (C. A. C. C.); diego.alonso@ua.es (D. A. A.); fm@xanum.uam.mx (F. M.).

Table of contents

General S-1

Experimental procedures and characterization data S-1

Preparation of 1,3-diphenylpropenones (Chalcones) S-1

Deep eutectic solvent preparation (ChCl-tartaric acid, 1:2) S-2

Typical experimental procedure for the synthesis of **1aa** S-2

NMR spectra S-3

Mechanistic proposal S-20

Gas Phase Energies and Cartesian Coordinates of

2-aminopyridine S-21
Chalcone S-22
Intermediary 1 (**Int1**) S-24
Transition state 1 (**TS1**) S-26
Intermediary 2 (**Int2**) S-28
Intermediary 3 (**Int3**) S-30
Transition state 2 (**TS2**) S-32
Intermediary 4 (**Int4**) S-34
Intermediary 5 (**Int5**) S-36
Transition state 3 (**TS3**) S-38
Intermediary 6 (**Int6**) S-40
Intermediary 7 (**Int7**) S-42
Transition state 4 (**TS4**) S-44
Intermediary 8 (**int8**) S-46
Product (**P**) S-48
DES: ChCl:Tartaric acid (1:2) S-50

IRC Graphs of Transition State in Gas Phase

IRC Transition state 1 (**TS1**) S-53
IRC Transition state 2 (**TS2**) S-55
IRC Transition state 3 (**TS3**) S-57
IRC Transition state 4 (**TS4**) S-59

Energies and Cartesian Coordinates in DES of

Intermediary 1 (**Int1**) S-61
Transition state 1 (**TS1**) S-65
Intermediary 2 (**Int2**) S-69
Intermediary 3 (**Int3**) S-74

Transition state 2 (**TS2**) S-79
Intermediary 4 (**Int4**) S-84
Intermediary 5 (**Int5**) S-89
Transition state 3 (**TS3**) S-94
Intermediary 6 (**Int6**) S-99
Intermediary 7 (**Int7**) S-104
Transition state 4 (**TS4**) S-109
Intermediary 8 (**int8**) S-114
Product (**P**) S-119

IRC Graphs of Transition State in DES

IRC Transition state 1 (**TS1**) S-124
IRC Transition state 2 (**TS2**) S-129
IRC Transition state 3 (**TS3**) S-134
IRC Transition state 4 (**TS4**) S-139

General

Thin-layer chromatography (TLC) analyses were performed on commercial aluminum plates coated with a 0.25 mm layer of Merck silica gel $60F_{254}$, visualized under UV light (254 nm) or iodine vapor. Column chromatography was performed using SiO_2 (F60, 230-400 mesh). Infrared (IR) spectra were recorded on a Nicolet iS10 spectrometer (Thermo Scientific, Waltham, MA, USA) equipped with an ATR (Attenuated Total Reflection) accessory. Characteristic absorption bands (ν_{max}) are reported in wavenumbers (cm^{-1}). Melting points were determined using a Melting Point Meter MPM-H2 (Paul Marienfeld GmbH & Co. KG, Lauda-Königshofen, Germany). ^1H and ^{13}C NMR spectra were recorded on a Varian Mercury 400 MHz spectrometer (Varian Inc., Palo Alto, CA, USA) using $CDCl_3$ solutions. Deuterated chloroform ($CDCl_3$) was used as received, with chemical shifts (δ) reported in ppm relative to residual solvent signals [δ 7.26 (^1H) and δ 77.16 (^{13}C)]. Standard NMR abbreviations were used: s (singlet), d (doublet), t (triplet), m (multiplet). The synthesis of the 1,3-diphenylpropenones **3a** - **3k**, was accomplished through the utilization of conventional methodologies employed for this objective. 2-Aminopyridines **2a**-**2d**, ChCl, tartaric acid, and iodine were obtained from commercial suppliers and used without further purification.

Experimental procedures and characterization data

Preparation of 1,3-diphenylpropenones (Chalcones)

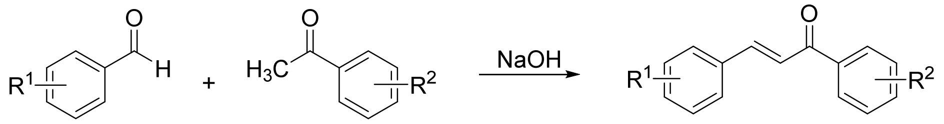

A series of 1,3-diphenylpropenones were synthesized from commercially available benzaldehydes and acetophenones according to a procedure described by Daniel R. Palleros.

In a typical reaction, 5 mmol of the benzaldehyde and 5 mmol of the acetophenone were added to a porcelain mortar. Then, 200 mg of solid NaOH was added, and the mixture was ground with a pestle for 5-10 minutes. The reaction mixtures solidified upon grinding. The crude reaction mixture was washed with water, vacuum filtered, and air-dried. Subsequent recrystallization from the appropriate solvent afforded the pure products in 81–94% yield.

Deep eutectic solvent preparation (choline chloride-tartaric acid, 1:2):

Choline chloride (2.2 mmol) and tartaric acid (4.4 mmol) were mixed in a 10 mL round-bottomed flask and heated in a sand bath on a hot plate magnetic stirrer for 30 min at 130 °C until a clear liquid appeared; the colorless liquid was used directly for the reactions without purification. Other deep eutectic solvents tested in this study were prepared in a similar way.

Typical experimental procedure for the synthesis of 2-imidazo[1,2-a]pyridines: Synthesis of ***1a.***

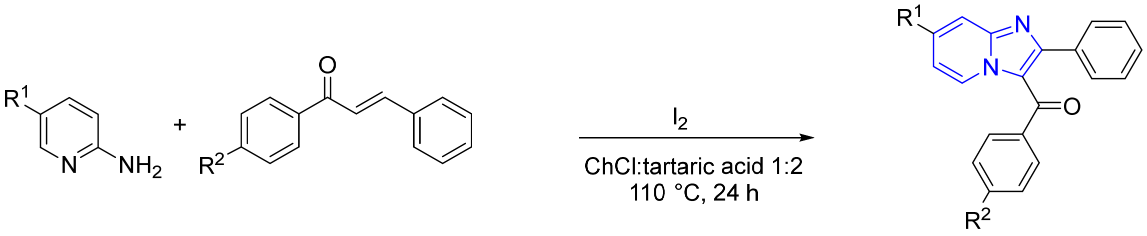

2a R^1 = H **3a** R^2 = H, R^3 = H **1a** R^1 = H, R^2 = H, R^3 = H

In an open round-bottom flask, 1 g of DES ChCl/tartaric acid 1:2, 1,3-diphenylpropenone (**3a**, 1 mmol), 2-aminopyridine (**2a**, 3 mmol), and iodine (1 mmol) were added and stirred at 110 °C for 24 h. The reaction progress was monitored by TLC. After completion of the reaction, water was added and neutralized with a saturated aqueous solution of $NaHCO_3$, the extraction was performed with EtOAc (3 × 10 mL). The combined organic phases were dried over anhydrous magnesium sulfate, filtered, and concentrated under reduced pressure to afford the crude product. The product was purified by flash chromatography on silica gel, using a Hex/EtOAc mixture (7:3 or 1:1 v/v) as eluent.

NMR Spectra

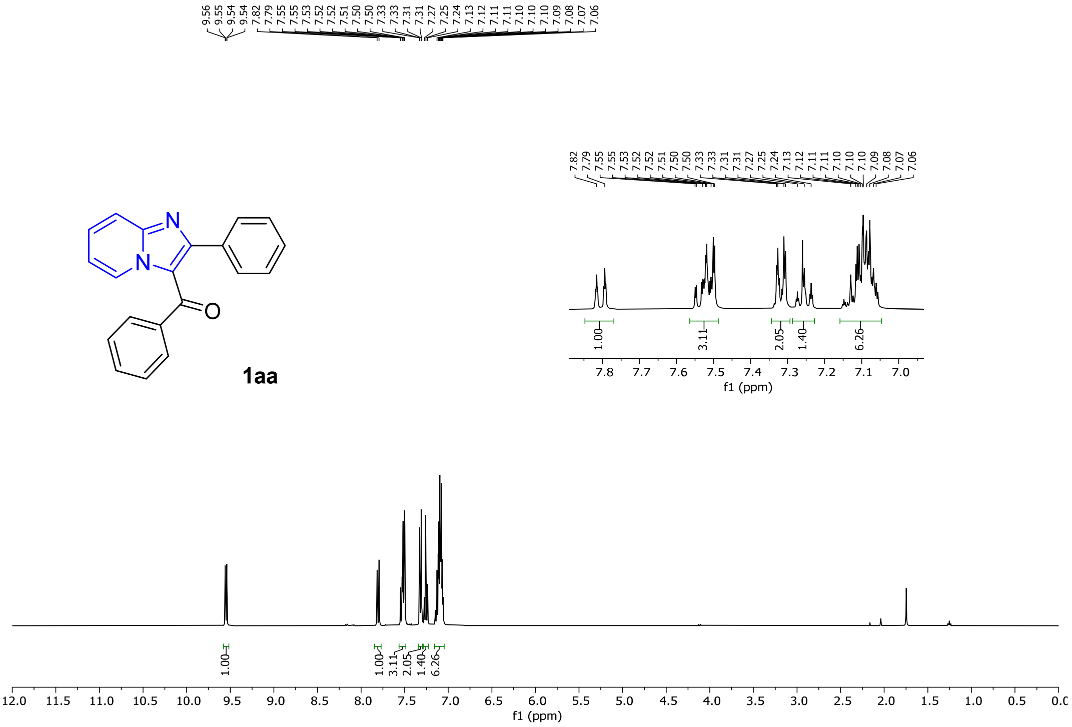

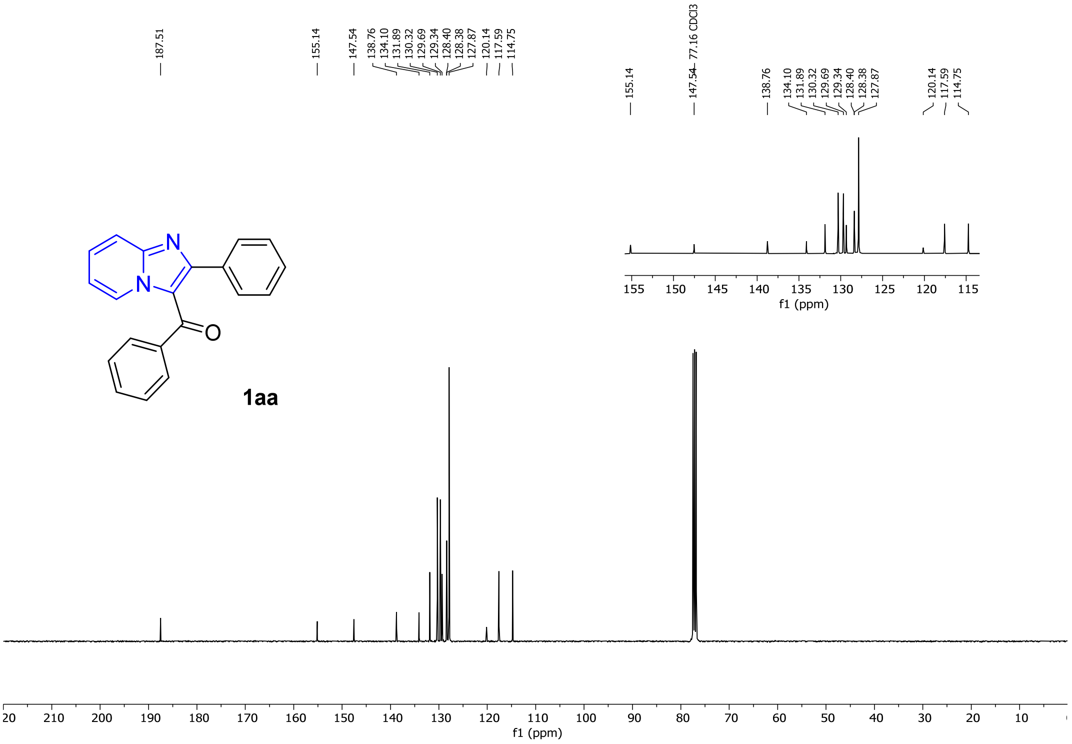

^{1}H NMR (400 MHz, $CDCl_3$), ^{13}C NMR (101 MHz, $CDCl_3$) Phenyl(2-phenylimidazo[1,2-*a*]pyridin-3-yl)methanone (**1aa**)

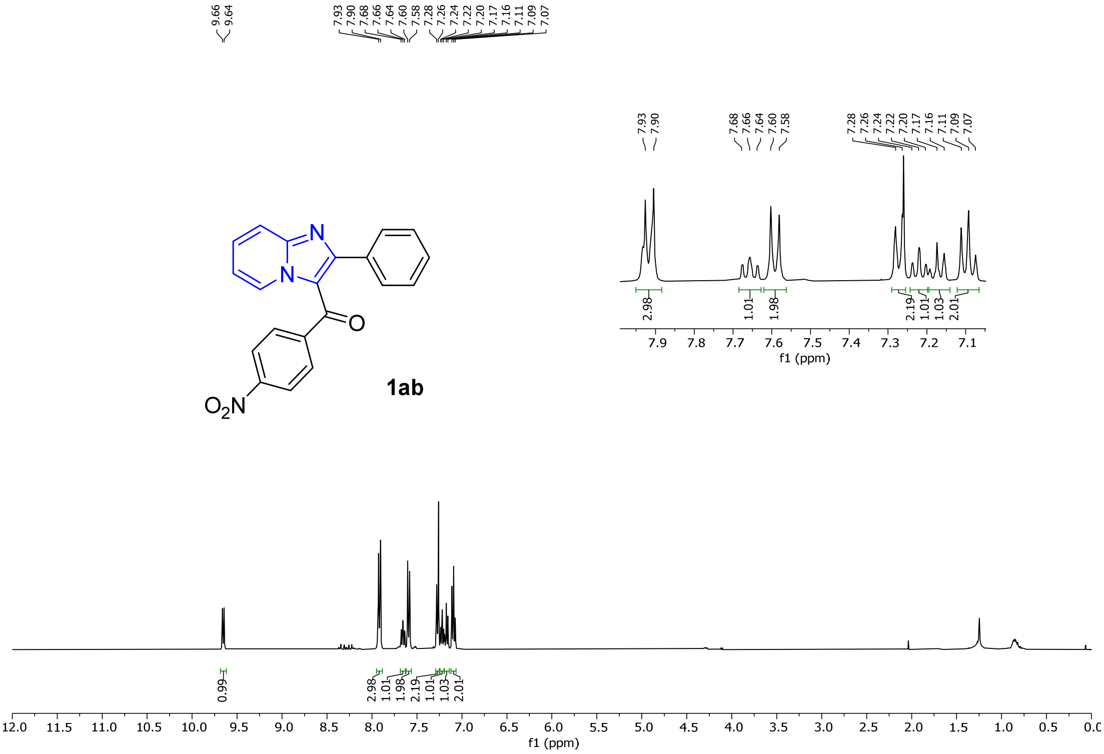

9.66
9.64
7.93
7.90
7.68
7.66
7.64
7.60
7.58
7.28
7.26
7.24
7.22
7.20
7.17
7.16
7.11
7.09
7.07
f1 (ppm)

1ab

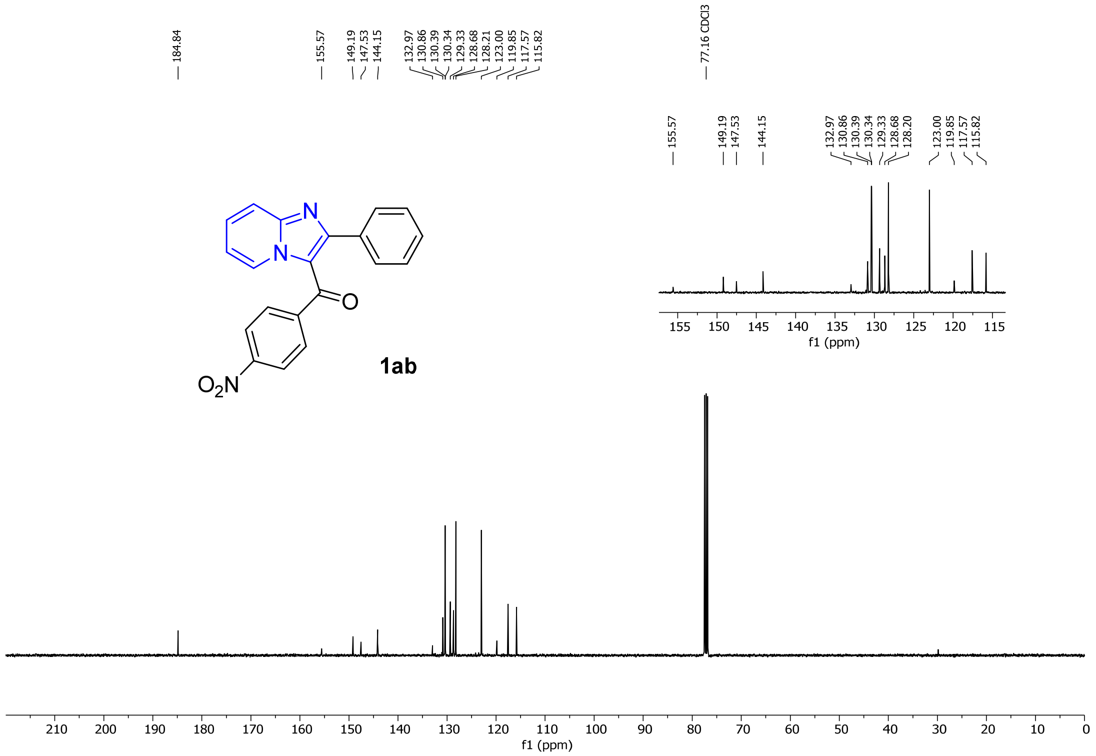

184.84
155.57
149.19
147.53
144.15
132.97
130.86
130.39
130.34
129.33
128.68
128.21
123.00
119.85
117.57
115.82
77.16 CDCl3
f1 (ppm)

1ab

^{1}H NMR (400 MHz, $CDCl_3$), ^{13}C NMR (101 MHz, $CDCl_3$) (4-nitrophenyl)(2-phenylimidazo[1,2-*a*]pyridin-3-yl)methanone (**1ab**)

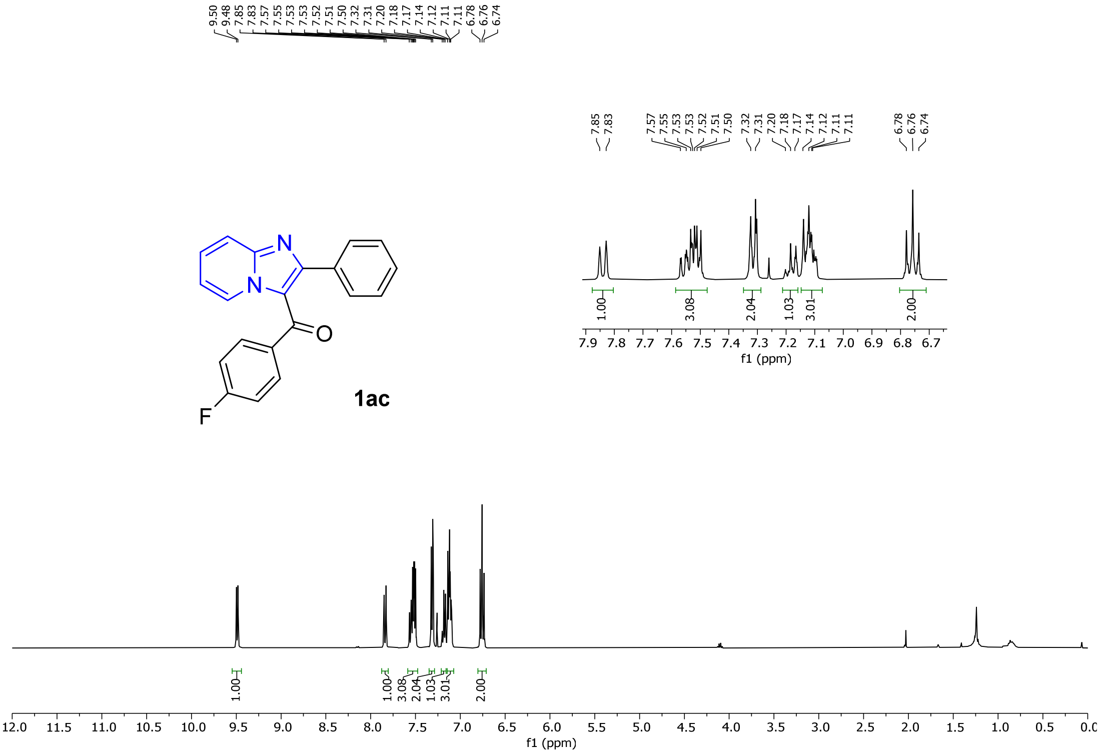

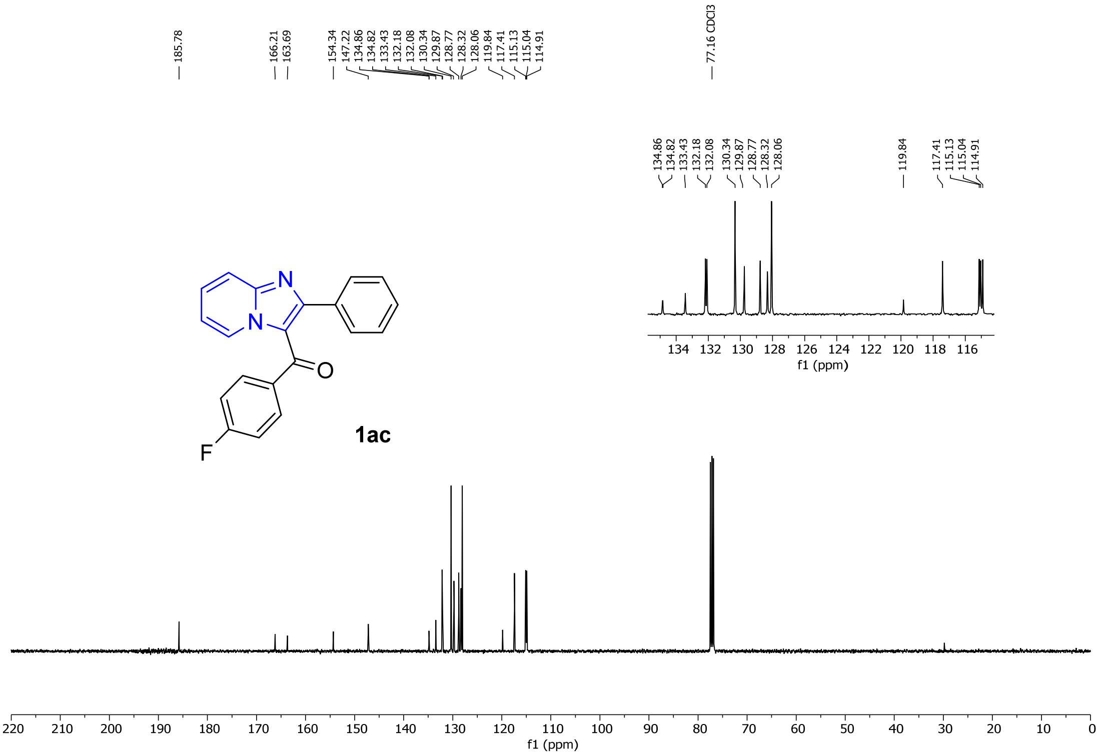

^{1}H NMR (400 MHz, $CDCl_3$), ^{13}C NMR (101 MHz, $CDCl_3$) (4-fluorophenyl)(2-phenylimidazo[1,2-*a*]pyridin-3-yl)methanone (**1ac**)

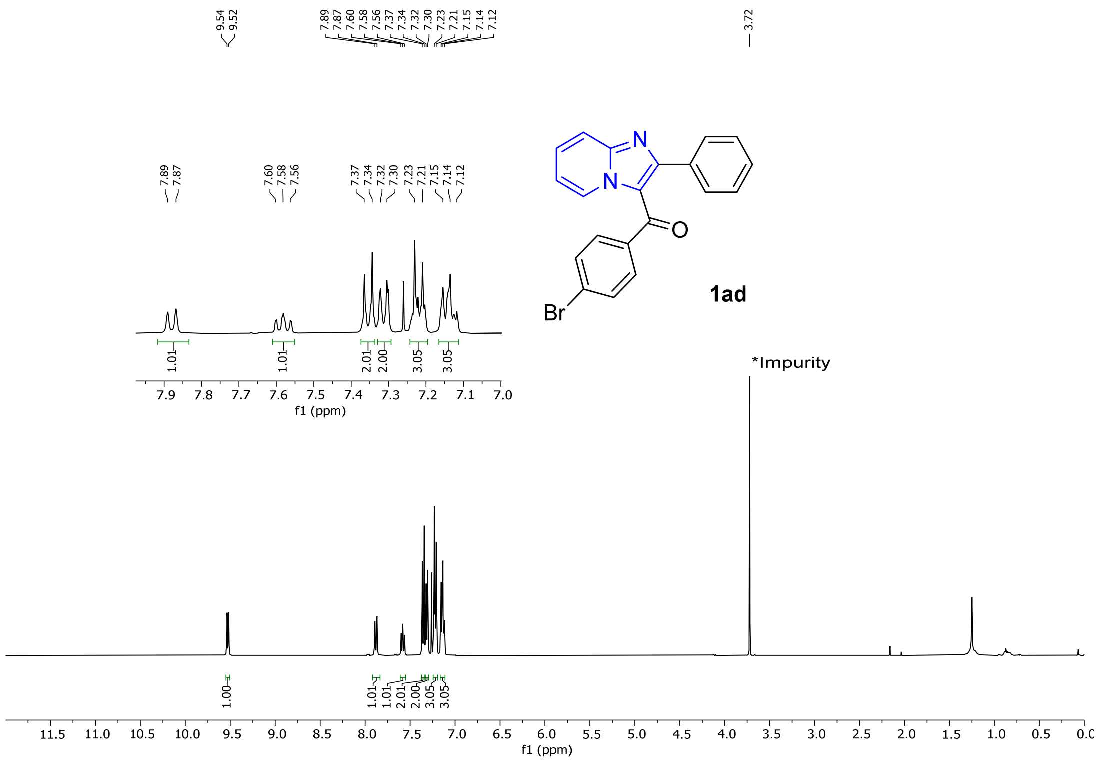

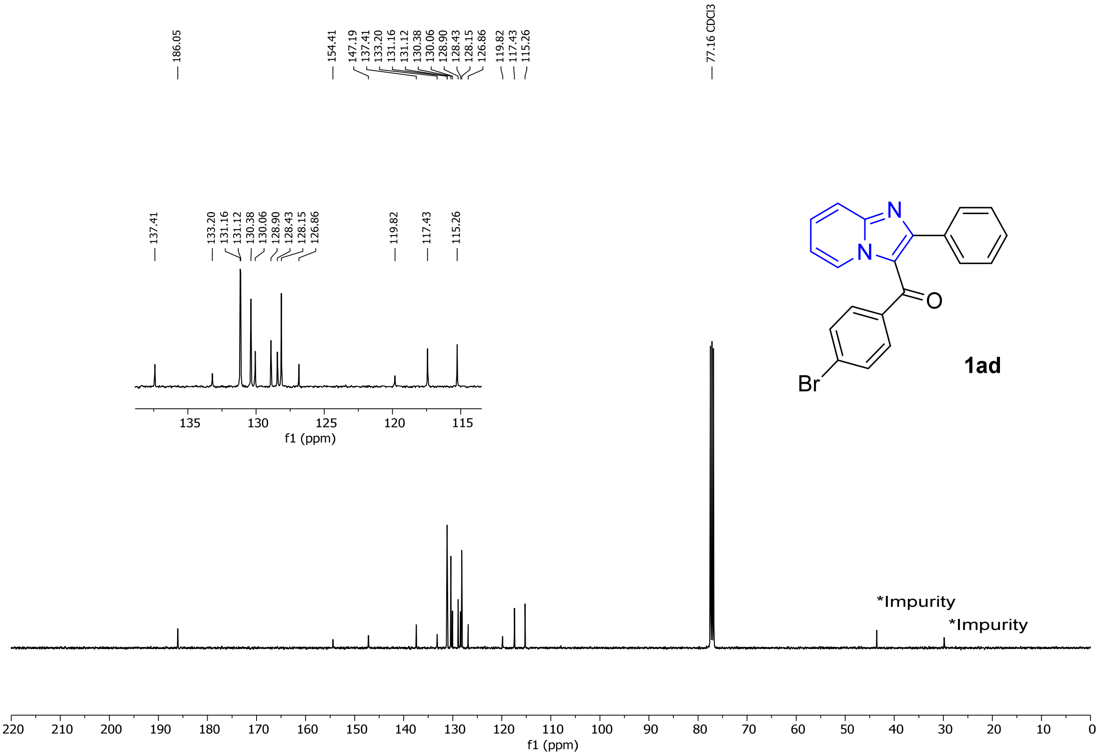

^{1}H NMR (400 MHz, $CDCl_3$), ^{13}C NMR (101 MHz, $CDCl_3$) (4-bromophenyl)(2-phenylimidazo[1,2-*a*]pyridin-3-yl)methanone (**1ad**)

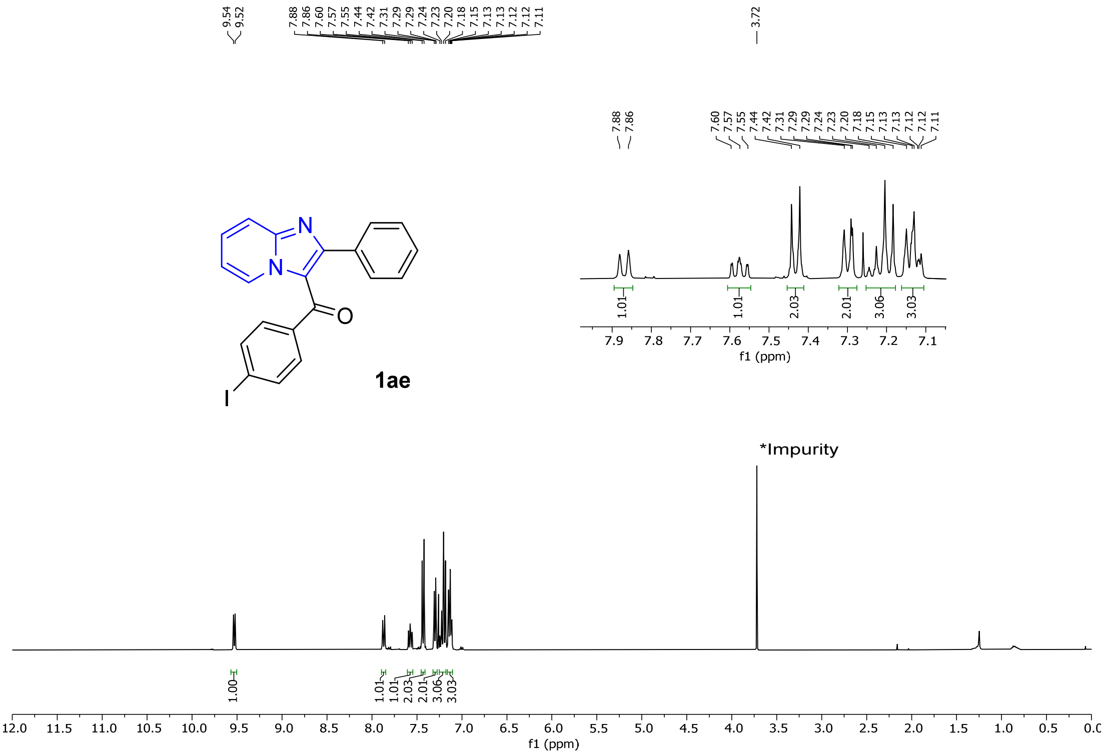

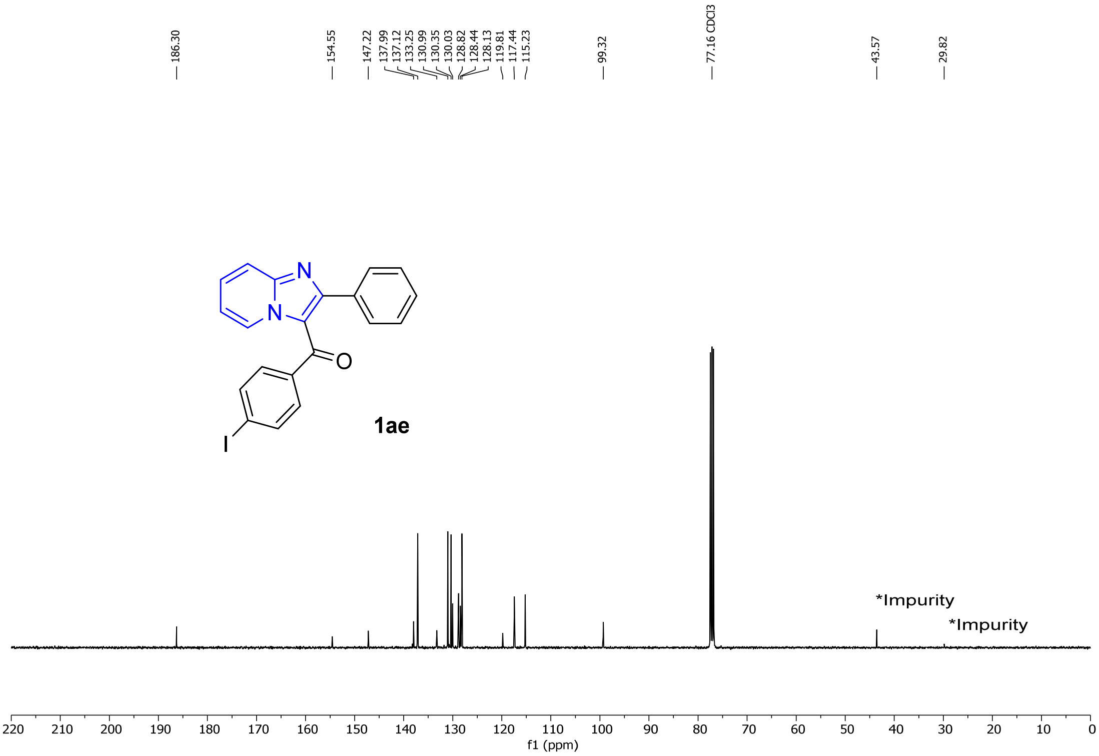

^{1}H NMR (400 MHz, CDCl$_3$), ^{13}C NMR (101 MHz, CDCl$_3$) (4-iodophenyl)(2-phenylimidazo[1,2-*a*]pyridin-3-yl)methanone (**1ae**)

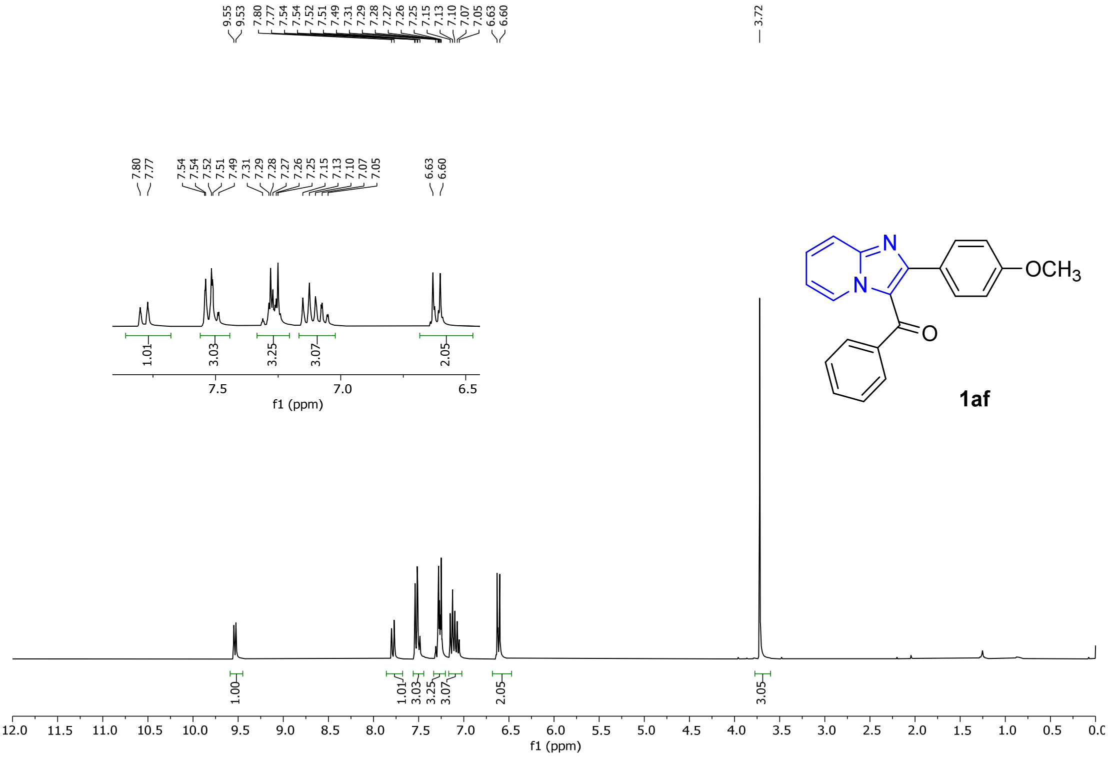

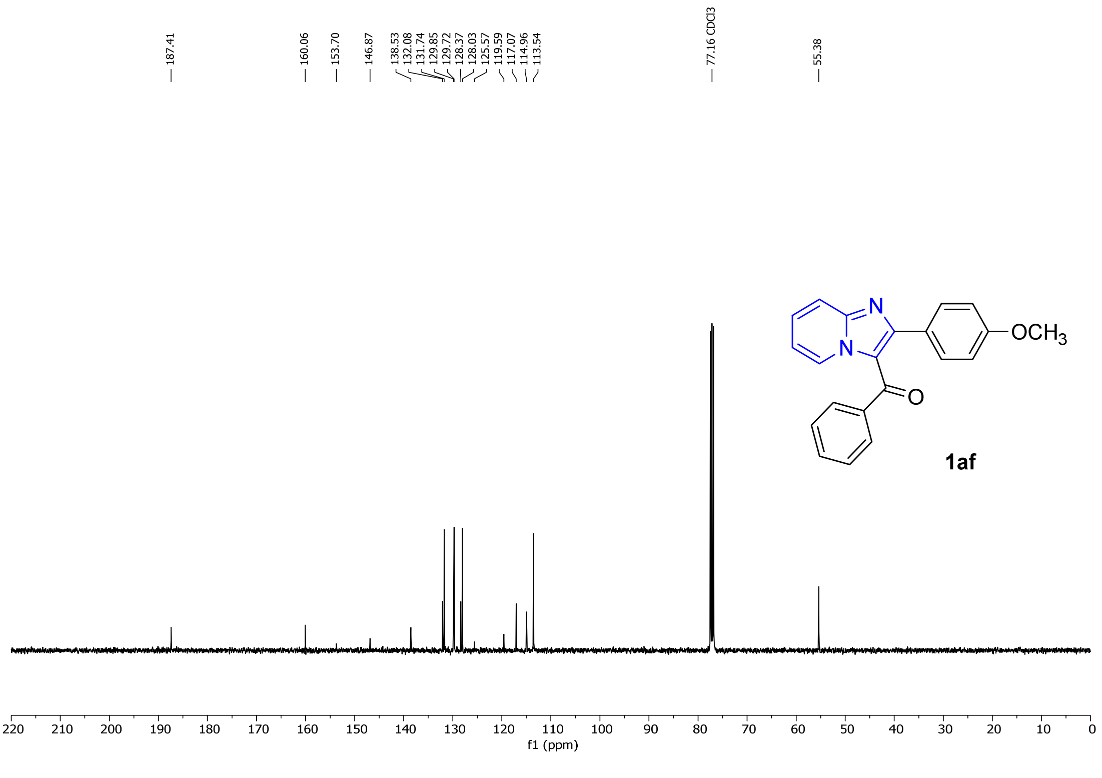

^{1}H NMR (400 MHz, $CDCl_3$), ^{13}C NMR (101 MHz, $CDCl_3$) (2-(4-methoxyphenyl)imidazo[1,2-*a*]pyridin-3-yl)(phenyl)methanone (**1af**)

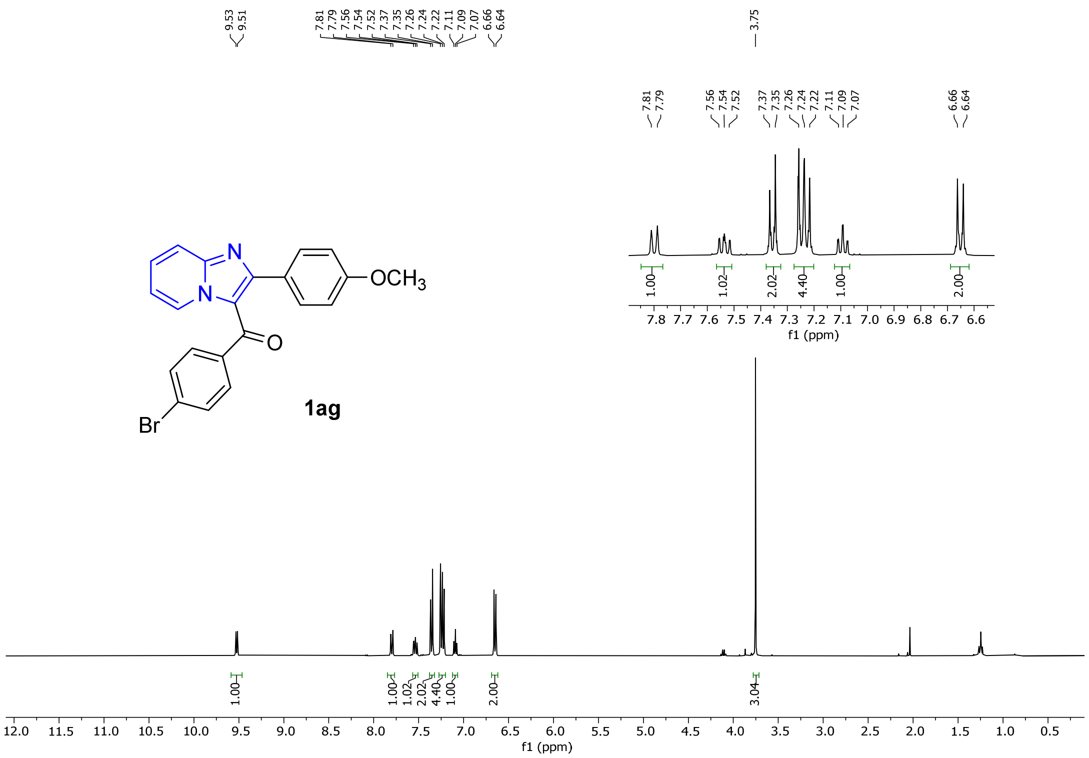

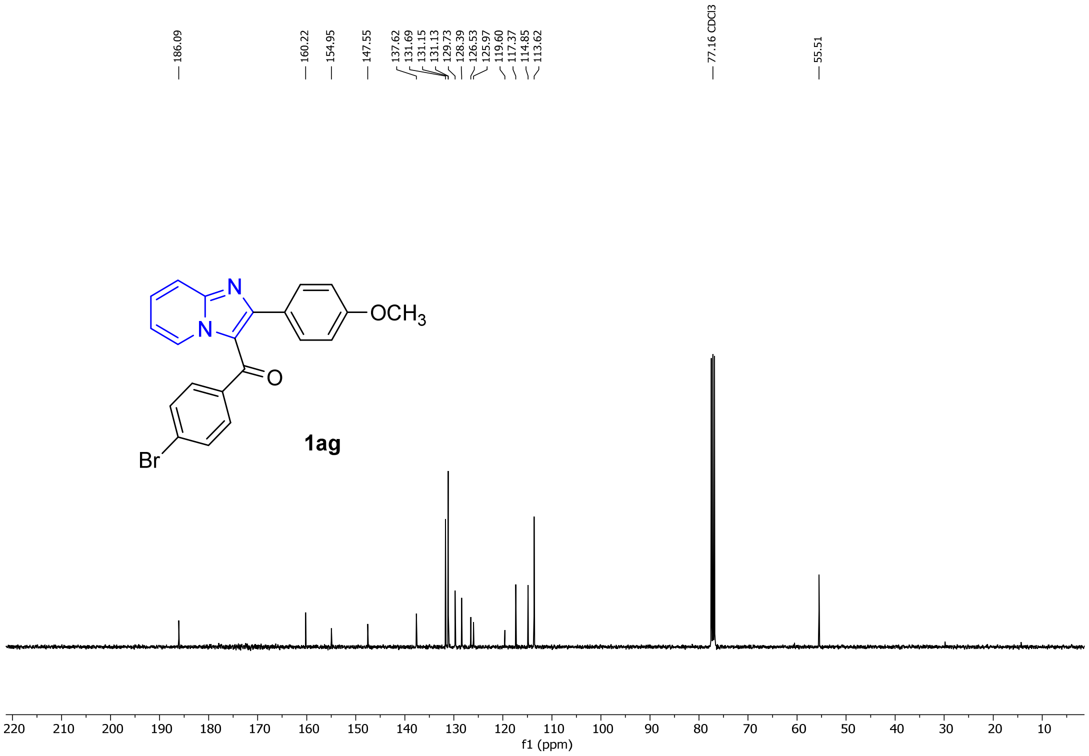

^{1}H NMR (400 MHz, $CDCl_3$), ^{13}C NMR (101 MHz, $CDCl_3$) (4-bromophenyl)(2-(4-methoxyphenyl)imidazo[1,2-*a*]pyridin-3-yl)methanone (**1ag**)

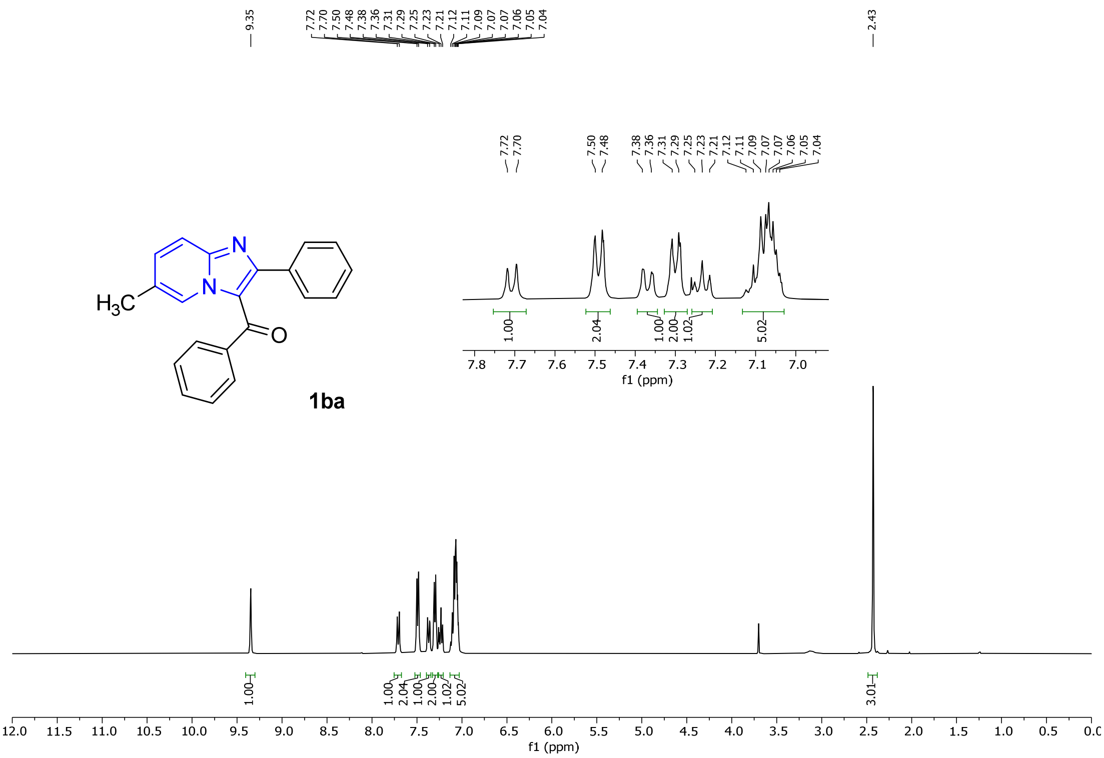

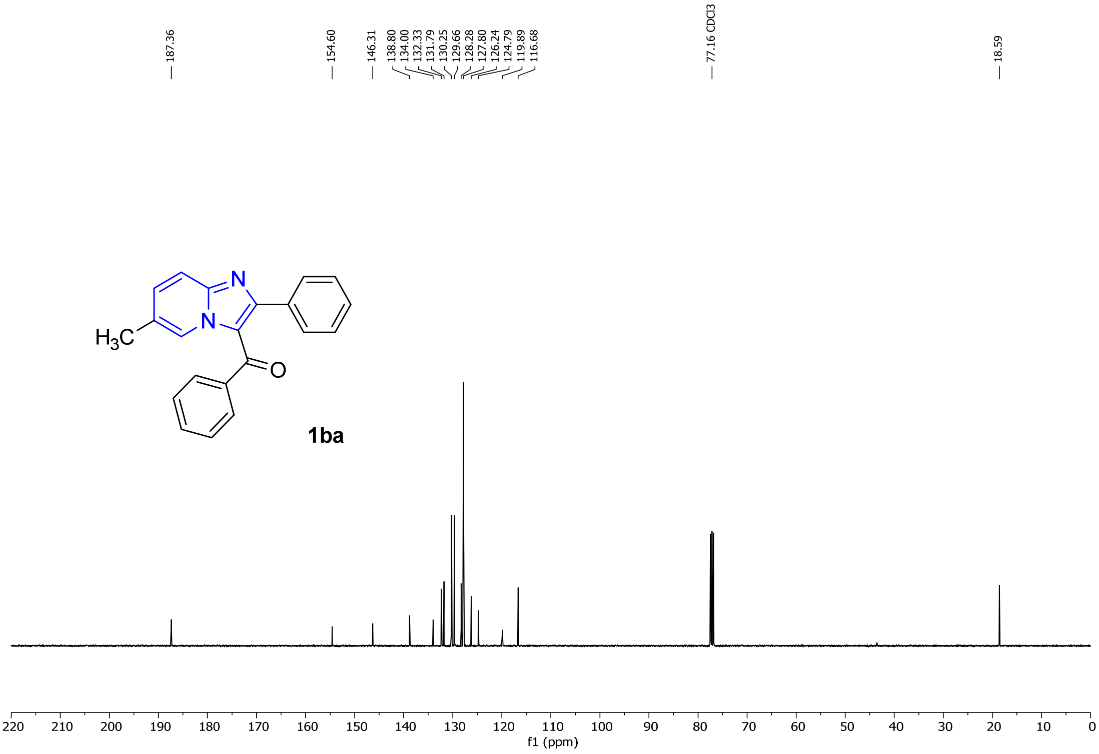

^{1}H NMR (400 MHz, $CDCl_3$), ^{13}C NMR (101 MHz, $CDCl_3$) (6-methyl-2-phenylimidazo[1,2-*a*]pyridin-3-yl)(phenyl)methanone (**1ba**)

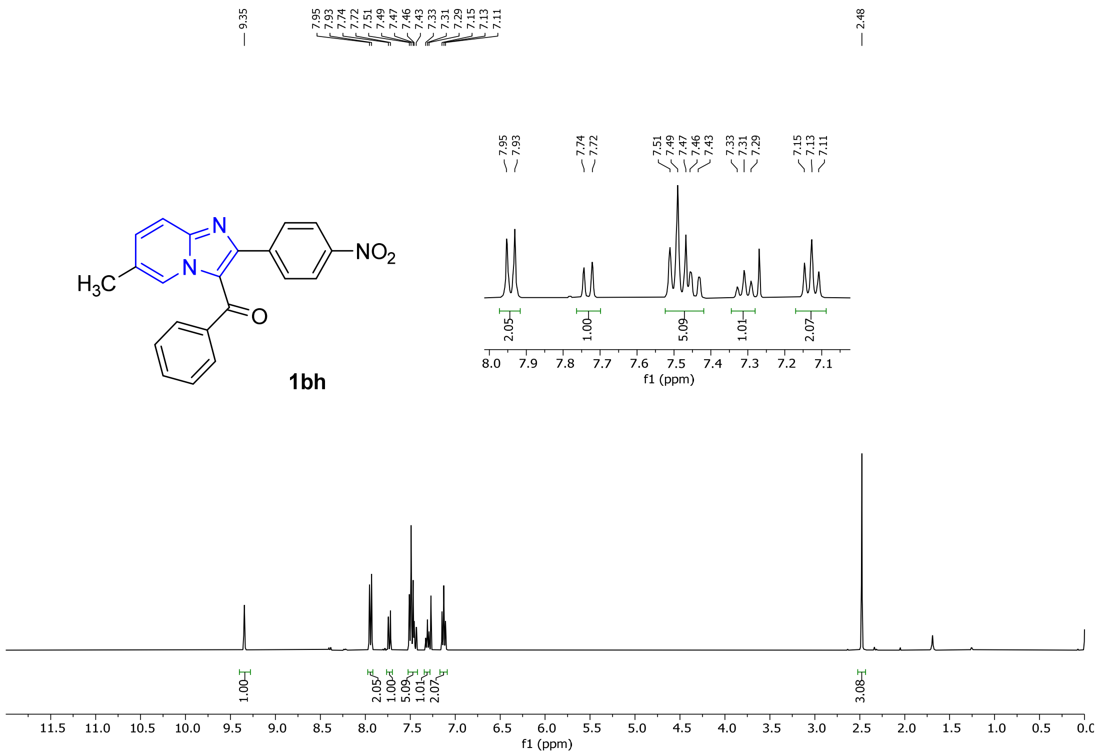

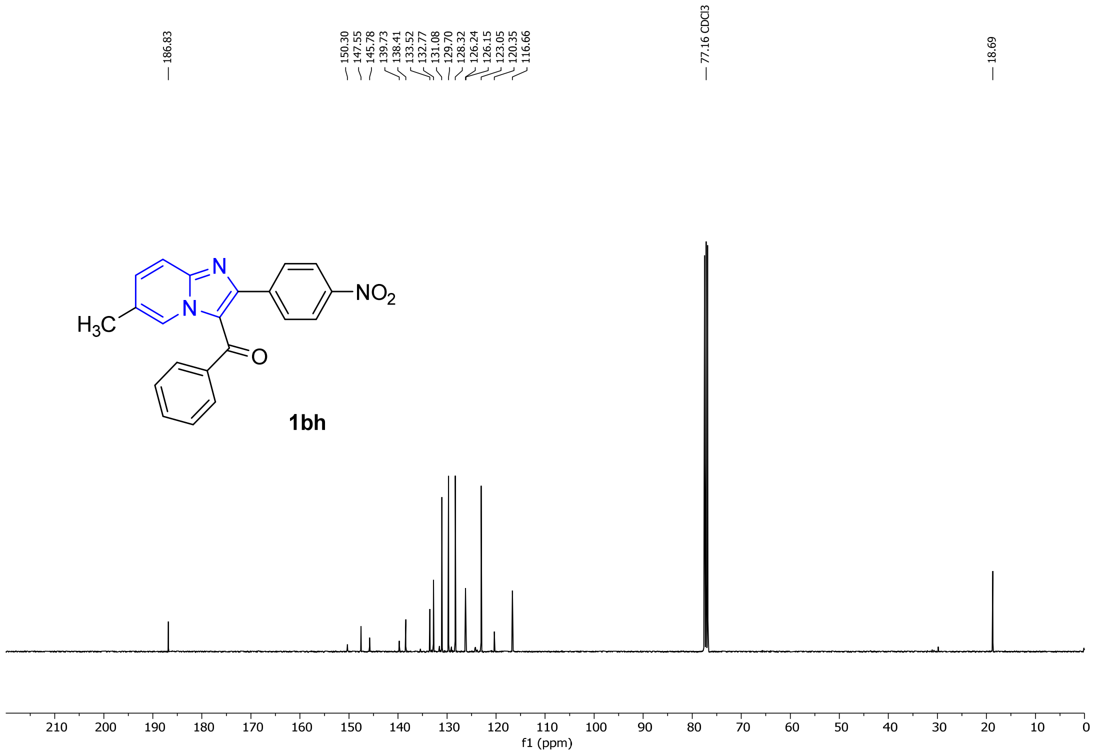

^{1}H NMR (400 MHz, $CDCl_3$), ^{13}C NMR (101 MHz, $CDCl_3$) (6-methyl-2-(4-nitrophenyl)imidazo[1,2-*a*]pyridin-3-yl)(phenyl)methanone (**1bh**)

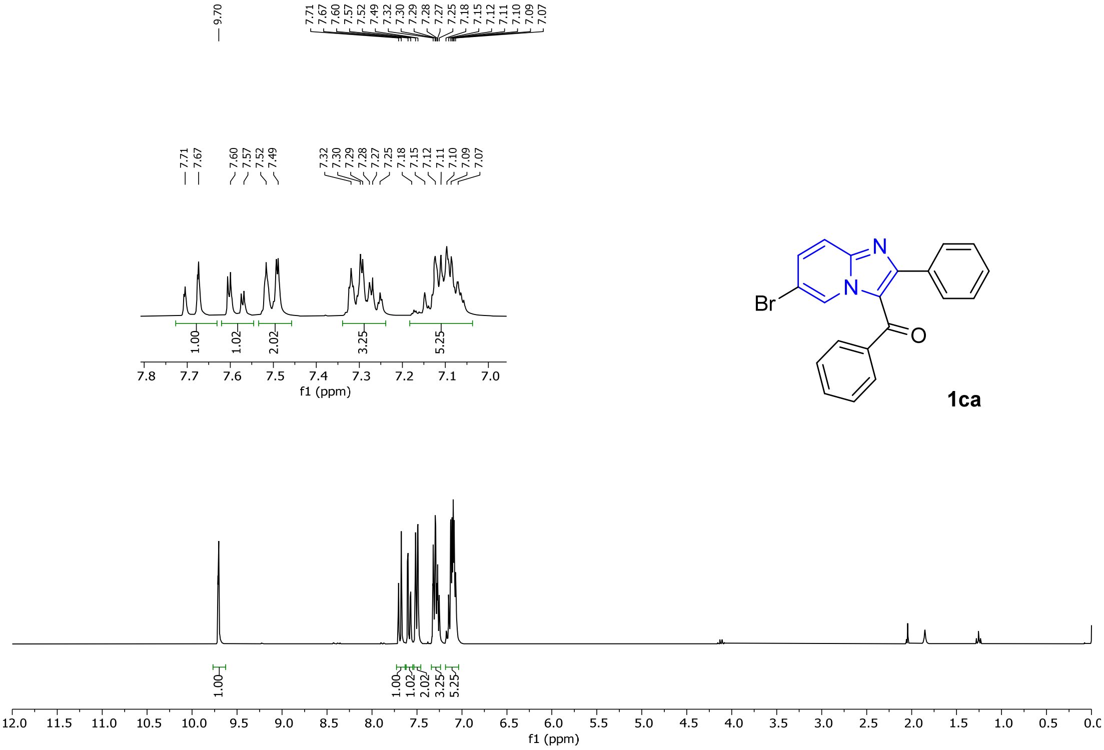

1ca

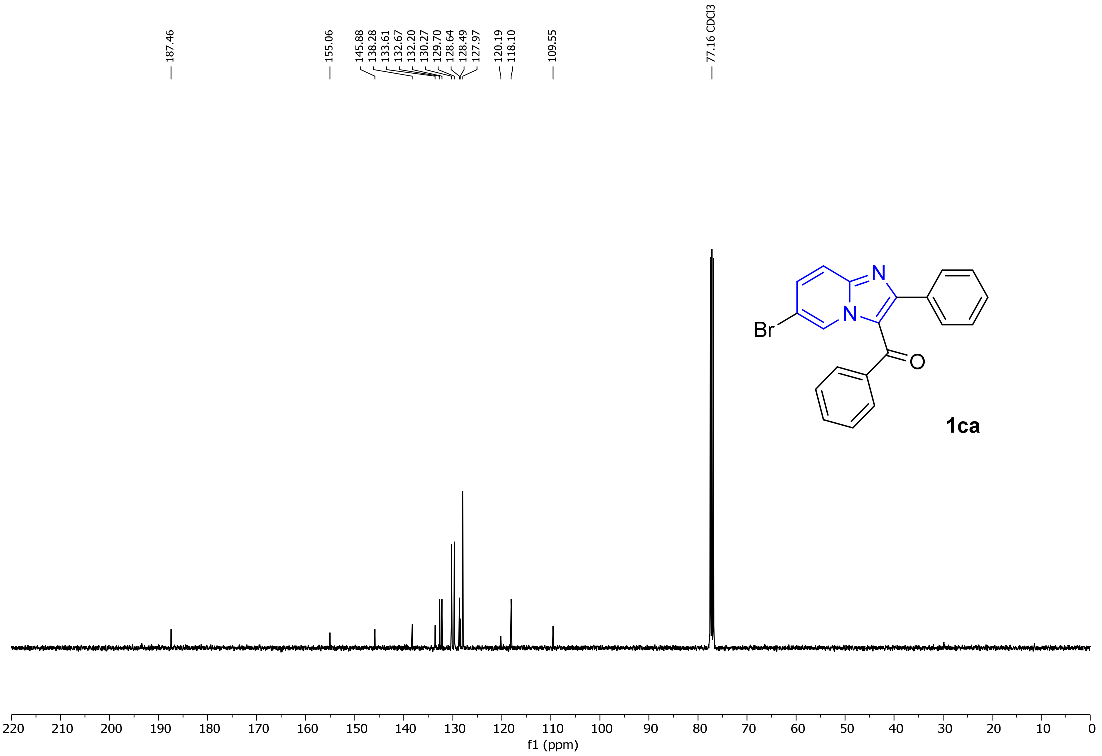

1ca

^{1}H NMR (400 MHz, $CDCl_3$), ^{13}C NMR (101 MHz, $CDCl_3$) (6-bromo-2-phenylimidazo[1,2-*a*]pyridin-3-yl)(phenyl)methanone (**1ca**)

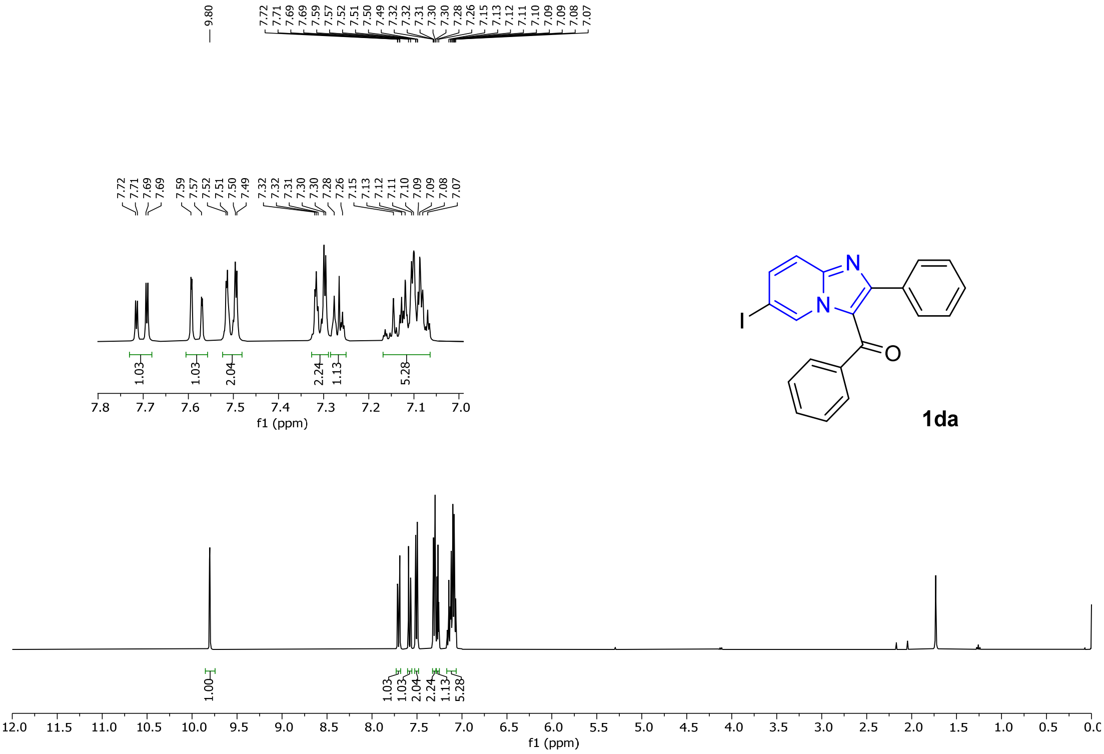

1da

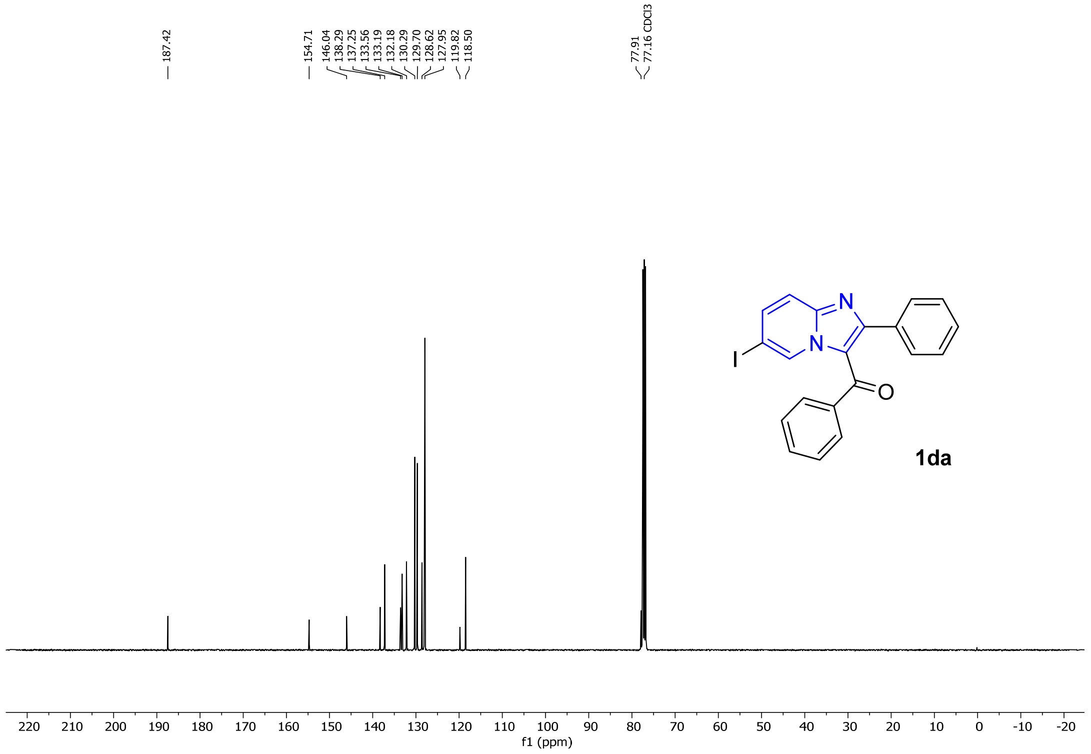

1da

^{1}H NMR (400 MHz, $CDCl_3$), ^{13}C NMR (101 MHz, $CDCl_3$) (6-iodo-2-phenylimidazo[1,2-*a*]pyridin-3-yl)(phenyl)methanone (**1da**)

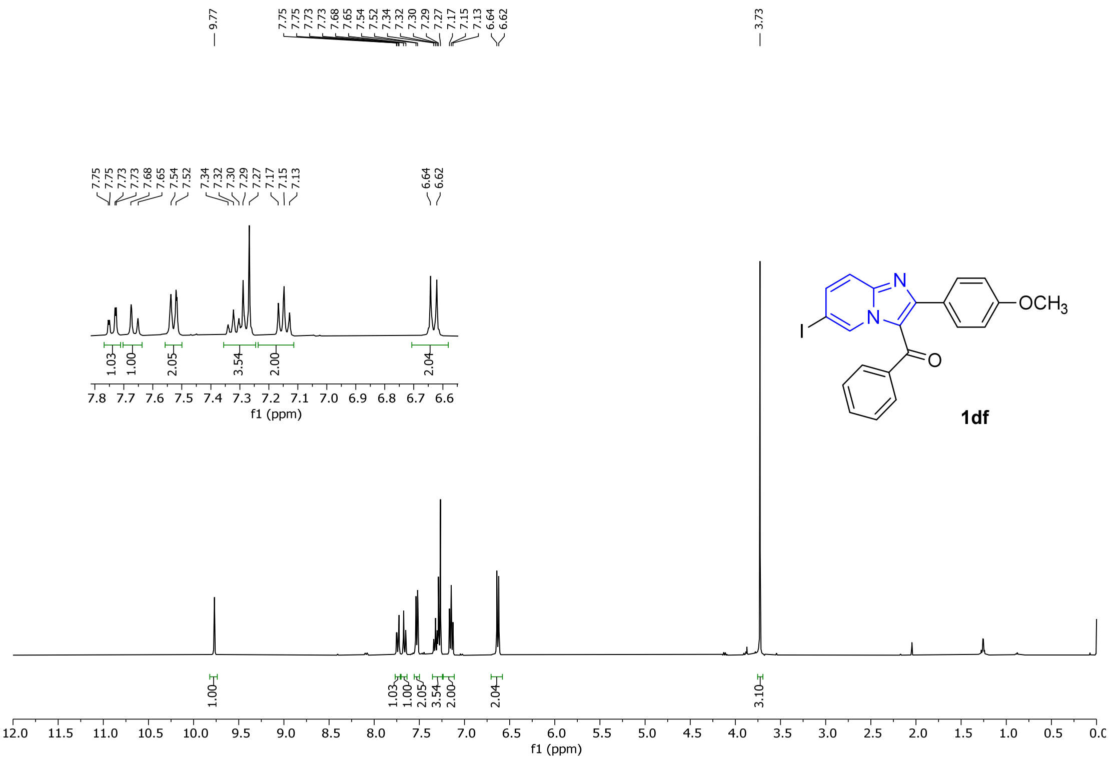

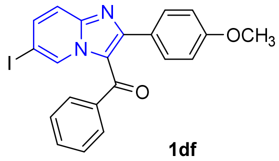

1df

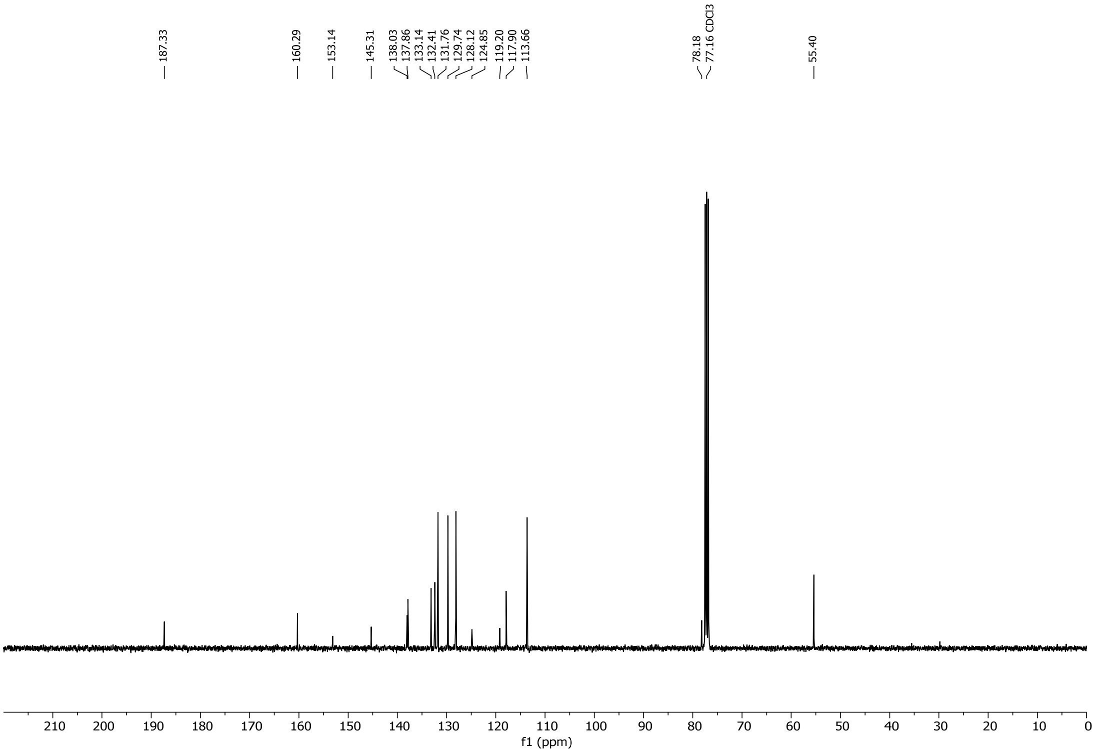

^{1}H NMR (400 MHz, $CDCl_3$), ^{13}C NMR (101 MHz, $CDCl_3$) (6-iodo-2-(4-methoxyphenyl)imidazo[1,2-*a*]pyridin-3-yl)(phenyl)methanone (**1df**)

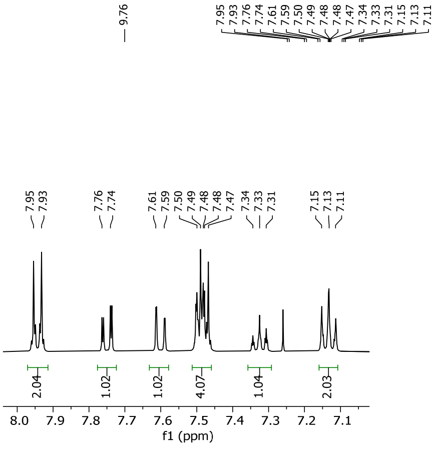
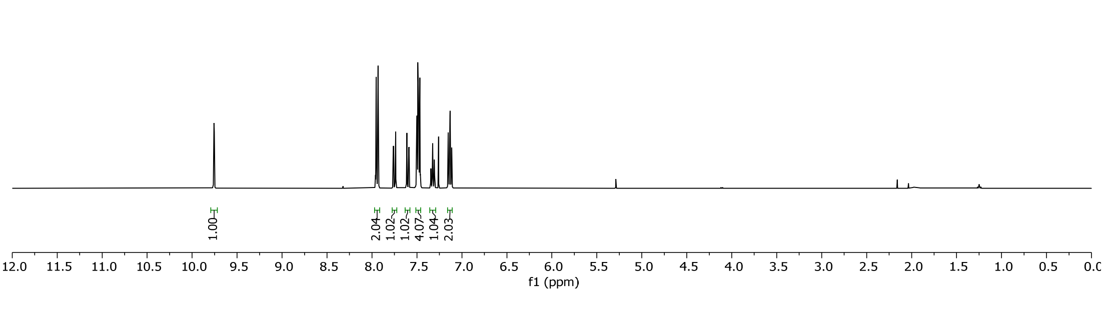

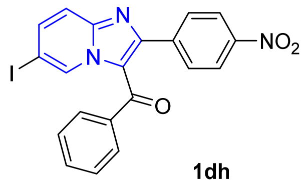

1dh

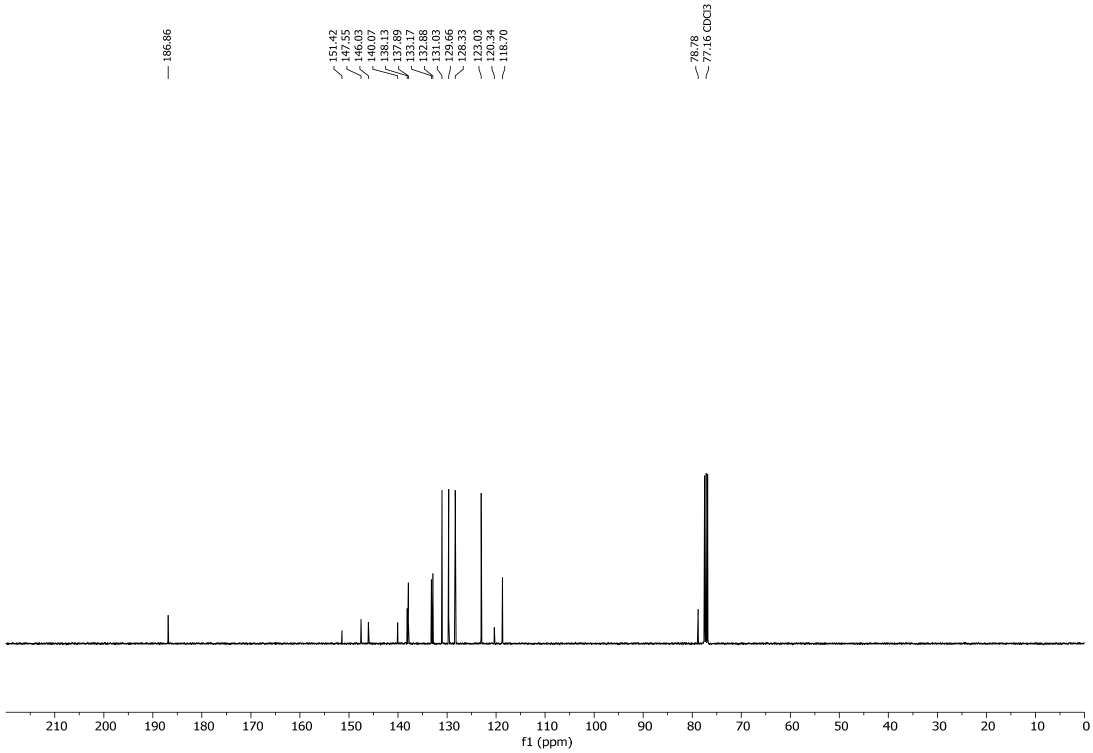

^{1}H NMR (400 MHz, $CDCl_3$), ^{13}C NMR (101 MHz, $CDCl_3$) (6-iodo-2-(4-nitrophenyl)imidazo[1,2-*a*]pyridin-3-yl)(phenyl)methanone (**1dh**)

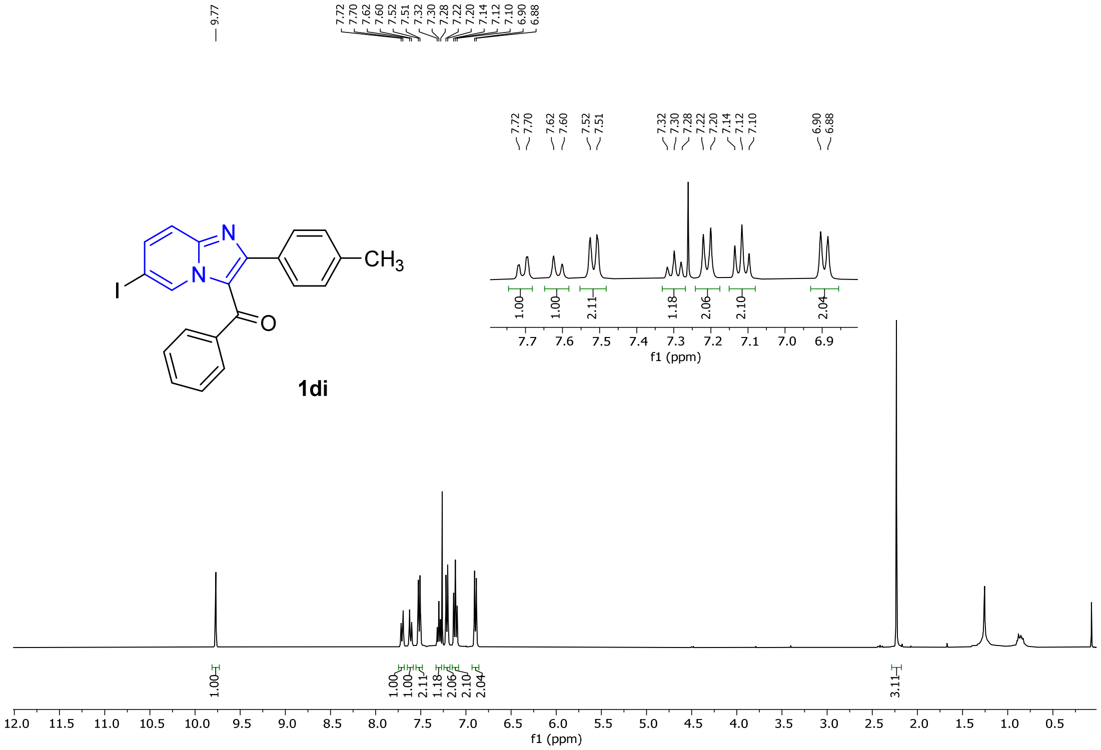

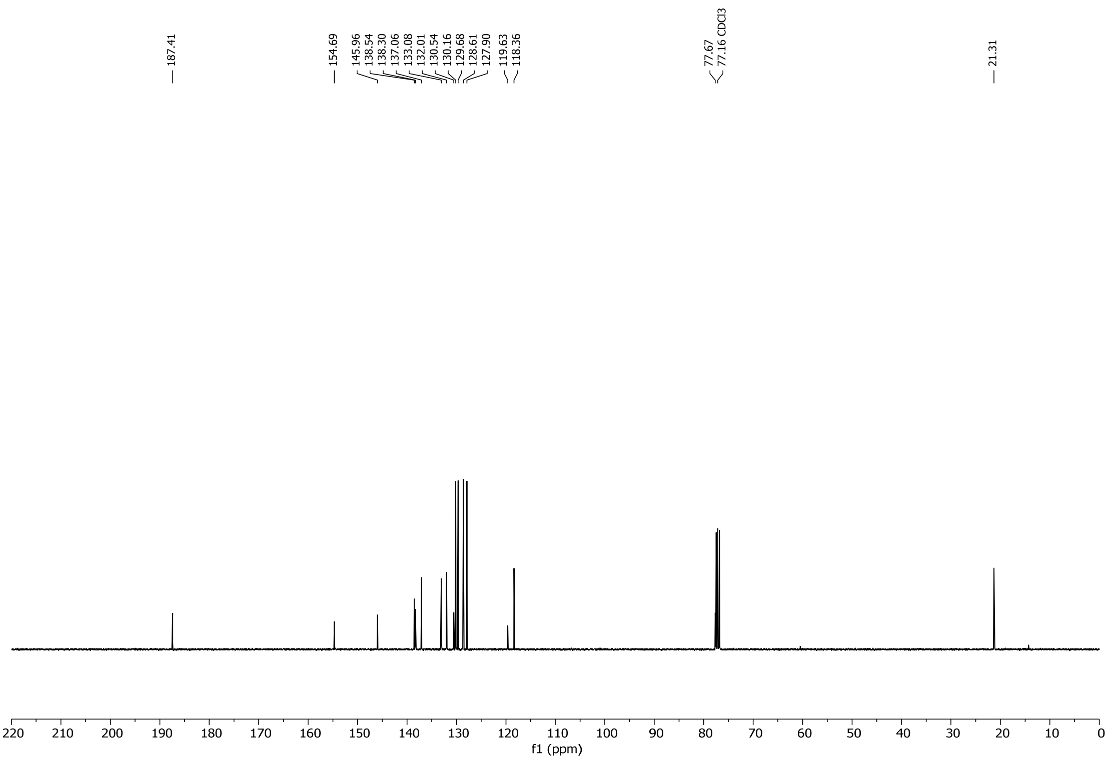

^{1}H NMR (400 MHz, $CDCl_3$), ^{13}C NMR (101 MHz, $CDCl_3$) (6-iodo-2-(p-tolyl)imidazo[1,2-*a*]pyridin-3-yl)(phenyl)methanone (**1di**)

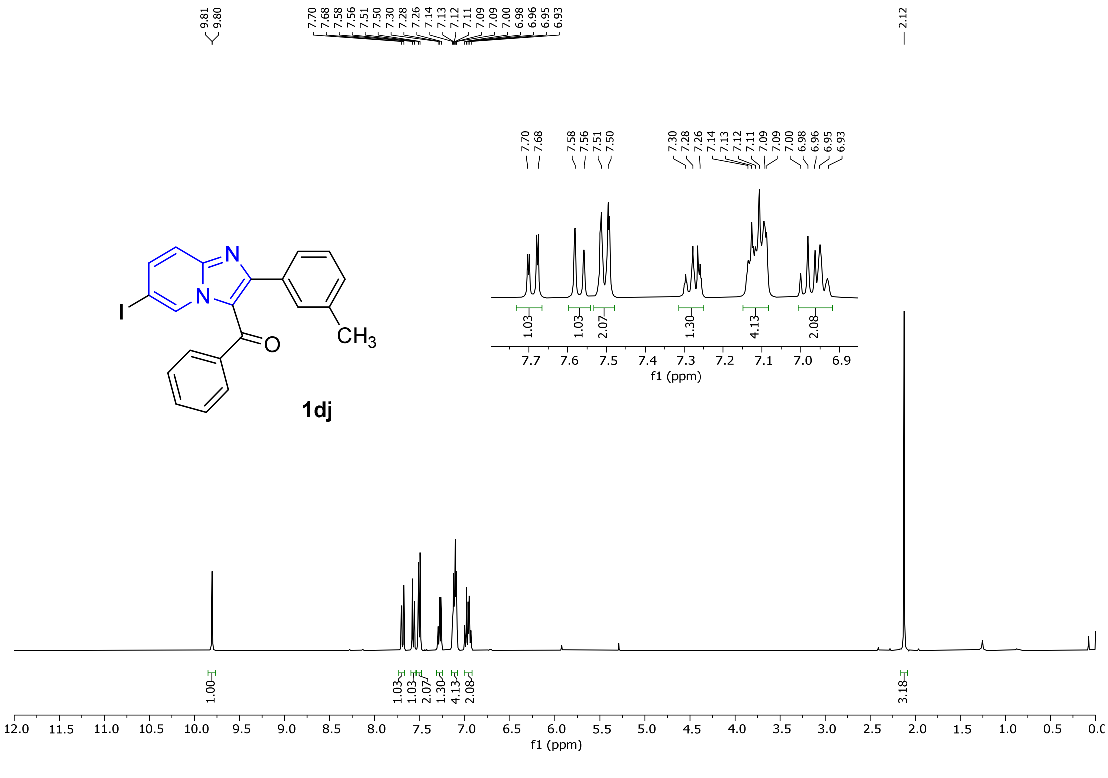

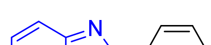

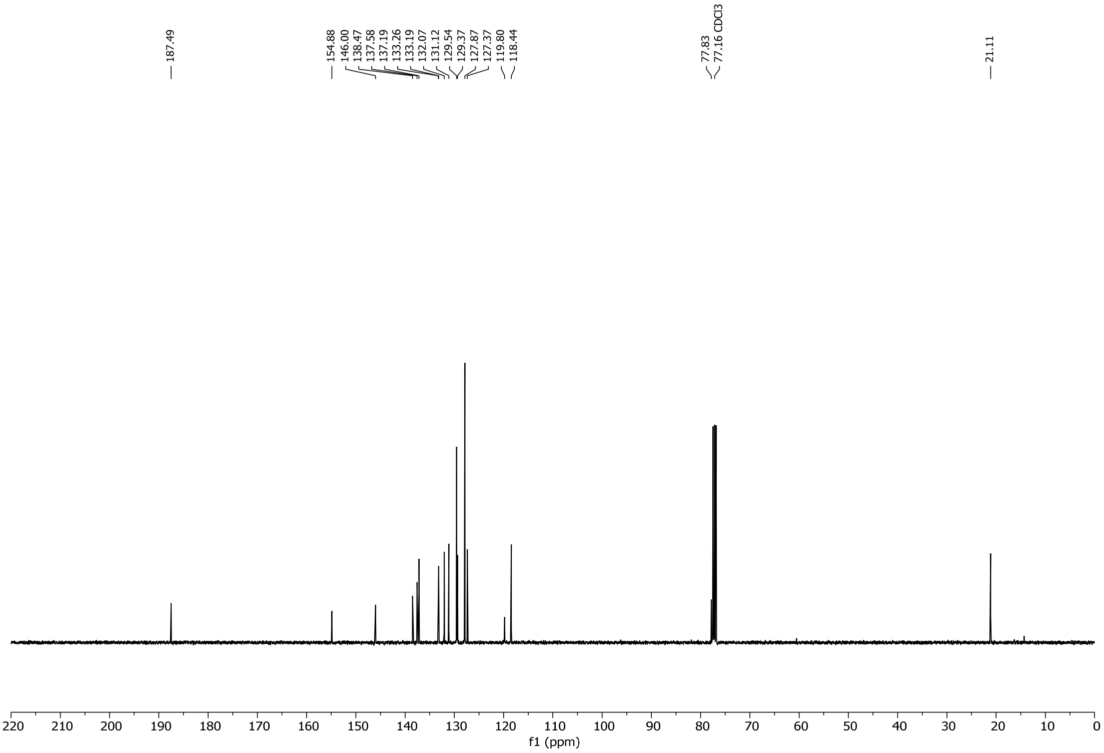

^1H NMR (400 MHz, $CDCl_3$), ^{13}C NMR (101 MHz, $CDCl_3$) (6-iodo-2-(m-tolyl)imidazo[1,2-*a*]pyridin-3-yl)(phenyl)methanone (**1dj**)

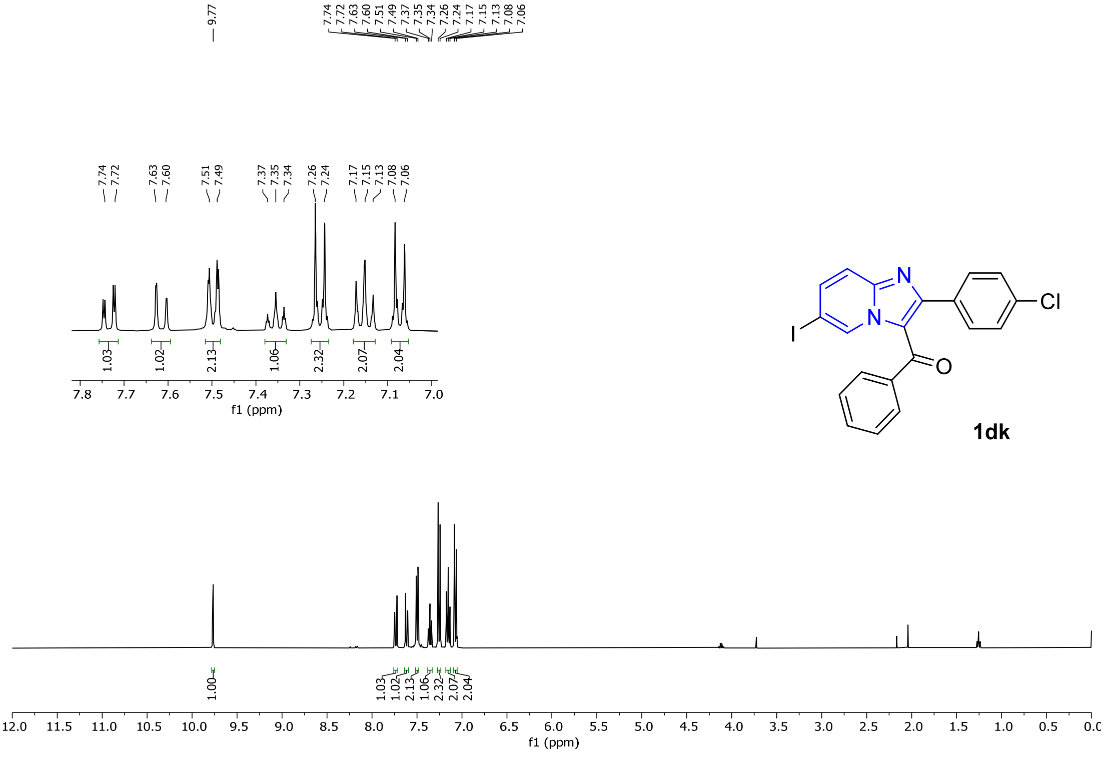

1dk

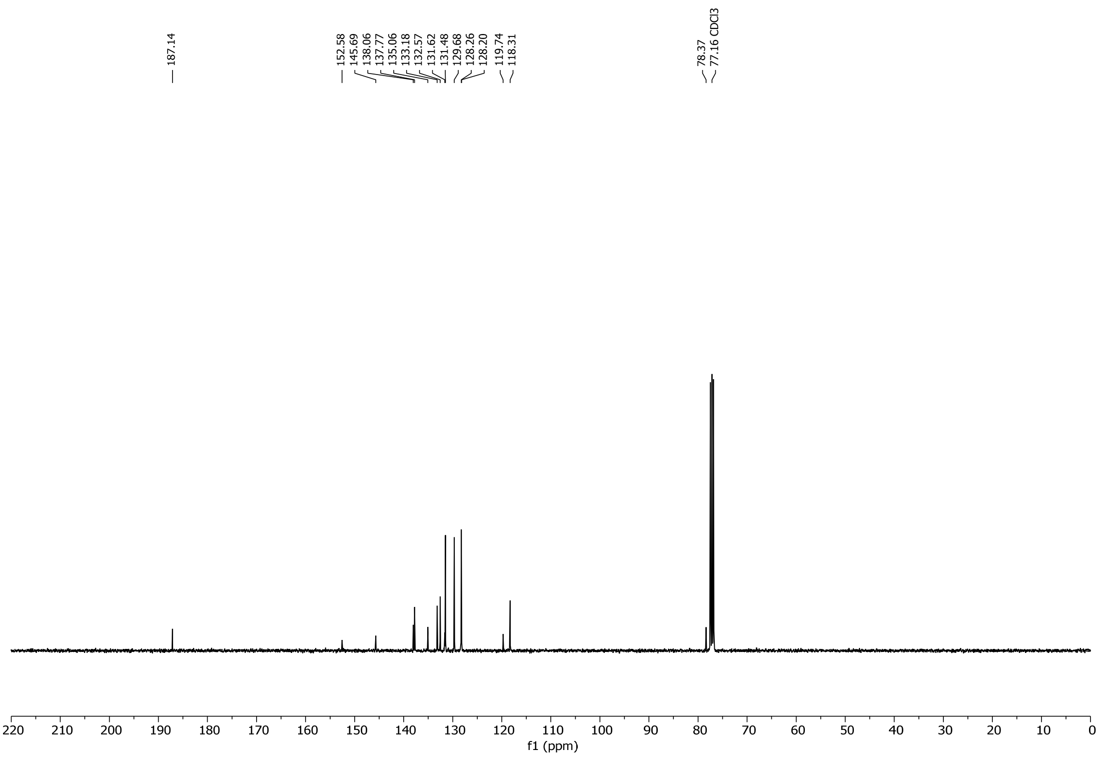

^{1}H NMR (400 MHz, $CDCl_3$), ^{13}C NMR (101 MHz, $CDCl_3$) (2-(4-chlorophenyl)-6-iodoimidazo[1,2-*a*]pyridin-3-yl)(phenyl)methanone (**1dk**)

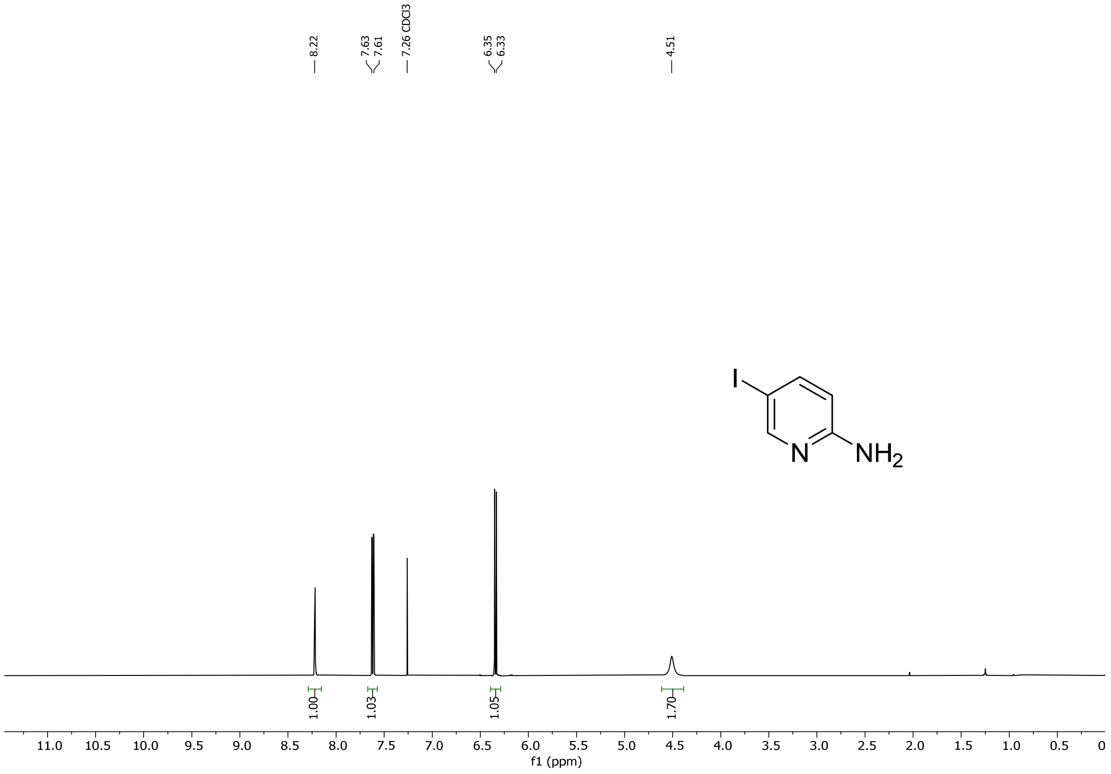

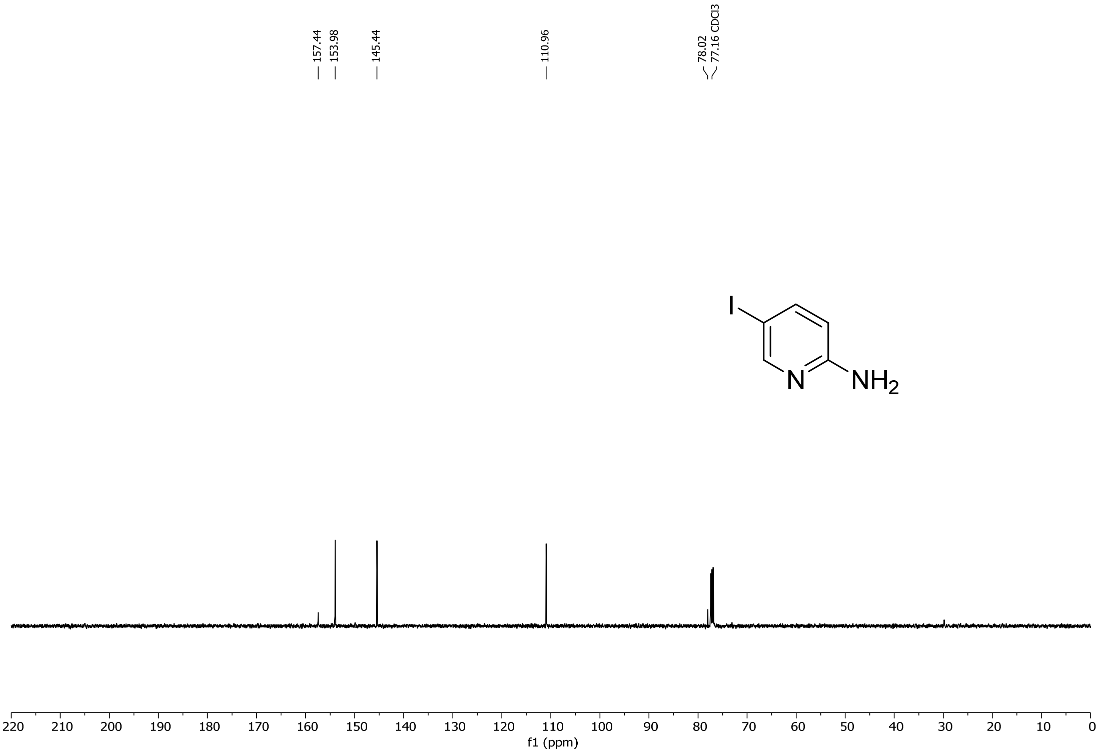

^{1}H NMR (400 MHz, $CDCl_3$), ^{13}C NMR (101 MHz, $CDCl_3$) 5-iodopyridin-2-amine (**2d**)

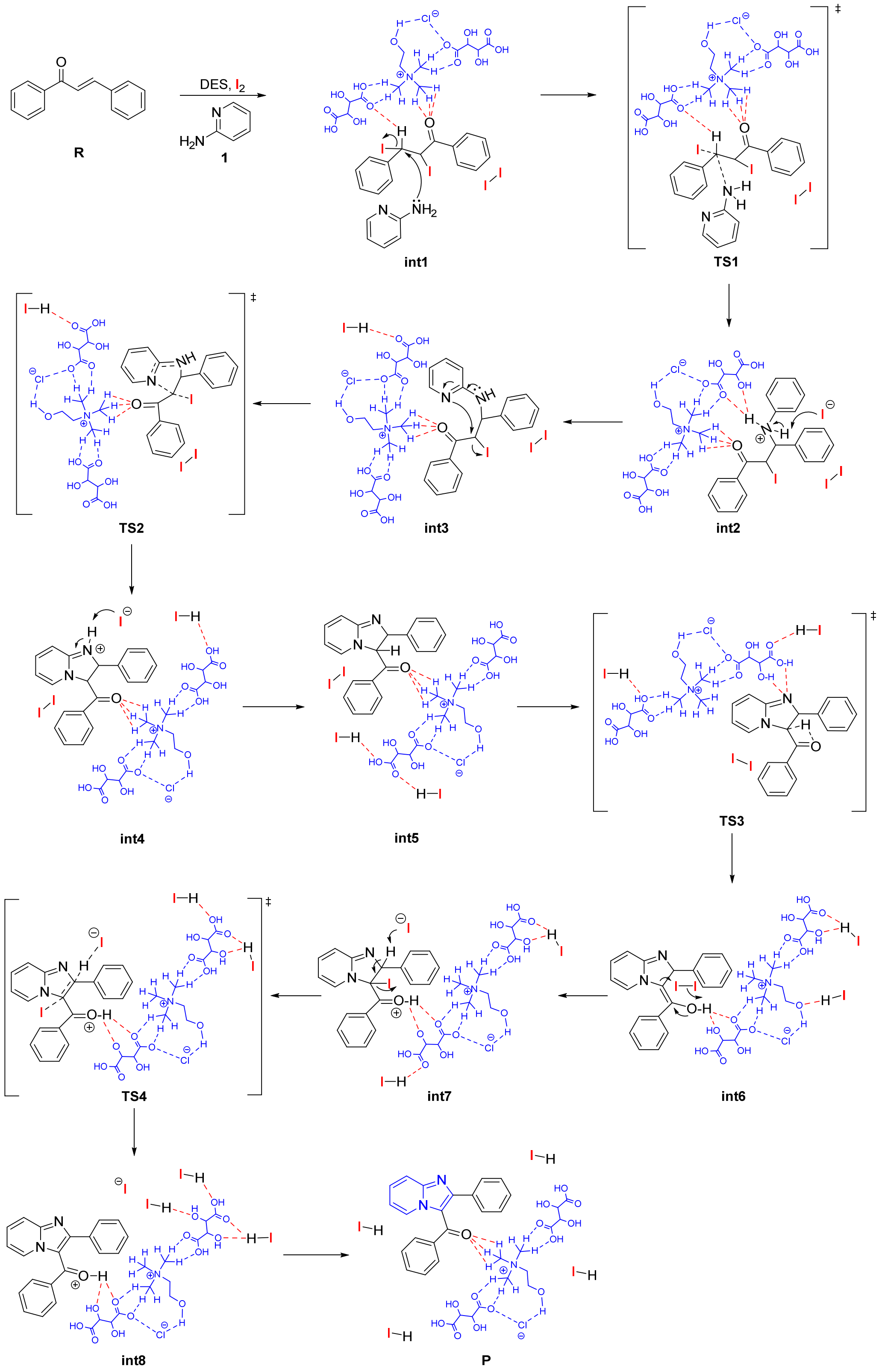

Scheme S1. Mechanistic proposal for the synthesis of imidazo[1,2-*a*]pyridines **1aa** in DES

Gas Phase Energies and Cartesian Coordinates

2-aminopiridina (1)

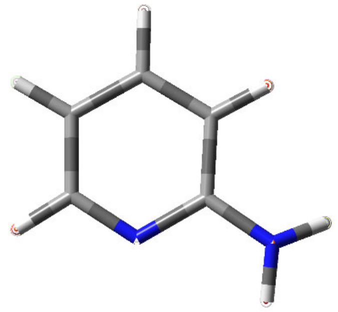

	**(Hartree/Particle)**
Zero-point correction=	0.096970
Thermal correction to Energy=	0.102840
Thermal correction to Enthalpy=	0.103784
Thermal correction to Gibbs Free Energy=	0.067669
Sum of electronic and zero-point Energies=	0.145279
Sum of electronic and thermal Energies=	0.151149
Sum of electronic and thermal Enthalpies=	0.152093
Sum of electronic and thermal Free Energies=	0.115978

Charge: 0 Multiplicity:1

C	1.85262300	-0.05322400	-0.00991600
C	1.19457600	1.18287200	-0.00327400
C	-0.19615900	1.23386800	0.01198100
C	-0.90826500	0.00696000	0.01052700
C	1.07947200	-1.22406300	0.00262200
H	2.93562700	-0.10711900	-0.02348200
H	1.77271200	2.10767000	-0.00987700
H	-0.72222100	2.18157300	0.00453600
H	1.54704600	-2.21664700	0.00378600
N	-0.27584700	-1.21776500	0.02426900
N	-2.30877000	-0.02296600	-0.08292200
H	-2.75700400	-0.91890300	0.06915100
H	-2.81732300	0.76006800	0.29481400

Chalcona (R)

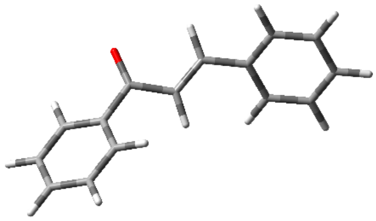

	(Hartree/Particle)
Zero-point correction=	0.210711
Thermal correction to Energy=	0.224541
Thermal correction to Enthalpy=	0.225485
Thermal correction to Gibbs Free Energy=	0.166694
Sum of electronic and zero-point Energies=	0.260619
Sum of electronic and thermal Energies=	0.274449
Sum of electronic and thermal Enthalpies=	0.275393
Sum of electronic and thermal Free Energies=	0.216603

Charge: 0 Multiplicity:1

C	-4.82166900	-1.21853600	0.15830800
C	-3.67747800	-1.67043300	0.82530400
C	-2.49132200	-0.93465400	0.75881200
C	-2.45062800	0.25917200	0.02569300
C	-3.60279200	0.71952200	-0.62874500
C	-4.78453200	-0.02384100	-0.56792900
H	-5.74378300	-1.79655400	0.20816500
H	-3.71130600	-2.59568100	1.39795100
H	-1.60459400	-1.28599000	1.28668100
H	-3.56878400	1.66385600	-1.17695500
H	-5.67679700	0.33133300	-1.08106300
C	-1.20366100	1.08528800	-0.05401100
C	0.08207800	0.34570300	-0.13525700
C	1.25183900	0.97357700	0.05407500
C	2.56480100	0.31360700	-0.01285100
C	3.57532200	0.70351200	0.88436300
C	2.82992600	-0.68720600	-0.96204800
C	4.82783500	0.08840600	0.83808300
H	3.37970500	1.48213400	1.62078500

C	4.08856700	-1.29275600	-1.00877900
H	2.05688800	-0.98449100	-1.66972700
C	5.08701300	-0.90923400	-0.10839700
H	5.60584100	0.38835000	1.53884500
H	4.29082500	-2.06536100	-1.74930000
H	6.06602900	-1.38424500	-0.14464300
H	1.27319900	2.04921700	0.28567900
H	0.01768300	-0.71739200	-0.36347900
O	-1.25458700	2.30025900	-0.06908200

Intermediary 1 (Int1)

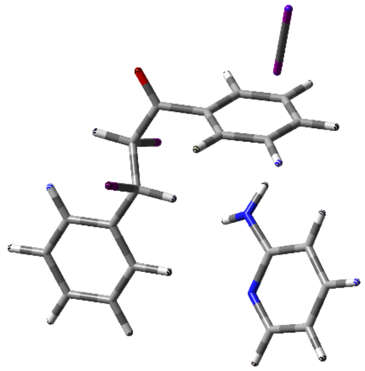

	(Hartree/Particle)
Zero-point correction=	0.212997
Thermal correction to Energy=	0.235016
Thermal correction to Enthalpy=	0.235961
Thermal correction to Gibbs Free Energy=	0.150455
Sum of electronic and zero-point Energies=	0.286324
Sum of electronic and thermal Energies=	0.308343
Sum of electronic and thermal Enthalpies=	0.309288
Sum of electronic and thermal Free Energies=	0.223782

Charge: 0 Multiplicity:1

C	6.63910000	0.00421100	-0.63563000
C	7.18769400	-0.04415900	0.65082500
C	6.35045400	-0.20609900	1.75834900
C	4.96814900	-0.31520200	1.58491500
C	4.41385100	-0.26121400	0.29518000
C	5.25953000	-0.10655900	-0.81639400
C	2.30557000	0.59691200	-0.81971900
C	2.93266300	-0.37180900	0.14265500
C	-0.81559500	-1.12004200	0.01852300
C	-0.03256200	0.03519600	0.15251600
C	0.79595900	0.45340000	-1.02168300
C	-1.61457100	-1.55889000	1.09352800
C	-1.60819800	-0.83322800	2.30493200
C	-0.84860800	0.33470300	2.41760700
C	-0.06556100	0.77112200	1.34516300
O	0.32549000	0.62562900	-2.11819200
H	7.29068500	0.12643600	-1.50045300

H	8.26456300	0.04223200	0.78784900
H	6.77502800	-0.24780100	2.76074100
H	4.32613800	-0.44280300	2.45524900
H	4.84473500	-0.08361100	-1.82429000
H	2.79782600	0.55989400	-1.82769800
H	-0.80167800	-1.68809900	-0.91888300
H	-2.07824200	-2.55491300	1.05221100
H	-2.18885900	-1.19560400	3.15792600
H	-0.86975600	0.91042700	3.34366000
H	0.51719700	1.69494800	1.44059300
H	2.41707200	-0.35838100	1.13356400
I	2.49857300	-2.40416000	-0.60008000
I	2.65883100	2.63558000	-0.16193100
I	-3.77356100	-0.43197200	0.35509300
I	-6.08395400	0.46399000	-0.42213000

Transition state 1 (TS1)

Imaginary frequency: -465.35 cm^{-1}

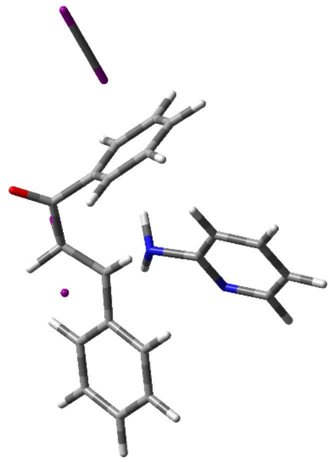

	**(Hartree/Particle)**
Zero-point correction=	0.311275
Thermal correction to Energy=	0.339946
Thermal correction to Enthalpy=	0.340891
Thermal correction to Gibbs Free Energy=	0.240998
Sum of electronic and zero-point Energies=	0.480009
Sum of electronic and thermal Energies=	0.508680
Sum of electronic and thermal Enthalpies=	0.509625
Sum of electronic and thermal Free Energies=	0.409732

Charge: 0 Multiplicity:1

C	6.15525900	-1.13283400	0.83633800
C	6.60261100	-0.01876700	1.54943800
C	5.71506000	1.02633900	1.83724000
C	4.38996600	0.95557300	1.41365800
C	3.92957900	-0.16823800	0.69373000
C	4.82641400	-1.20982000	0.40760000
C	1.88945200	-1.48762700	-0.19363800
C	2.51548100	-0.19003800	0.24079600
C	-0.98275100	0.41602500	0.58505100
C	-0.39126200	-0.30761800	-0.45496900
C	0.35706000	-1.57301300	-0.14380300
C	-1.70956700	1.58830600	0.29715000
C	-1.83652100	2.02016900	-1.03880100
C	-1.27020900	1.26704800	-2.07511200
C	-0.56008500	0.09995200	-1.78670600
O	-0.19487100	-2.61595800	0.07953600
H	6.84033800	-1.95164700	0.61729100

H	7.63678200	0.03611900	1.88767500
H	6.05961900	1.89075100	2.40377100
H	3.69860600	1.76085200	1.66610700
H	4.50148100	-2.09677800	-0.13879000
H	-0.86959800	0.07583000	1.63178500
H	-2.03881900	2.23299400	1.12449300
H	-2.36360600	2.95132400	-1.26507300
H	-1.40473600	1.58229700	-3.11022100
H	-0.15629300	-0.50781400	-2.60206700
N	2.82090300	0.75915800	-1.38546700
H	2.05980300	0.52784400	-2.04893700
C	2.89979100	2.20806600	-1.17998900
C	1.79547500	2.93055100	-0.69999400
C	4.25673400	4.09825100	-1.22128200
C	1.97035800	4.30033700	-0.47063500
H	0.83547800	2.44985900	-0.50640300
C	3.20786600	4.89510000	-0.72745400
H	5.24593100	4.52052400	-1.44593200
H	1.13942900	4.89815500	-0.08912600
H	3.36335700	5.95867400	-0.55015800
N	4.11309100	2.76998500	-1.45262800
H	3.71226100	0.39406300	-1.78402400
H	2.30758000	-2.35549400	0.39652900
I	2.35791300	-2.21298200	-2.22636600
H	1.83639400	0.63335600	0.54765400
I	1.58027500	-0.77516000	2.65683900
I	-6.51096100	-0.31052300	0.14661100
I	-4.06774200	0.56123400	0.08865300

Intermediary 2 (Int2)

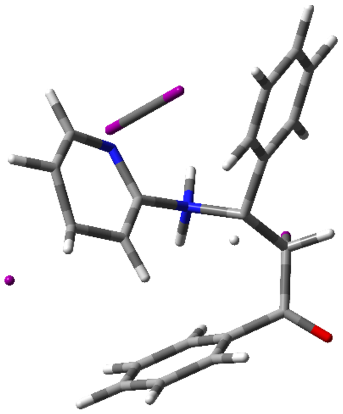

	(Hartree/Particle)
Zero-point correction=	0.315685
Thermal correction to Energy=	0.343425
Thermal correction to Enthalpy=	0.344369
Thermal correction to Gibbs Free Energy=	0.248257
Sum of electronic and zero-point Energies=	0.442336
Sum of electronic and thermal Energies=	0.470076
Sum of electronic and thermal Enthalpies=	0.471020
Sum of electronic and thermal Free Energies=	0.374908

Charge: 0 Multiplicity:1

C	-0.27006100	-4.53412800	-1.38053300
C	1.07895300	-4.49546700	-1.00562600
C	1.55258900	-3.44726400	-0.21369100
C	0.68086100	-2.43922100	0.20814400
C	-0.67158600	-2.47674400	-0.16645900
C	-1.14427200	-3.52712000	-0.96843300
C	-2.95417300	-1.77805000	0.74604300
C	-1.56505800	-1.34599600	0.28865300
C	-2.45912500	1.10648600	2.78801000
C	-3.30093700	0.54620900	1.81582400
C	-3.56191000	-0.92248600	1.87192700
C	-2.18623500	2.47714100	2.76153700
C	-2.75803500	3.28771600	1.77479400
C	-3.63094600	2.73119800	0.83200900
C	-3.91471700	1.36361400	0.85738500
O	-4.15682700	-1.46572400	2.76518500
H	-0.63787100	-5.35147100	-2.00055600
H	1.76086900	-5.28022900	-1.33624400
H	2.61417000	-3.39552200	0.07337400

H	1.07802800	-1.62088200	0.81782100
H	-2.18868600	-3.57379900	-1.27766700
H	-2.02760600	0.47954000	3.57059200
H	-1.52854700	2.91672800	3.51391000
H	-2.52494200	4.35564900	1.74052400
H	-4.09243500	3.37156500	0.07822100
H	-4.63785600	0.94625400	0.15360100
N	-1.62862300	-0.31553700	-0.86647300
C	-0.42549900	0.57869300	-0.92236900
C	-0.06223900	1.38803900	0.10113400
C	1.36157000	1.11762300	-2.29361400
C	1.07978800	2.29567700	-0.13066400
H	-0.57604500	1.45370800	1.05730600
C	1.96734300	1.88110800	-1.22542000
H	1.88344400	1.02020400	-3.25874200
H	1.59678500	2.65241700	0.78198000
H	2.73857300	2.61372100	-1.51670900
N	0.20132400	0.47311700	-2.17286200
H	-1.70308400	-0.83620500	-1.78564600
H	-2.87451300	-2.83307600	1.14132600
I	-4.49045900	-1.92877500	-0.75860300
H	-1.06957300	-0.82451800	1.16725300
H	-2.48739400	0.28104900	-0.77962400
I	-0.05669000	4.21981800	-0.65029500
I	4.71405800	-1.60736400	0.94993600
I	3.32412000	0.28623400	-0.24705200

Intermediary 3 (Int3)

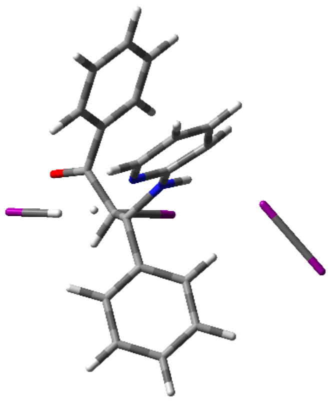

	(Hartree/Particle)
Zero-point correction=	0.309006
Thermal correction to Energy=	0.338708
Thermal correction to Enthalpy=	0.339653
Thermal correction to Gibbs Free Energy=	0.235783
Sum of electronic and zero-point Energies=	0.411604
Sum of electronic and thermal Energies=	0.441307
Sum of electronic and thermal Enthalpies=	0.442251
Sum of electronic and thermal Free Energies=	0.338382

Charge: 0 Multiplicity:1

C	-0.22816800	0.61436100	4.20394500
C	1.09794600	0.16779200	4.24324900
C	1.65285900	-0.45975700	3.12559400
C	0.88707800	-0.64552900	1.96906900
C	-0.43350100	-0.18404100	1.92305400
C	-0.99218800	0.44362900	3.04813100
C	-2.15063200	-1.60131900	0.77072100
C	-1.30155700	-0.33081200	0.68136800
C	-3.42507200	0.05373600	-2.00209100
C	-3.11598300	-1.25506500	-1.60625600
C	-3.34432400	-1.65222600	-0.18242000
C	-3.23483500	0.43482800	-3.33269100
C	-2.74837400	-0.48840400	-4.26485000
C	-2.45598100	-1.79701800	-3.86803800
C	-2.64206000	-2.18584100	-2.53775800
O	-4.42639200	-1.97232900	0.24460300
H	-0.66453900	1.10049900	5.07539200
H	1.69566700	0.31183300	5.14200600
H	2.68933200	-0.80125800	3.14406500

H	1.34261400	-1.14539000	1.11313000
H	-2.02223000	0.80245900	3.02836300
H	-3.81615200	0.77311500	-1.27762200
H	-3.47435400	1.45076200	-3.64445100
H	-2.60563200	-0.18954000	-5.30275000
H	-2.09061500	-2.51921400	-4.59700400
H	-2.42906700	-3.21255100	-2.23397600
N	-0.48827400	-0.30306600	-0.56559700
C	-0.03005600	0.85133400	-1.14538200
C	0.95281600	0.78241200	-2.20185800
C	-0.13892700	3.19056800	-1.34134500
C	1.28807400	1.99500200	-2.86623400
H	1.10127500	-0.16908200	-2.73312800
C	0.77639600	3.20064100	-2.42111700
H	-0.57132100	4.13317000	-0.97268000
H	1.95826700	1.95619900	-3.73166000
H	1.06078900	4.14227400	-2.88330400
N	-0.55415700	2.07386600	-0.71874500
H	-2.54063600	-1.68634700	1.82242400
I	-1.07616300	-3.46415000	0.51859700
H	-2.02417300	0.54930500	0.63697400
H	-0.07269800	-1.20154800	-0.85040300
I	5.16295600	0.07109100	0.43001200
I	3.03759500	0.55845100	-1.00266400
I	-3.82375900	2.63433200	1.21123900
H	-2.24277100	2.89727400	0.79691900

Transition state (TS2)

Imaginary frequency: -501.65 cm^{-1}

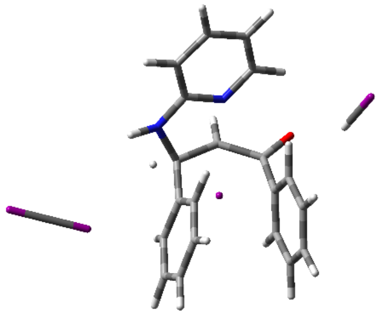

	**(Hartree/Particle)**
Zero-point correction=	0.307380
Thermal correction to Energy=	0.336176
Thermal correction to Enthalpy=	0.337120
Thermal correction to Gibbs Free Energy=	0.237862
Sum of electronic and zero-point Energies=	0.448596
Sum of electronic and thermal Energies=	0.477392
Sum of electronic and thermal Enthalpies=	0.478336
Sum of electronic and thermal Free Energies=	0.379078

Charge: 0 Multiplicity:1

C	-2.03337300	-2.27363500	1.38412800
C	-1.65536500	-1.86222600	2.67044200
C	-0.84785400	-0.73716300	2.82859500
C	-0.41117700	-0.00978100	1.70914300
C	-0.77208700	-0.41465100	0.42419200
C	-1.61407900	-1.54499300	0.25451700
C	1.07759200	0.17936400	-1.32339600
C	-0.36280300	0.37300100	-0.80866200
C	1.80049700	-2.42080300	0.92669500
C	2.20906000	-1.10410800	0.69830400
C	2.26799600	-0.50760700	-0.67125400
C	1.85693000	-2.95072200	2.22135000
C	2.33120800	-2.17292400	3.27949900
C	2.75393400	-0.85744200	3.04730800
C	2.69952700	-0.32277900	1.76020000
O	3.35467100	-0.38717300	-1.22480100
H	-2.62770400	-3.18284600	1.25302600

H	-1.99153000	-2.42594600	3.54098200
H	-0.54269800	-0.41827400	3.82693000
H	0.23244100	0.85778200	1.85793400
H	-1.74578300	-1.97917700	-0.75627700
H	1.44320400	-3.04245300	0.09477600
H	1.53895200	-3.97793100	2.39638100
H	2.38438200	-2.59086700	4.28415400
H	3.14193400	-0.25706500	3.86899000
H	3.04932600	0.69457700	1.57908900
N	-0.61864300	1.83396900	-0.56963500
C	0.51971800	2.60335200	-0.39035200
C	0.57547400	3.96833600	-0.02568300
C	2.86784400	2.52252000	-0.75281300
C	1.81855000	4.58825700	-0.04260700
H	-0.32986200	4.50936700	0.23727700
C	2.97187500	3.86829300	-0.40873600
H	3.74104100	1.91247100	-1.05513100
H	1.90542700	5.64411600	0.22769500
H	3.94518600	4.35853400	-0.42515500
N	1.65810600	1.88390300	-0.71662800
H	1.25217700	0.53150100	-2.36624100
I	0.65930100	-2.07060200	-2.35605800
H	-1.06360300	0.10422700	-1.66909600
H	-1.51373600	2.09024700	-0.15097500
I	-5.96031200	1.13553800	-0.03188800
I	-3.82244500	-0.30791700	0.26317400
I	6.09899100	0.65989200	0.27316300
H	5.04305700	-0.39800500	-0.49203500

Intermediary 4 (Int4)

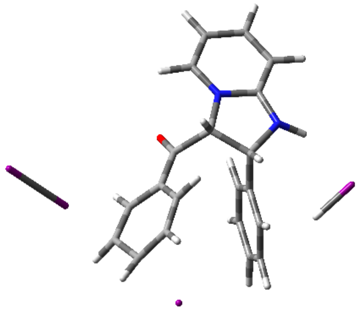

	(Hartree/Particle)
Zero-point correction=	0.310046
Thermal correction to Energy=	0.338818
Thermal correction to Enthalpy=	0.339762
Thermal correction to Gibbs Free Energy=	0.239350
Sum of electronic and zero-point Energies=	0.429662
Sum of electronic and thermal Energies=	0.458433
Sum of electronic and thermal Enthalpies=	0.459378
Sum of electronic and thermal Free Energies=	0.358966

Charge: 0 Multiplicity:1

C	2.80413100	2.54262200	0.08790700
C	3.03179500	2.36070700	1.45488300
C	3.04611600	1.06938600	1.99556300
C	2.83843700	-0.03815900	1.17182700
C	2.61855200	0.14284000	-0.20323000
C	2.59688100	1.43590400	-0.74271900
C	1.21811600	-1.98180300	-0.73164200
C	2.40281800	-1.03678900	-1.11916000
C	-0.61288500	0.25720000	-1.32106800
C	-0.56417300	-0.31088800	0.01405300
C	0.24407500	-1.42800700	0.36991800
C	-1.29239100	1.40014000	-1.56981200
C	-1.96869100	2.13483500	-0.49109800
C	-2.23324100	1.35322300	0.72629800
C	-1.39629800	0.24701100	0.99996600
O	0.25512600	-2.04037300	1.42773600
H	2.75955800	3.55073400	-0.33200200
H	3.16494000	3.22654900	2.10507300
H	3.19395600	0.92948200	3.06687800

H	2.81296000	-1.03612500	1.60954500
H	2.38856300	1.59604900	-1.80128500
H	-0.11589300	-0.27315800	-2.12894000
H	-1.35002900	1.83103500	-2.56792600
H	-2.79609700	2.79560700	-0.81155100
H	-2.65774100	1.91605000	1.57339500
H	-1.40436800	-0.19950000	2.00441500
N	3.61224200	-1.94871300	-1.09675400
C	3.30463100	-3.12902300	-0.44373900
C	4.13276200	-4.19928300	-0.05058500
C	1.33726100	-4.29904700	0.38394300
C	3.54696700	-5.30375200	0.55290800
H	5.20960500	-4.14091600	-0.21777800
C	2.15439700	-5.34993500	0.77550700
H	0.24805400	-4.26411700	0.57173100
H	4.16948500	-6.14793300	0.87054900
H	1.71014500	-6.21555200	1.27624200
N	1.91227200	-3.22018700	-0.25356300
H	0.60603000	-2.23541900	-1.63725600
H	2.27257900	-0.69484600	-2.18230700
H	4.54492700	-1.50845500	-1.00632000
I	-0.44706100	3.78621300	0.05028100
I	-4.11922100	0.02863600	0.24328100
I	6.41332400	0.35796900	-0.27068300
H	5.24614800	1.28994500	0.43986800
I	-6.26158900	-1.40144600	-0.28628200

Intermediary 5 (Int5)

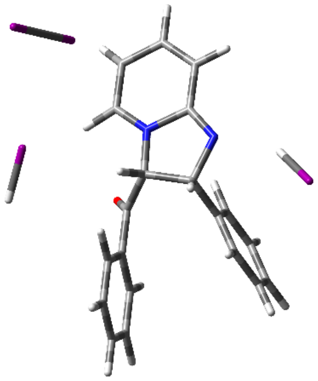

	(Hartree/Particle)
Zero-point correction=	0.305776
Thermal correction to Energy=	0.335987
Thermal correction to Enthalpy=	0.336931
Thermal correction to Gibbs Free Energy=	0.229881
Sum of electronic and zero-point Energies=	0.407398
Sum of electronic and thermal Energies=	0.437609
Sum of electronic and thermal Enthalpies=	0.438553
Sum of electronic and thermal Free Energies=	0.331503

Charge: 0 Multiplicity:1

C	5.69237000	1.01956400	0.34449700
C	5.83479000	1.11899900	1.73187600
C	4.77513100	0.75599600	2.56976000
C	3.57466700	0.29617100	2.02302200
C	3.42824900	0.20290900	0.63115000
C	4.49298300	0.56103600	-0.20810900
C	0.87478800	0.57319500	0.37398000
C	2.14203900	-0.29961400	0.01573400
C	1.65134600	2.99065400	-1.22940700
C	1.87550000	2.91804100	0.15433800
C	1.16046600	1.93771000	1.01421900
C	2.33484600	3.93708600	-1.99415000
C	3.24625000	4.80837100	-1.38412700
C	3.46860300	4.73906800	-0.00591900
C	2.78075400	3.79887000	0.76660600
O	0.77966800	2.18692000	2.13523900
H	6.52026400	1.29039400	-0.30966000
H	6.77208300	1.47126700	2.15999900

H	4.88687100	0.82478000	3.65108300
H	2.75625400	0.00424100	2.68094500
H	4.39757500	0.47506600	-1.29099300
H	0.94034200	2.31923300	-1.71310100
H	2.16059400	3.99763000	-3.06844600
H	3.78199700	5.54290200	-1.98678100
H	4.17667700	5.41818000	0.46905700
H	2.94429000	3.74286300	1.84605300
N	1.83908300	-1.67627800	0.51749100
C	0.73147700	-1.68730400	1.20366800
C	0.01828700	-2.73765100	1.89202600
C	-1.01102500	-0.07200600	1.97194900
C	-1.14777700	-2.47177500	2.53594900
H	0.46085400	-3.73460500	1.85291700
C	-1.72355500	-1.14042100	2.56382900
H	-1.34965700	0.98028300	2.05879000
H	-1.69591000	-3.26390600	3.05644600
H	-2.42943600	-0.91540000	3.37875900
N	0.11197500	-0.34560200	1.26578900
H	0.25938200	0.74772300	-0.55843000
H	2.23728300	-0.36239700	-1.10121000
I	4.84784600	-2.92339800	-1.45978100
I	-2.51542300	2.07147100	-0.68248400
I	-3.39358500	-1.14524100	0.84501000
H	3.64733300	-2.80166600	-0.31870400
H	-1.85515800	3.57151900	-0.80884100
I	-5.04652700	-0.83573100	-1.14403000

Transition state (TS3)

Imaginary frequency: -1316.20 cm^{-1}

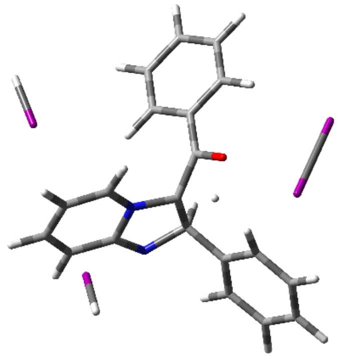

	**(Hartree/Particle)**
Zero-point correction=	0.301909
Thermal correction to Energy=	0.332069
Thermal correction to Enthalpy=	0.333013
Thermal correction to Gibbs Free Energy=	0.227906
Sum of electronic and zero-point Energies=	0.490554
Sum of electronic and thermal Energies=	0.520714
Sum of electronic and thermal Enthalpies=	0.521658
Sum of electronic and thermal Free Energies=	0.416551

Charge: 0 Multiplicity:1

C	2.27594000	-3.79903200	0.06888700
C	2.57672300	-4.50580700	-1.08997900
C	2.10354200	-4.04630800	-2.33091600
C	1.35047600	-2.87652500	-2.41625700
C	1.04759100	-2.14300700	-1.25851500
C	1.53089300	-2.59470900	-0.00268900
C	-0.30671700	-0.32947000	-0.05945600
C	0.27877700	-0.83925900	-1.37352300
C	0.16441300	2.78043300	-0.50695800
C	0.79722500	2.03883600	0.51348300
C	0.41485100	0.66748900	0.74404600
C	0.55786300	4.09462000	-0.74946700
C	1.58929800	4.66969700	0.00569600
C	2.22573500	3.93587800	1.01253300
C	1.83309800	2.62266600	1.27254000
O	0.73180700	0.01574900	1.81480900
H	2.60122500	-4.16923400	1.04370000

H	3.16882600	-5.41852600	-1.03992300
H	2.32567100	-4.61528500	-3.23606300
H	0.96500500	-2.53195700	-3.38192400
H	1.07008100	-2.18735300	0.92208000
H	-0.63438100	2.32207500	-1.10008300
H	0.06972100	4.67228000	-1.53417100
H	1.90098200	5.69607900	-0.19707600
H	3.03652800	4.38368100	1.58796700
H	2.33023500	2.03353400	2.05139200
N	-0.89005100	-1.00102700	-2.30043600
C	-1.98994400	-0.65579500	-1.67063000
C	-3.36725800	-0.61312600	-2.08708300
C	-2.71133600	0.11974200	0.58383800
C	-4.33701600	-0.22614400	-1.20756400
H	-3.58101000	-0.90487300	-3.11366400
C	-4.02449900	0.14404000	0.13925700
H	-2.43834300	0.38347100	1.61169400
H	-5.38469700	-0.19211200	-1.51686300
H	-4.82829300	0.44090100	0.81130800
N	-1.72338400	-0.23112900	-0.29109400
H	0.97395300	-0.07932900	-1.83149900
H	0.21794600	-1.06420000	1.36324600
I	-4.66551600	-2.57635700	1.22637600
I	-3.80791200	3.08991400	-0.44255600
I	3.45406900	-1.05070500	0.11354700
H	-3.76240400	4.71874200	-0.64062000
I	5.15987200	0.88334700	0.46838100
H	-5.09653100	-4.00263200	1.91463400

Intermediary 6 (Int6)

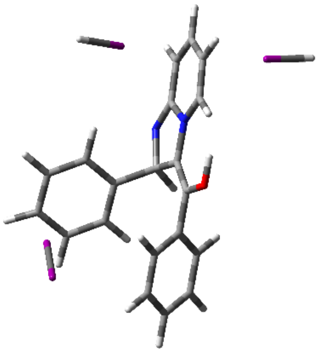

	(Hartree/Particle)
Zero-point correction=	0.303542
Thermal correction to Energy=	0.334576
Thermal correction to Enthalpy=	0.335520
Thermal correction to Gibbs Free Energy=	0.227020
Sum of electronic and zero-point Energies=	0.423323
Sum of electronic and thermal Energies=	0.454356
Sum of electronic and thermal Enthalpies=	0.455300
Sum of electronic and thermal Free Energies=	0.346800

Charge: 0 Multiplicity:1

C	-2.41122500	-1.42033100	-2.78336500
C	-2.59156700	-0.03332000	-2.99978500
C	-1.55627900	0.85779000	-2.62707400
C	-0.40153300	0.37937100	-2.01134100
C	-0.24396900	-0.99747400	-1.78234200
C	-1.25285100	-1.89438900	-2.16920100
C	1.13226900	-1.05542600	0.33691300
C	1.01284600	-1.53377300	-1.12136000
C	-0.67122000	-3.63340400	0.85655100
C	-0.86247800	-2.29221400	1.22797800
C	0.30258900	-1.38706200	1.34952400
C	-1.77509700	-4.47914200	0.74055300
C	-3.06353500	-3.99380000	0.99937100
C	-3.24875500	-2.66341700	1.38280300
C	-2.14843900	-1.80782700	1.50363200
O	0.39007800	-0.94020400	2.64302500
H	-3.18259100	-2.12128400	-3.11182700
H	-3.39449400	0.31368700	-3.66474900

H	-1.66320500	1.92483200	-2.83693300
H	0.39020200	1.07307900	-1.71793000
H	-1.13481600	-2.96393200	-1.98918300
H	0.33286600	-4.01353000	0.67452900
H	-1.63405400	-5.52044800	0.45573600
H	-3.92236300	-4.65737600	0.90567900
H	-4.25240400	-2.28258500	1.58782300
H	-2.29028800	-0.76921200	1.81213100
N	2.22471600	-1.00443800	-1.82165000
C	2.92001500	-0.27870500	-0.98077500
C	4.13978500	0.47601700	-1.15577000
C	2.84925700	0.52839100	1.37619900
C	4.63614400	1.22594300	-0.13276600
H	4.59912600	0.43544800	-2.14577500
C	3.98557400	1.26510200	1.14822500
H	2.33594100	0.52192600	2.34878400
H	5.54411800	1.82238000	-0.26205700
H	4.43727400	1.84881300	1.94817000
N	2.31814200	-0.24026600	0.35845000
H	1.05009200	-2.65374500	-1.17165700
H	1.13663200	-0.29604500	2.78992100
I	6.20525200	-1.57529500	0.10514700
I	2.30026600	3.70481200	0.32805300
H	7.30424900	-2.70465300	0.56608300
H	1.40638300	5.05682100	0.05861500
I	-3.93043000	0.32395000	-0.93450800
I	-5.45230500	0.70967300	1.14098700

Intermediary 7 (Int7)

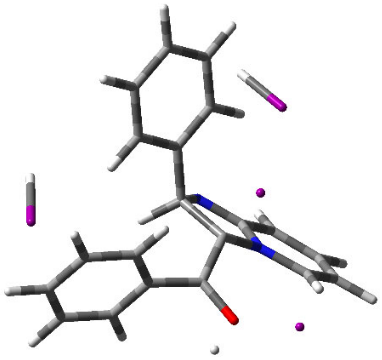

	(Hartree/Particle)
Zero-point correction=	0.303784
Thermal correction to Energy=	0.333647
Thermal correction to Enthalpy=	0.334591
Thermal correction to Gibbs Free Energy=	0.233178
Sum of electronic and zero-point Energies=	0.442178
Sum of electronic and thermal Energies=	0.472041
Sum of electronic and thermal Enthalpies=	0.472985
Sum of electronic and thermal Free Energies=	0.371572

Charge: 0 Multiplicity:1

C	1.29827800	3.58590200	-0.00270000
C	0.55946300	4.29492700	0.94883700
C	0.03403100	3.62467100	2.05937100
C	0.23670600	2.25129700	2.21328600
C	0.96450500	1.53335600	1.24966600
C	1.50666900	2.21192200	0.14764900
C	-0.00204000	-0.83870000	0.81355700
C	1.19122700	0.04249300	1.38372900
C	1.22390500	-0.24198200	-2.21899100
C	1.39059200	-1.29538000	-1.30833600
C	0.33623200	-1.67093800	-0.34758900
C	2.23967500	0.05092900	-3.13320600
C	3.41684300	-0.70366700	-3.14420300
C	3.58200700	-1.75912800	-2.23890700
C	2.57194500	-2.05795100	-1.32174000
O	-0.48923100	-2.67184000	-0.68900200
H	1.72306300	4.10555900	-0.85988500
H	0.41841100	5.36826300	0.84173600

H	-0.52191000	4.17601100	2.81623000
H	-0.16872600	1.73571800	3.08616100
H	2.09400600	1.67211500	-0.59710800
H	0.29703500	0.34453900	-2.21764300
H	2.11000100	0.86967100	-3.84160600
H	4.20585400	-0.47321800	-3.86128400
H	4.49366900	-2.35840000	-2.25801200
H	2.70521100	-2.87493900	-0.60982700
N	1.34044500	-0.34377200	2.81265000
C	0.40710800	-1.20783600	3.13581200
C	0.10894500	-1.84535600	4.39674000
C	-1.54715800	-2.40561000	2.14674100
C	-0.95191900	-2.69198900	4.49709200
H	0.76238900	-1.60268700	5.23336200
C	-1.79410600	-2.97181900	3.36726800
H	-2.17454500	-2.58725300	1.24684000
H	-1.19241200	-3.17730500	5.44529200
H	-2.64002700	-3.64283100	3.49580600
N	-0.44473500	-1.56656200	2.00586200
H	2.15285700	-0.24006000	0.86242300
H	-0.19285800	-3.14262200	-1.63317300
I	-1.62348900	0.49017000	-0.04364500
I	-2.95040700	2.92054700	-0.70519800
I	-2.51884400	-2.81071900	-1.31234600
I	4.81778800	0.23851200	-0.06395500
H	-1.68452500	3.68733300	0.06196400
H	5.19143000	1.01527400	1.33936900

Transition state 4 (TS4)

Imaginary frequency: -1060.40 cm^{-1}

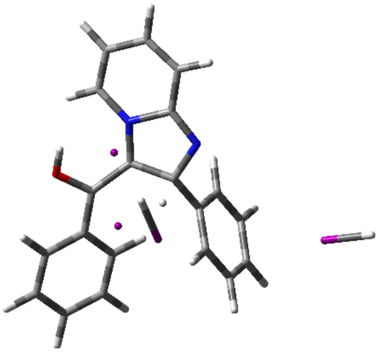

	(Hartree/Particle)
Zero-point correction=	0.296672
Thermal correction to Energy=	0.326713
Thermal correction to Enthalpy=	0.327658
Thermal correction to Gibbs Free Energy=	0.225014
Sum of electronic and zero-point Energies=	0.489487
Sum of electronic and thermal Energies=	0.519528
Sum of electronic and thermal Enthalpies=	0.520472
Sum of electronic and thermal Free Energies=	0.417828

Charge: 0 Multiplicity:1

C	-2.70930400	0.17857100	-1.51361200
C	-3.37325200	-0.55002700	-0.52075000
C	-2.83496600	-0.62225900	0.76903300
C	-1.62832100	0.01650500	1.06322500
C	-0.94483300	0.72006400	0.05687000
C	-1.49924300	0.81755400	-1.22817100
C	1.61208500	0.61925600	0.63179200
C	0.34729200	1.37039800	0.38383100
C	0.67990800	-1.51112100	-1.58121400
C	1.96657900	-0.93757600	-1.53442700
C	2.39774100	-0.00088200	-0.50039800
C	0.34194900	-2.40100100	-2.60016900
C	1.27595000	-2.73633300	-3.58450800
C	2.55990100	-2.17418000	-3.55199400
C	2.90420900	-1.28156000	-2.54346700
O	3.73401100	0.11276500	-0.48257100
H	-3.13415000	0.24884500	-2.51490200
H	-4.30445300	-1.06755700	-0.75122100

H	-3.36003500	-1.17747500	1.54741900
H	-1.22269100	-0.01945400	2.07601200
H	-0.99740400	1.39189600	-2.00923900
H	-0.07007100	-1.27906300	-0.81582200
H	-0.65794000	-2.84450700	-2.61425700
H	1.00718400	-3.43596300	-4.37826100
H	3.28675600	-2.43249300	-4.32267600
H	3.90139600	-0.83047900	-2.53056700
N	0.25702900	2.44761700	1.33302600
C	1.38983000	2.51039700	2.00414400
C	1.84421500	3.39728500	3.05228500
C	3.47325100	1.16461500	2.31993900
C	3.02722100	3.16131200	3.66994300
H	1.17916500	4.22476800	3.31324000
C	3.84054200	2.01625500	3.31422500
H	4.02206500	0.24922400	2.07147000
H	3.39303000	3.81313100	4.46572700
H	4.75381100	1.84084100	3.88493700
N	2.31094700	1.45540400	1.56954700
H	4.09617900	0.92418400	0.00050000
I	1.50469100	-1.55695500	1.65385000
I	2.00894400	2.26013800	-2.21003700
H	0.72236500	2.09702700	-0.83691600
I	-4.72321200	2.28096700	0.00382500
I	-1.47805900	-3.98465700	0.49557100
H	-0.26014200	-2.94639600	1.05218200
H	-5.78282400	3.50340300	0.27539700

Intermediary 8 (Int8)

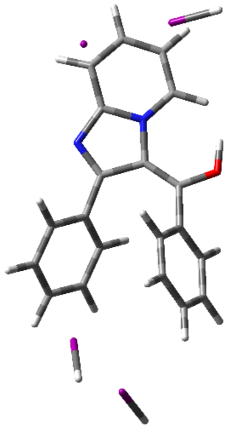

	(Hartree/Particle)
Zero-point correction=	0.299425
Thermal correction to Energy=	0.330648
Thermal correction to Enthalpy=	0.331592
Thermal correction to Gibbs Free Energy=	0.222872
Sum of electronic and zero-point Energies=	0.446706
Sum of electronic and thermal Energies=	0.477928
Sum of electronic and thermal Enthalpies=	0.478872
Sum of electronic and thermal Free Energies=	0.370152

Charge: 0 Multiplicity:1

C	-3.05644600	-0.32718800	2.09475100
C	-3.39617600	-1.61326500	1.65905300
C	-2.42394300	-2.43461600	1.07929100
C	-1.10751100	-1.98230700	0.95125200
C	-0.76219000	-0.69977800	1.40825400
C	-1.74285900	0.13021900	1.97770300
C	1.16219100	1.06406600	0.95402600
C	0.64122000	-0.27380300	1.37587000
C	-1.13944900	1.03637100	-1.22831200
C	-0.82916300	2.03084900	-0.28541400
C	0.53155900	2.07570400	0.28600500
C	-2.40212900	1.02551600	-1.82282000
C	-3.35573200	1.98560000	-1.46638900
C	-3.04479100	2.97099900	-0.52253900
C	-1.77721600	3.00610400	0.06230100
O	1.07908300	3.28320800	0.02233800
H	-3.81953600	0.31498300	2.53569500
H	-4.41428700	-1.98101300	1.80074200

H	-2.68287200	-3.44082200	0.74782600
H	-0.34014100	-2.62999800	0.52094500
H	-1.47596400	1.12167400	2.33949600
H	-0.39830700	0.27959100	-1.49384600
H	-2.64463100	0.26285200	-2.56538100
H	-4.34663200	1.96506600	-1.92483300
H	-3.79307000	3.71279500	-0.24500600
H	-1.52204800	3.78371500	0.78194400
N	1.62623600	-1.05068600	1.81646700
C	2.82429100	-0.30975200	1.74951000
C	4.17464000	-0.74828200	1.99013300
C	3.51384600	1.95947400	1.29527000
C	5.11805400	0.34337200	2.16896600
H	4.28393300	-1.64819100	2.62133000
C	4.81586600	1.61828200	1.77509900
H	3.29793000	2.96852300	0.94796500
H	6.10715400	0.09287800	2.55633900
H	5.55580200	2.41833700	1.83290300
N	2.56214900	0.99292600	1.30413200
H	2.04534900	3.38384500	0.31545500
I	4.95256900	-1.76917200	0.00833000
I	5.92611500	0.72231000	-1.43422500
I	-6.37400100	0.76277100	0.38436100
I	-4.75999300	-1.67312300	-1.26463700
H	-5.93354500	-1.79883800	-2.41122500
H	-7.29054000	1.90831800	1.13295700
H	6.48709700	2.01187400	-2.30123000

Product (P)

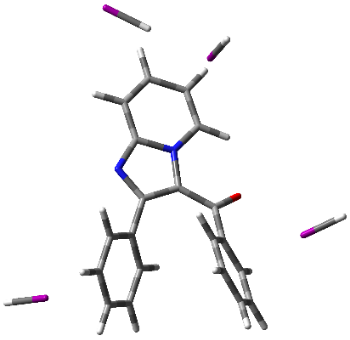

	(Hartree/Particle)
Zero-point correction=	0.295211
Thermal correction to Energy=	0.328248
Thermal correction to Enthalpy=	0.329192
Thermal correction to Gibbs Free Energy=	0.210978
Sum of electronic and zero-point Energies=	0.406843
Sum of electronic and thermal Energies=	0.439880
Sum of electronic and thermal Enthalpies=	0.440824
Sum of electronic and thermal Free Energies=	0.322610

Charge: 0 Multiplicity:1

C	-4.47452200	1.52609800	0.18845200
C	-4.53235400	1.88351400	1.54061200
C	-3.35438200	1.98388600	2.28893200
C	-2.11935800	1.71908100	1.69309200
C	-2.06188000	1.34144700	0.34070400
C	-3.24275400	1.25874700	-0.41503200
C	-0.40191000	0.01275000	-1.16510900
C	-0.76318500	1.07020700	-0.28580800
C	-2.30278300	-1.74333200	0.46430300
C	-2.30606000	-1.65870000	-0.93490000
C	-1.11302200	-1.15959900	-1.68123800
C	-3.41756900	-2.26074100	1.13121100
C	-4.53352100	-2.69009700	0.40307700
C	-4.53230700	-2.61477700	-0.99396600
C	-3.41702300	-2.10765500	-1.66501000
O	-0.72272400	-1.73144800	-2.68705000
H	-5.39189100	1.44561700	-0.39454600
H	-5.49491200	2.08303000	2.01070400
H	-3.39871000	2.27727900	3.33686800

H	-1.19608000	1.81671800	2.26724500
H	-3.19859100	0.98877700	-1.47011500
H	-1.43464000	-1.40413300	1.03225800
H	-3.42380600	-2.31117400	2.22008400
H	-5.40390300	-3.08647500	0.92618100
H	-5.39672600	-2.95937700	-1.55951100
H	-3.39843000	-2.06647800	-2.75609700
N	0.30296400	1.92674700	-0.07450200
C	1.34889100	1.45566500	-0.82726100
C	2.67947200	1.92707500	-1.00532400
C	1.79471700	-0.39953700	-2.39316000
C	3.51769000	1.24823600	-1.85287500
H	2.98609100	2.82286400	-0.45678300
C	3.07554100	0.07872600	-2.54485200
H	1.39156100	-1.28370200	-2.91644800
H	4.53984000	1.59951500	-2.02124900
H	3.76257100	-0.42006000	-3.23119000
N	0.93745200	0.28162100	-1.53536100
I	6.02443200	1.95633600	0.93959700
I	-4.22260000	4.69974500	-0.06530700
I	4.16352700	-1.58452400	-0.05207500
I	-2.21105200	-5.14458400	0.68699700
H	5.25939600	0.48841500	0.69017100
H	-4.35603200	6.27186400	-0.51469300
H	4.79781100	-2.56767400	1.10565500
H	-1.58500100	-6.65756400	0.79356800

DES: ChCl:Tartaric acid (1:2)

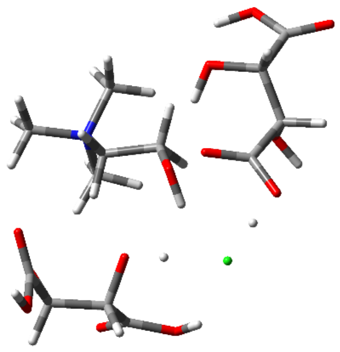

	(Hartree/Particle)
Zero-point correction=	0.372197
Thermal correction to Energy=	0.408135
Thermal correction to Enthalpy=	0.409080
Thermal correction to Gibbs Free Energy=	0.299475
Sum of electronic and zero-point Energies=	–0.626428
Sum of electronic and thermal Energies=	–0.590490
Sum of electronic and thermal Enthalpies=	–0.589546
Sum of electronic and thermal Free Energies=	–0.699151

Charge: 0 Multiplicity:1

C	5.86717800	0.31985100	-0.40708900
O	3.67459300	1.20129500	-0.63236200
C	4.43428400	0.28422400	0.16585100
C	2.36106900	-1.13394900	0.50940300
O	6.81684600	-0.24653200	0.06609800
O	6.02620900	1.11358400	-1.49962600
O	1.62716300	-0.21422200	0.12348200
O	1.97195900	-2.14307300	1.20913800
H	2.88967500	0.72374900	-1.04773500
H	5.15401200	1.51991500	-1.81734600
H	0.75933200	-2.17468800	1.50026100
O	-0.80387300	0.96605200	3.25551400
N	-0.54648900	2.37378700	-0.23553600
H	-1.16773000	0.04867600	3.21046500
C	-4.49538900	0.87116100	-0.02672900
O	-3.58138500	0.36352400	-2.10479500
C	-4.19524500	-0.27693400	-0.99119200
C	-2.70055500	-2.28605100	-1.37047000

O	-5.53183600	0.48218600	0.78109900
O	-3.99459900	1.96179400	0.09509200
O	-2.88910900	-2.16711800	-2.56185700
O	-1.97134200	-3.34584800	-0.95068200
H	-5.78642000	1.17054400	1.45769900
H	-1.77215300	-3.39362500	0.02945200
Cl	-0.69827000	-2.28364900	1.94248400
C	-0.08925800	1.27955900	2.06195800
H	0.74366200	1.91832300	2.40984300
H	0.31037900	0.36388500	1.57841100
C	-1.07585700	2.04107000	1.15621400
H	-2.01194200	1.42424000	1.04402900
H	-1.39571900	2.97125700	1.67649100
C	-0.63937000	1.17172600	-1.14237400
H	-1.70341000	0.86300300	-1.29687400
H	-0.18580800	1.35417700	-2.12497400
H	-0.13433100	0.28372000	-0.69110300
C	-1.41102600	3.47652200	-0.81511600
H	-1.13759000	3.70167800	-1.85840600
H	-2.48406900	3.16339000	-0.82066300
H	-1.33893500	4.40576200	-0.22934900
C	0.88856700	2.84405600	-0.17377300
H	1.22618100	3.25413700	-1.13943200
H	1.03456300	3.61765900	0.59605100
H	1.57917100	1.99382300	0.06232600
H	-3.40632000	-0.32239400	-2.82812000
H	-5.14487500	-0.76920500	-1.32399600
H	4.43383800	0.71636500	1.19730200
C	-3.26499100	-1.31698500	-0.30556700

H	-3.82806700	-1.88123900	0.47845200
O	-2.20881600	-0.59070300	0.27297400
H	-1.60884800	-1.20622800	0.92117000
O	3.98342500	-1.61805300	-1.17892800
H	3.98473600	-2.60630400	-1.19178300
C	3.86961300	-1.14647000	0.16670900
H	4.43867600	-1.80486200	0.86622500

IRC Graphs of Transition States in Gas Phase

IRC Transition state 1 (TS1)

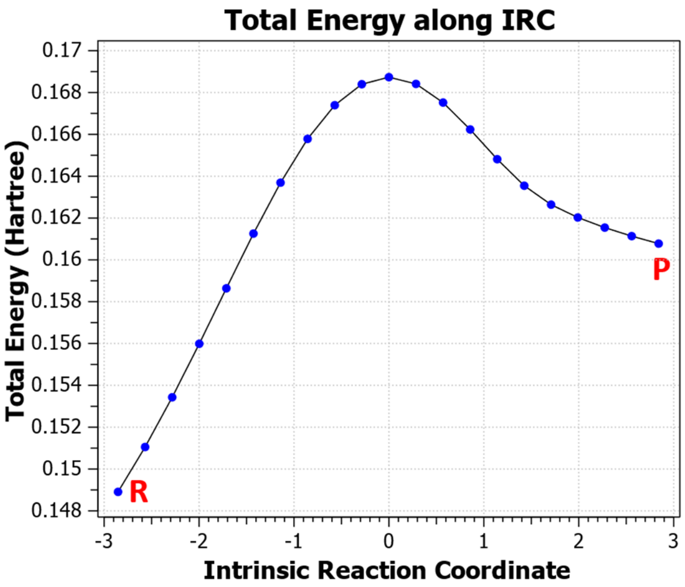

Charge: 0 Multiplicity:1

C	6.37858820	-1.03402465	0.81516482
C	6.82594020	0.08004235	1.52826482
C	5.93838920	1.12514835	1.81606682
C	4.61329520	1.05438235	1.39248482
C	4.15290820	-0.06942865	0.67255682
C	5.04974320	-1.11101065	0.38642682
C	2.11278120	-1.38881765	-0.21481118
C	2.73881020	-0.09122865	0.21962282
C	-0.75942180	0.51483435	0.56387782
C	-0.16793280	-0.20880865	-0.47614218
C	0.58038920	-1.47420365	-0.16497618
C	-1.48623780	1.68711535	0.27597682
C	-1.61319180	2.11897835	-1.05997418
C	-1.04687980	1.36585735	-2.09628518
C	-0.33675580	0.19876135	-1.80787918
O	0.02845820	-2.51714865	0.05836282

H	7.06366720	-1.85283765	0.59611782
H	7.86011120	0.13492835	1.86650182
H	6.28294820	1.98956035	2.38259782
H	3.92193520	1.85966135	1.64493382
H	4.72481020	-1.99796865	-0.15996318
H	-0.64626880	0.17463935	1.61061182
H	-1.81548980	2.33180335	1.10331982
H	-2.14027680	3.05013335	-1.28624618
H	-1.18140680	1.68110635	-3.13139418
H	0.06703620	-0.40900465	-2.62324018
N	3.04423220	0.85796735	-1.40664018
H	2.28313220	0.62665335	-2.07011018
C	3.12312020	2.30687535	-1.20116218
C	2.01880420	3.02936035	-0.72116718
C	4.48006320	4.19706035	-1.24245518
C	2.19368720	4.39914635	-0.49180818
H	1.05880720	2.54866835	-0.52757618
C	3.43119520	4.99390935	-0.74862718
H	5.46926020	4.61933335	-1.46710518
H	1.36275820	4.99696435	-0.11029918
H	3.58668620	6.05748335	-0.57133118
N	4.33642020	2.86879435	-1.47380118
H	3.93559020	0.49287235	-1.80519718
H	2.53090920	-2.25668465	0.37535582
I	2.58124220	-2.11417265	-2.24753918
H	2.05972320	0.73216535	0.52648082
I	1.80360420	-0.67635065	2.63566582
I	-6.28763180	-0.21171365	0.12543782
I	-3.84441280	0.66004335	0.06747982

IRC Transition state 2 (TS2)

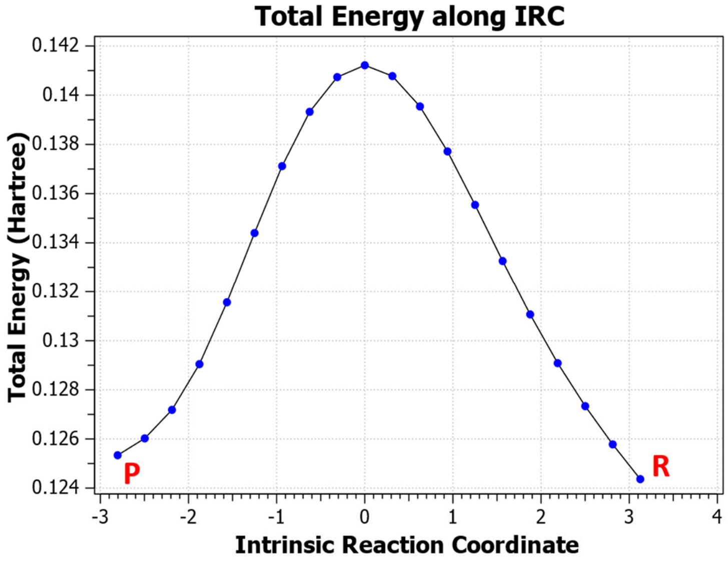

Charge: 0 Multiplicity:1

C	-1.93340446	-2.25562356	1.45183118
C	-1.55539646	-1.84421456	2.73814518
C	-0.74788546	-0.71915156	2.89629818
C	-0.31120846	0.00823044	1.77684618
C	-0.67211846	-0.39663956	0.49189518
C	-1.51411046	-1.52698156	0.32222018
C	1.17756054	0.19737544	-1.25569282
C	-0.26283446	0.39101244	-0.74095882
C	1.90046554	-2.40279156	0.99439818
C	2.30902854	-1.08609656	0.76600718
C	2.36796454	-0.48959556	-0.60355082
C	1.95689854	-2.93271056	2.28905318
C	2.43117654	-2.15491256	3.34720218
C	2.85390254	-0.83943056	3.11501118
C	2.79949554	-0.30476756	1.82790318
O	3.45463954	-0.36916156	-1.15709782

H	-2.52773546	-3.16483456	1.32072918
H	-1.89156146	-2.40793456	3.60868518
H	-0.44272946	-0.40026256	3.89463318
H	0.33240954	0.87579344	1.92563718
H	-1.64581446	-1.96116556	-0.68857382
H	1.54317254	-3.02444156	0.16247918
H	1.63892054	-3.95991956	2.46408418
H	2.48435054	-2.57285556	4.35185718
H	3.24190254	-0.23905356	3.93669318
H	3.14929454	0.71258844	1.64679218
N	-0.51867446	1.85198044	-0.50193182
C	0.61968654	2.62136344	-0.32264882
C	0.67544254	3.98634744	0.04202018
C	2.96781254	2.54053144	-0.68510982
C	1.91851854	4.60626844	0.02509618
H	-0.22989346	4.52737844	0.30498018
C	3.07184354	3.88630444	-0.34103282
H	3.84100954	1.93048244	-0.98742782
H	2.00539554	5.66212744	0.29539818
H	4.04515454	4.37654544	-0.35745182
N	1.75807454	1.90191444	-0.64892482
H	1.35214554	0.54951244	-2.29853782
I	0.75926954	-2.05259056	-2.28835482
H	-0.96363446	0.12223844	-1.60139282
H	-1.41376746	2.10825844	-0.08327182
I	-5.86034346	1.15354944	0.03581518
I	-3.72247646	-0.28990556	0.33087718
I	6.19895954	0.67790344	0.34086618
H	5.14302554	-0.37999356	-0.42433182

IRC Transition state 3 (TS3)

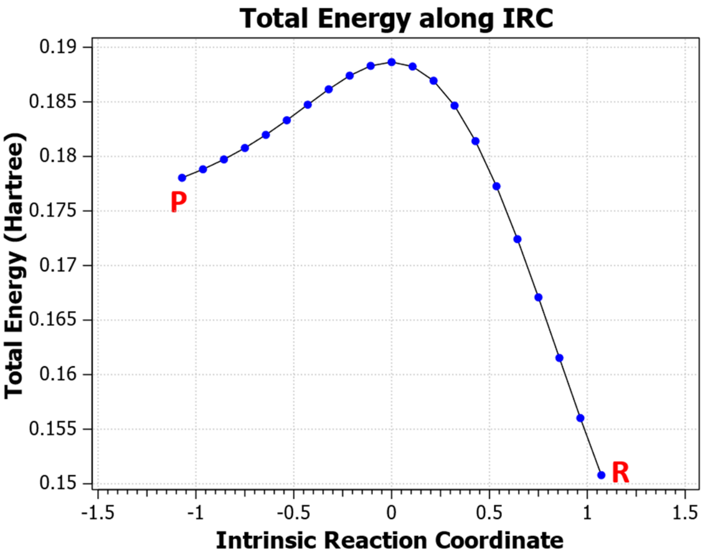

Charge: 0 Multiplicity:1

C	2.26367030	-3.80859378	0.02595469
C	2.56445330	-4.51536878	-1.13291131
C	2.09127230	-4.05586978	-2.37384831
C	1.33820630	-2.88608678	-2.45918931
C	1.03532130	-2.15256878	-1.30144731
C	1.51862330	-2.60427078	-0.04562131
C	-0.31898670	-0.33903178	-0.10238831
C	0.26650730	-0.84882078	-1.41645531
C	0.15214330	2.77087122	-0.54989031
C	0.78495530	2.02927422	0.47055069
C	0.40258130	0.65792722	0.70111369
C	0.54559330	4.08505822	-0.79239931
C	1.57702830	4.66013522	-0.03723631
C	2.21346530	3.92631622	0.96960069
C	1.82082830	2.61310422	1.22960769
O	0.71953730	0.00618722	1.77187669

H	2.58895530	-4.17879578	1.00076769
H	3.15655630	-5.42808778	-1.08285531
H	2.31340130	-4.62484678	-3.27899531
H	0.95273530	-2.54151878	-3.42485631
H	1.05781130	-2.19691478	0.87914769
H	-0.64665070	2.31251322	-1.14301531
H	0.05745130	4.66271822	-1.57710331
H	1.88871230	5.68651722	-0.24000831
H	3.02425830	4.37411922	1.54503469
H	2.31796530	2.02397222	2.00845969
N	-0.90232070	-1.01058878	-2.34336831
C	-2.00221370	-0.66535678	-1.71356231
C	-3.37952770	-0.62268778	-2.13001531
C	-2.72360570	0.11018022	0.54090569
C	-4.34928570	-0.23570578	-1.25049631
H	-3.59327970	-0.91443478	-3.15659631
C	-4.03676870	0.13447822	0.09632469
H	-2.45061270	0.37390922	1.56876169
H	-5.39696670	-0.20167378	-1.55979531
H	-4.84056270	0.43133922	0.76837569
N	-1.73565370	-0.24069078	-0.33402631
H	0.96168330	-0.08889078	-1.87443131
H	0.20567630	-1.07376178	1.32031369
I	-4.67778570	-2.58591878	1.18344369
I	-3.82018170	3.08035222	-0.48548831
I	3.44179930	-1.06026678	0.07061469
H	-3.77467370	4.70918022	-0.68355231
I	5.14760230	0.87378522	0.42544869
H	-5.10880070	-4.01219378	1.87170169

IRC Transition state 4 (TS4)

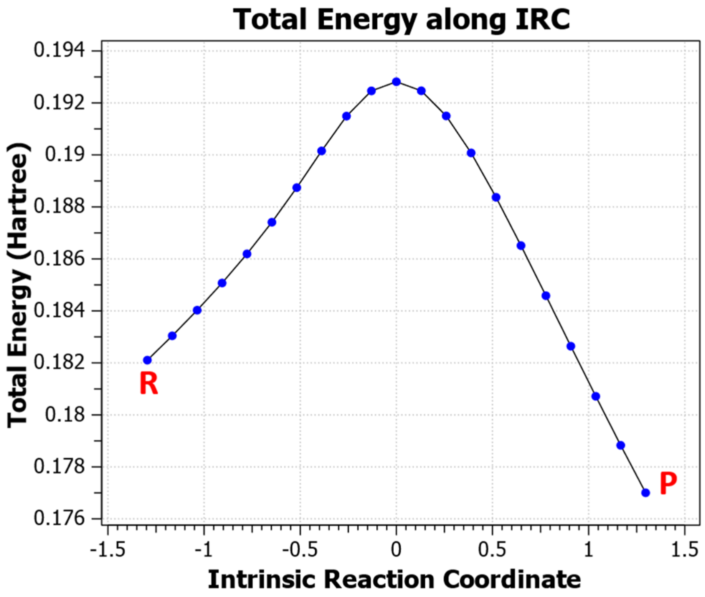

Charge: 0 Multiplicity:1

C	-2.63186637	0.20715028	-1.51471738
C	-3.29581437	-0.52144772	-0.52185538
C	-2.75752837	-0.59367972	0.76792762
C	-1.55088337	0.04508428	1.06211962
C	-0.86739537	0.74864328	0.05576462
C	-1.42180537	0.84613328	-1.22927638
C	1.68952263	0.64783528	0.63068662
C	0.42472963	1.39897728	0.38272562
C	0.75734563	-1.48254172	-1.58231938
C	2.04401663	-0.90899672	-1.53553238
C	2.47517863	0.02769728	-0.50150338
C	0.41938663	-2.37242172	-2.60127438
C	1.35338763	-2.70775372	-3.58561338
C	2.63733863	-2.14560072	-3.55309938
C	2.98164663	-1.25298072	-2.54457238
O	3.81144863	0.14134428	-0.48367638

H	-3.05671237	0.27742428	-2.51600738
H	-4.22701537	-1.03897772	-0.75232638
H	-3.28259737	-1.14889572	1.54631362
H	-1.14525337	0.00912528	2.07490662
H	-0.91996637	1.42047528	-2.01034438
H	0.00736663	-1.25048372	-0.81692738
H	-0.58050237	-2.81592772	-2.61536238
H	1.08462163	-3.40738372	-4.37936638
H	3.36419363	-2.40391372	-4.32378138
H	3.97883363	-0.80189972	-2.53167238
N	0.33446663	2.47619628	1.33192062
C	1.46726763	2.53897628	2.00303862
C	1.92165263	3.42586428	3.05117962
C	3.55068863	1.19319428	2.31883362
C	3.10465863	3.18989128	3.66883762
H	1.25660263	4.25334728	3.31213462
C	3.91797963	2.04483428	3.31311962
H	4.09950263	0.27780328	2.07036462
H	3.47046763	3.84171028	4.46462162
H	4.83124863	1.86942028	3.88383162
N	2.38838463	1.48398328	1.56844162
H	4.17361663	0.95276328	-0.00060538
I	1.58212863	-1.52837572	1.65274462
I	2.08638163	2.28871728	-2.21114238
H	0.79980263	2.12560628	-0.83802138
I	-4.64577437	2.30954628	0.00271962
I	-1.40062137	-3.95607772	0.49446562
H	-0.18270437	-2.91781672	1.05107662
H	-5.70538637	3.53198228	0.27429162

Energies and cartesian coordinates in DES

Intermediary 1 (Int1)

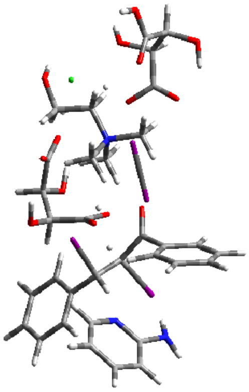

	**(Hartree/Particle)**
Zero-point correction=	0.683278
Thermal correction to Energy=	0.750816
Thermal correction to Enthalpy=	0.751760
Thermal correction to Gibbs Free Energy=	0.559676
Sum of electronic and zero-point Energies=	-0.252464
Sum of electronic and thermal Energies=	-0.184926
Sum of electronic and thermal Enthalpies=	-0.183982
Sum of electronic and thermal Free Energies=	-0.376067

Charge: 0 Multiplicity:1

C	2.46628400	4.89549900	-1.62987400
C	3.79110300	5.26996000	-1.87896200
C	4.82957700	4.36927800	-1.62158200
C	4.54853300	3.09607200	-1.11958600
C	3.21898600	2.71955800	-0.86474400
C	2.17879000	3.62782400	-1.12237200
C	2.01078100	1.29407100	0.83037000
C	2.94794500	1.35082700	-0.33952200
C	3.22666100	-1.83089100	0.82501000
C	2.34963100	-1.15112800	1.70141200
C	1.43843600	-0.06134000	1.20529000
C	4.01017400	-2.88877400	1.32661000
C	3.90343000	-3.27346200	2.66966300
C	3.03137300	-2.59536900	3.52205300
C	2.24780200	-1.53611400	3.03935200
O	0.24255500	-0.24098000	1.15553500
H	1.64590600	5.58411300	-1.84008500

H	4.01262100	6.25912600	-2.27573300
H	5.86094200	4.65863200	-1.81568900
H	5.36212300	2.39610600	-0.92770700
H	1.13272700	3.34988500	-0.94001400
H	3.46762700	-1.41130000	-0.17260500
H	4.72549300	-3.38924400	0.66767100
H	4.50818700	-4.09909600	3.04689600
H	2.95067200	-2.88970900	4.57028900
H	1.55664100	-1.02070500	3.71117900
N	6.24764300	-0.36653100	1.94327100
H	6.89742500	-0.11540600	2.67280600
C	6.75393600	-0.49062900	0.64158300
C	8.10418600	-0.85992600	0.40492300
C	6.27672100	-0.53897200	-1.65370800
C	8.50526600	-1.07322700	-0.90903900
H	8.79780000	-0.98481800	1.22958000
C	7.58899800	-0.91658000	-1.95948400
H	5.51905500	-0.39520600	-2.43775400
H	9.53522000	-1.36273600	-1.12498800
H	7.89150000	-1.08161000	-2.98914800
N	5.84847400	-0.31591500	-0.38262000
H	5.32860700	0.05962700	2.04798600
H	1.14231500	2.01522700	0.69404800
I	3.02660400	2.03713600	2.60254400
H	3.89810500	0.78039300	-0.15016700
I	2.06301500	0.17865700	-2.00284800
C	-1.17121500	4.13497600	0.68568500
O	-0.83985100	4.73225900	-1.63733800
C	-1.41312900	5.17315800	-0.42762800

C	-3.72906500	4.20693100	-0.95289800
O	-0.50508200	3.13278800	0.56603800
O	-1.74509000	4.39088000	1.88583200
O	-3.27320400	3.30453500	-1.64957300
O	-4.89896300	4.23422800	-0.39839100
H	-1.32990100	3.91770100	-1.97983300
H	-2.40304400	5.18101300	1.87938500
H	-5.64356800	3.23491200	-0.61081800
O	-5.28871500	-0.04984100	-3.10445900
N	-2.62731600	-0.52362500	-0.47537400
H	-6.06986800	0.36314100	-2.66585400
C	-6.57301700	-2.79440600	0.44213500
C	-7.30080500	-2.25841100	1.68934500
C	-5.72192300	-0.25881000	1.98743800
O	-7.46905800	-3.46663000	-0.33804500
O	-5.41194500	-2.70577900	0.11489000
O	-4.98850500	-0.84512800	2.77178200
O	-5.30838200	0.75227400	1.27049400
H	-7.06517400	-3.84017400	-1.17054900
H	-6.08064800	1.21886500	0.46821100
Cl	-6.62746300	2.10147300	-0.73115500
C	-4.08750000	0.31313900	-2.42457500
H	-3.30215200	0.14605700	-3.18501500
H	-4.10988200	1.38628400	-2.13529100
C	-3.95450200	-0.62697200	-1.21244900
H	-4.78722300	-0.42237800	-0.49144700
H	-4.12330000	-1.68170000	-1.54238400
C	-2.45904600	0.85529500	0.11303500
H	-3.27428600	1.08278200	0.83520500

H	-1.49588200	0.95260200	0.66115900
H	-2.47125400	1.64125200	-0.66576900
C	-2.60348100	-1.54238600	0.64551000
H	-1.60898300	-1.56810800	1.13458000
H	-3.35002000	-1.29667700	1.43263800
H	-2.84091400	-2.55769900	0.28087500
C	-1.48204700	-0.81889000	-1.41546300
H	-0.51313000	-0.81599700	-0.86565800
H	-1.58579500	-1.81615700	-1.88172200
H	-1.40197700	-0.06329800	-2.21358200
H	-8.37413900	-2.58361600	1.66339900
H	-0.85114800	6.11344800	-0.18816900
C	-7.18502500	-0.71697400	1.78050000
H	-7.81541400	-0.33956900	2.61651800
O	-7.56952200	-0.16123400	0.52542200
H	-8.45685700	0.26343200	0.57166100
O	-3.35731400	6.02515300	0.63714000
H	-4.33509700	5.81691200	0.76608000
C	-2.91614400	5.47116200	-0.61226100
H	-3.06753300	6.24494200	-1.40371000
O	-6.78305100	-2.88366600	2.83800900
H	-5.86001000	-2.52437100	3.06818300
I	-0.43546800	-4.64449300	-1.37834400
I	1.47694800	-3.35822800	-0.17994200

Transition State 1 (TS1)

Imaginary frequency: -450.06 cm^{-1}

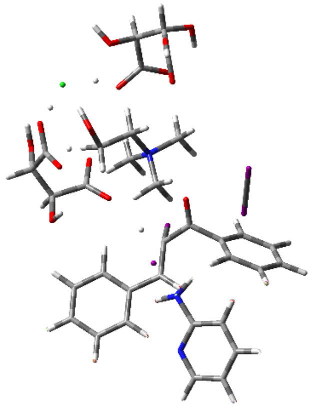

	(Hartree/Particle)
Zero-point correction=	0.684072
Thermal correction to Energy=	0.750197
Thermal correction to Enthalpy=	0.751142
Thermal correction to Gibbs Free Energy=	0.564910
Sum of electronic and zero-point Energies=	-0.198960
Sum of electronic and thermal Energies=	-0.132835
Sum of electronic and thermal Enthalpies=	-0.131891
Sum of electronic and thermal Free Energies=	-0.318122

Charge: 0 Multiplicity:1

C	3.31511300	-4.43358500	1.53888600
C	4.41538300	-4.45903200	2.39977500
C	5.22549200	-3.32381300	2.52905500
C	4.93441200	-2.16886000	1.80540800
C	3.83461100	-2.13886200	0.92059400
C	3.02991200	-3.28589400	0.79731700
C	2.32482200	-0.83220600	-0.69789100
C	3.54241800	-0.89233000	0.18079600
C	2.87687500	2.56815100	-0.03353500
C	2.53407400	1.73004400	-1.11788000
C	1.65979500	0.52442500	-0.88711100
C	3.60804800	3.74181700	-0.27351000
C	3.97946400	4.08390300	-1.58342500
C	3.63273600	3.25088000	-2.64596600
C	2.90600700	2.07066900	-2.41701000
O	0.45639300	0.61995500	-0.92208500
H	2.64958600	-5.29723300	1.46073100

H	4.63538500	-5.35471600	2.98010700
H	6.07611300	-3.33811100	3.20925600
H	5.55019700	-1.27987000	1.94106000
H	2.13974300	-3.28250300	0.15352500
H	2.70737000	2.20554900	1.01797300
H	3.90672000	4.37996500	0.56362800
H	4.52837400	5.00842700	-1.76706000
H	3.91284200	3.51924000	-3.66646800
H	2.61356100	1.44211400	-3.26308300
N	5.05346000	-1.05864800	-1.06425900
H	4.74879700	-0.74298300	-2.00609100
C	6.23640800	-0.31685800	-0.63370200
C	6.22682000	1.08758900	-0.59205600
C	8.40528400	-0.43065000	0.20631200
C	7.37523500	1.72901800	-0.11477300
H	5.35926100	1.66363300	-0.91529500
C	8.47622800	0.97128800	0.29182100
H	9.24632500	-1.07043800	0.50746500
H	7.40632900	2.81933300	-0.06049000
H	9.37700900	1.45336800	0.66945900
N	7.30505100	-1.07675900	-0.25324800
H	5.25119500	-2.07786500	-1.11941000
H	1.52535900	-1.55405400	-0.30204000
I	2.45411900	-1.61167100	-2.74174400
H	4.00832500	0.06110500	0.49415700
I	2.04251100	0.06057500	2.18683700
C	-0.38812400	-4.07706100	-0.95145600
O	0.24157400	-4.88175100	1.24653400
C	-0.39811000	-5.25169700	0.04645800

C	-2.78082400	-4.64791200	0.81252000
O	0.19074400	-3.02916500	-0.78147000
O	-1.06256300	-4.26668900	-2.11029100
O	-2.41648600	-3.73140500	1.53757200
O	-3.97490000	-4.83121100	0.33659600
H	-0.26986800	-4.14172600	1.70252900
H	-1.63503700	-5.12320400	-2.12968400
H	-4.83981700	-3.97443900	0.64301200
O	-4.94799900	-0.80857300	3.28070000
N	-2.47238000	0.26108300	0.65258500
H	-5.66520700	-1.32199100	2.84053600
C	-6.74170200	1.82982200	-0.25547100
C	-7.35872800	1.21285600	-1.52474600
C	-5.47235400	-0.49496800	-1.85427700
O	-7.74614400	2.32122500	0.52921700
O	-5.58621300	1.93079100	0.08617200
O	-4.84954800	0.21244400	-2.63460300
O	-4.89668200	-1.42898800	-1.14584600
H	-7.41847200	2.73170600	1.37726100
H	-5.58585600	-2.02331800	-0.34885700
Cl	-5.98707000	-3.00757000	0.82635600
C	-3.72701800	-0.93362100	2.55194300
H	-2.95449700	-0.66446700	3.29729200
H	-3.57603400	-1.98203400	2.21653300
C	-3.79534200	0.06043200	1.37767700
H	-4.56660400	-0.28257600	0.64216800
H	-4.17588300	1.04417700	1.74768200
C	-2.00298100	-1.04285500	0.05381000
H	-2.76181800	-1.44978800	-0.64949300

H	-1.05838200	-0.91237500	-0.51744900
H	-1.81427500	-1.81116000	0.82755800
C	-2.66221200	1.26705200	-0.46349600
H	-1.68839200	1.54555900	-0.91353400
H	-3.29432000	0.85576100	-1.27991200
H	-3.15799600	2.18843100	-0.10530300
C	-1.43392700	0.79574600	1.60928100
H	-0.46879300	0.98964000	1.08461400
H	-1.75162500	1.74800500	2.06592100
H	-1.20592600	0.07849500	2.41755000
H	-8.47096200	1.35742000	-1.50582900
H	0.23723800	-6.08537200	-0.35210500
C	-6.99205100	-0.28654500	-1.64757500
H	-7.54866500	-0.74334400	-2.49586900
O	-7.28831200	-0.92171200	-0.40662200
H	-8.08152500	-1.50105700	-0.47366500
O	-2.29192900	-6.26797000	-0.94322800
H	-3.29944700	-6.21741900	-0.97094500
C	-1.82947400	-5.75631500	0.31871800
H	-1.81560700	-6.59911500	1.05111200
O	-6.93944500	1.94254800	-2.65172200
H	-5.96965900	1.73933000	-2.88250600
I	-1.61094600	4.96037400	0.71805500
I	0.71670400	3.92032900	0.24391000

Intermediary 2 (Int2)

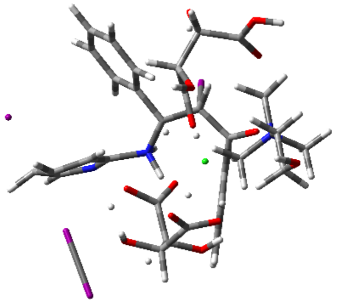

	(Hartree/Particle)
Zero-point correction=	0.689167
Thermal correction to Energy=	0.753824
Thermal correction to Enthalpy=	0.754768
Thermal correction to Gibbs Free Energy=	0.574978
Sum of electronic and zero-point Energies=	-0.241547
Sum of electronic and thermal Energies=	-0.176889
Sum of electronic and thermal Enthalpies=	-0.175945
Sum of electronic and thermal Free Energies=	-0.355735

Charge: 0 Multiplicity:1

C	1.97470900	4.40505600	-1.92115400
C	1.01738000	4.38298900	-2.93863300
C	-0.12850200	3.59120400	-2.80090600
C	-0.31287900	2.81769000	-1.65410100
C	0.64715300	2.84424800	-0.62899100
C	1.78946900	3.64453200	-0.76176600
C	-0.39743200	2.74963000	1.65065000
C	0.38774600	1.98308200	0.58813000
C	0.08484700	-0.17846700	3.72393500
C	0.13638000	1.22626800	3.71271200
C	-0.85488700	1.95946600	2.87681800
C	0.98984500	-0.90603000	4.49872100
C	1.92690000	-0.23742700	5.29321100
C	1.95106400	1.16148900	5.31578500
C	1.05492300	1.89462700	4.53382700
O	-2.04710700	1.91249700	3.10262600

H	2.87743900	5.00662800	-2.03160500
H	1.16600500	4.97494600	-3.84123200
H	-0.87678200	3.57085100	-3.59301600
H	-1.20915700	2.19045800	-1.56845600
H	2.55554100	3.67358300	0.01409600
H	-0.64396300	-0.71996900	3.10611500
H	0.95887900	-1.99963900	4.47609900
H	2.63585200	-0.80700000	5.89715500
H	2.66997200	1.68292800	5.94872100
H	1.06679700	2.98672500	4.58330600
N	1.66455600	1.36059300	1.17400200
C	2.68386400	0.94499500	0.17767200
C	2.41087700	0.24009500	-0.96299200
C	4.98616900	0.93404700	-0.12730600
C	3.51826400	-0.30204900	-1.71698300
H	1.38744600	0.02562400	-1.29899800
C	4.83828700	0.31661000	-1.48208900
H	6.00580800	1.15513300	0.22203800
H	3.31413400	-0.56535000	-2.76940000
H	5.69645400	-0.31156100	-1.79521800
N	3.98419100	1.25329300	0.64436900
H	2.11055100	2.02690400	1.85544300
H	-1.32435300	3.13570800	1.12987000
I	0.49402200	4.58612700	2.34850700
H	-0.25896000	1.08877700	0.26273200
H	1.38491200	0.49744100	1.71605800

C	-0.06440600	-1.83402800	0.42982300
O	-0.64711900	-3.11580500	2.38737400
C	-0.10156900	-3.21412300	1.09306600
C	-2.33288600	-3.89509500	0.05757200
O	-0.27287700	-0.77416400	1.00585500
O	0.23809400	-1.76435500	-0.87748200
O	-3.02140300	-3.41837500	0.95404400
O	-2.75360700	-4.14794000	-1.15706600
H	-1.64885300	-3.04035100	2.34795400
H	0.48344900	-2.68788200	-1.31437100
H	-3.84261300	-3.76936400	-1.39784400
O	-7.12844900	-2.40270500	0.87339700
N	-4.53828800	0.18847300	1.69918300
H	-7.07187200	-2.66306200	-0.07744200
C	-6.54474800	1.70040400	-2.18290000
C	-5.54293100	1.75630500	-3.35268500
C	-3.76706300	0.39150700	-2.10846700
O	-7.81164400	1.78573300	-2.67845000
O	-6.35240500	1.61239900	-0.98985200
O	-3.16394700	1.39444000	-1.71069100
O	-3.74071500	-0.73830900	-1.50346800
H	-8.51752800	1.75699200	-1.97420200
H	-4.53889200	-1.71626200	-1.95742100
Cl	-5.17092900	-3.04322300	-1.93505000
C	-5.84889000	-1.99985100	1.35573800
H	-5.92545900	-2.14835400	2.44904600

H	-5.04699200	-2.65008200	0.94249200
C	-5.66696800	-0.52093700	0.96393300
H	-5.47501100	-0.44776500	-0.14101700
H	-6.62784400	0.02333000	1.12220800
C	-3.23016300	-0.51532000	1.44289200
H	-3.02176800	-0.58560200	0.34393100
H	-2.37671900	0.01242800	1.89947800
H	-3.23849900	-1.55453500	1.82113300
C	-4.44683600	1.61328500	1.19679500
H	-3.74797400	2.21193100	1.81111200
H	-4.07849900	1.64470500	0.14392400
H	-5.43310000	2.10927900	1.18532900
C	-4.80410200	0.22179100	3.18903800
H	-3.98962000	0.76305500	3.71783700
H	-5.75265000	0.73553300	3.41740600
H	-4.85850200	-0.79460500	3.61488200
H	-6.10225100	1.88348000	-4.31654500
H	0.98207900	-3.57479400	1.26764200
C	-4.65107700	0.49235600	-3.37679100
H	-4.00908000	0.49629400	-4.28511100
O	-5.49278500	-0.65839800	-3.32185100
H	-5.56123600	-1.10058800	-4.19872400
O	-0.17774500	-4.26542600	-1.03306600
H	-0.80371800	-4.56770000	-1.75544300
C	-0.85008800	-4.25418600	0.23189400
H	-0.74765900	-5.27582100	0.68100600

O	-4.77304400	2.92929200	-3.24445900
H	-4.05922200	2.83330200	-2.52500400
I	5.10816200	2.09781700	-2.83900500
I	3.48163000	-2.38769400	-0.75350200
I	3.13636200	-4.68612800	0.50405800

Intermediary 3 (Int3)

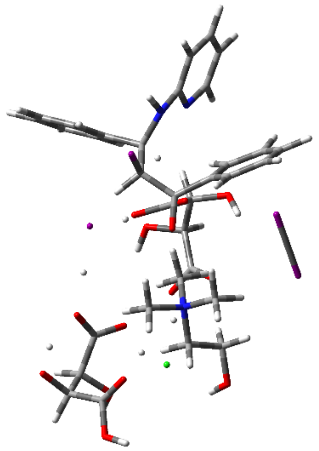

	(Hartree/Particle)
Zero-point correction=	0.683448
Thermal correction to Energy=	0.749917
Thermal correction to Enthalpy=	0.750862
Thermal correction to Gibbs Free Energy=	0.566073
Sum of electronic and zero-point Energies=	-0.276325
Sum of electronic and thermal Energies=	-0.209855
Sum of electronic and thermal Enthalpies=	-0.208911
Sum of electronic and thermal Free Energies=	-0.393699

Charge: 0 Multiplicity:1

C	3.67538200	-5.25104000	0.97124900
C	3.21830200	-5.26964600	2.29209800
C	2.76707800	-4.08744500	2.88794800
C	2.76616000	-2.89397900	2.16222800
C	3.20994500	-2.87626400	0.83003400
C	3.67567600	-4.05798200	0.24192500
C	2.51116400	-1.64242000	-1.27555500
C	3.18974500	-1.54519400	0.09335200
C	2.89347600	1.62461200	-0.60691800
C	2.69849200	0.91042400	-1.80738500
C	1.90128200	-0.35739400	-1.80351200
C	3.53446200	2.87868800	-0.63877100
C	4.02514900	3.38104400	-1.86970300
C	3.85154100	2.65476100	-3.03929200
C	3.17978300	1.41679700	-3.01249900
O	0.77002600	-0.36913700	-2.24755400

H	4.02934600	-6.16907900	0.50512600
H	3.21147800	-6.20129600	2.85495000
H	2.41094300	-4.09711400	3.91688400
H	2.42420200	-1.97039900	2.63473200
H	4.03636800	-4.06442100	-0.78732400
H	2.60892400	1.17097400	0.36488700
H	3.83493200	3.36656200	0.29936500
H	4.54156600	4.34276700	-1.88353400
H	4.23237100	3.03604900	-3.98697600
H	3.04230600	0.85816600	-3.94184000
N	4.56234100	-0.98239400	-0.00543500
C	5.11999000	-0.27746300	1.07541900
C	6.51466900	0.00282100	1.08446900
C	4.80090100	0.98708800	3.02100900
C	7.01862400	0.80400600	2.10189300
H	7.16583200	-0.39465300	0.31339900
C	6.16106900	1.31296100	3.08744800
H	4.08555900	1.35756300	3.76693100
H	8.08396000	1.03694400	2.13527800
H	6.54216300	1.93979300	3.88661600
N	4.27034900	0.20212700	2.04815900
H	1.69757200	-2.42324500	-1.19912800
I	3.70978300	-2.30733000	-2.97018700
H	2.55003100	-0.81077200	0.70021400
H	5.21206800	-1.54633900	-0.56348500
C	0.38033700	0.06273300	2.12725300

O	0.64972900	2.29598300	3.08050900
C	0.28065500	0.96550800	3.36837600
C	-2.28461100	1.24901000	3.07941700
O	0.88864200	0.43869500	1.09130700
O	-0.13614700	-1.21596800	2.18887400
O	-2.26275100	1.88650900	2.04897600
O	-3.39448900	0.69457300	3.58557200
H	0.22059500	2.61001800	2.22920200
H	-0.61084300	-1.40374800	3.12627000
H	-4.27771100	0.80388800	2.95565200
O	-5.41636500	2.54847500	-0.91961900
N	-2.37717700	0.47607600	-1.69421400
H	-5.97242100	2.08952500	-0.24808300
C	-6.02385200	-2.21711300	-2.31949700
C	-6.17274700	-3.32553500	-1.26028900
C	-4.31069200	-2.41610800	0.27117200
O	-7.22886200	-2.02978600	-2.93374400
O	-5.05816000	-1.56525100	-2.64655800
O	-3.46286700	-3.22602700	-0.17613700
O	-4.01763700	-1.31172900	0.80595400
H	-7.21026300	-1.32315200	-3.63655800
H	-5.12217500	-0.47861800	1.25631500
Cl	-5.70834000	0.60337200	1.93451500
C	-4.04358200	2.19377000	-0.76063200
H	-3.50253700	3.03023500	-1.24788900
H	-3.76819800	2.14574400	0.31402200

C	-3.83774100	0.84869400	-1.48246100
H	-4.33723300	0.03158200	-0.89852300
H	-4.37115000	0.86760700	-2.46178400
C	-1.65875900	0.41014700	-0.37289700
H	-2.19556500	-0.24864000	0.34527500
H	-0.62922100	0.00381600	-0.48531500
H	-1.55893200	1.40821400	0.09997800
C	-2.30728200	-0.88535300	-2.35503100
H	-1.25686100	-1.16416800	-2.57480100
H	-2.72724300	-1.67424700	-1.69518700
H	-2.89297800	-0.91813900	-3.28936200
C	-1.69473600	1.48611500	-2.58853900
H	-0.62428500	1.21564600	-2.73246400
H	-2.17199900	1.54150800	-3.57763400
H	-1.70873700	2.50080500	-2.14028900
H	-7.22234900	-3.72188500	-1.28606900
H	1.04282500	0.63374900	4.12440100
C	-5.80560900	-2.79842200	0.14631700
H	-6.05502000	-3.56521300	0.91388300
O	-6.50370500	-1.57329600	0.37367100
H	-7.28677700	-1.70629000	0.95555200
O	-1.24679000	-0.39396000	4.53088000
H	-2.19908700	-0.57917500	4.77333300
C	-1.11790200	0.94341100	4.02492300
H	-1.14743100	1.66280400	4.88252300
O	-5.38399100	-4.42950300	-1.62804500

H	-4.40065100	-4.27037700	-1.41396500
I	-1.22851000	4.77227800	-0.27740700
I	1.10967400	3.66681100	-0.49745300
I	-0.34885300	-2.98722400	0.98487600
H	-1.97237300	-3.09537400	0.38413600

Transition State 2 (TS2)

Imaginary frequency: -499.53 cm^{-1}

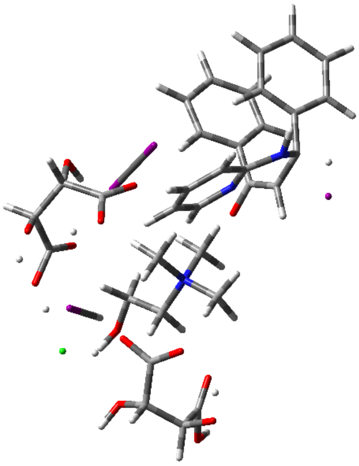

	**(Hartree/Particle)**
Zero-point correction=	0.681157
Thermal correction to Energy=	0.746881
Thermal correction to Enthalpy=	0.747825
Thermal correction to Gibbs Free Energy=	0.564678
Sum of electronic and zero-point Energies=	-0.236951
Sum of electronic and thermal Energies=	-0.171227
Sum of electronic and thermal Enthalpies=	-0.170283
Sum of electronic and thermal Free Energies=	-0.353430

Charge: 0 Multiplicity:1

C	7.81725300	-0.75237400	0.20538600
C	7.72895400	0.28701700	1.13626900
C	6.63216700	0.35478200	2.00163700
C	5.62772800	-0.61465200	1.93952000
C	5.71805500	-1.66294600	1.01193300
C	6.81815700	-1.72777900	0.14411500
C	3.36642900	-2.45530100	0.23488500
C	4.68387000	-2.76908500	0.96543400
C	4.66127000	0.46025400	-1.11324100
C	3.61133900	0.13894700	-0.25059500
C	2.85009600	-1.14699500	-0.33176100
C	5.25932600	1.73035900	-1.05576700
C	4.80390200	2.68027200	-0.14931100
C	3.72382900	2.37645300	0.71715800
C	3.14388200	1.09182200	0.67480400
O	1.69064300	-1.11511100	-0.72262600

H	8.66816300	-0.80669600	-0.47346000
H	8.51260200	1.04164600	1.18809200
H	6.55892000	1.16551200	2.72524000
H	4.76973600	-0.53978600	2.60619600
H	6.89502100	-2.53443100	-0.59100900
H	5.02652000	-0.28065900	-1.84285200
H	6.08361000	1.96383600	-1.73087200
H	5.27007700	3.66667600	-0.09619900
H	3.49085600	3.05498000	1.55153500
H	2.33844100	0.83184100	1.38055200
N	4.36651900	-3.25635700	2.35518900
C	3.16111300	-2.75847400	2.86931300
C	2.68500900	-2.92554000	4.18514600
C	1.13203800	-1.74323400	2.17377600
C	1.39033200	-2.49099600	4.46277900
H	3.30014100	-3.39976400	4.94457800
C	0.61093700	-1.89631900	3.45873300
H	0.56045800	-1.26470700	1.35616700
H	0.97844800	-2.61268500	5.46798100
H	-0.40394800	-1.53475000	3.67081600
N	2.40372300	-2.15552800	1.88265400
H	2.70166100	-3.33426300	0.07269700
I	4.19773200	-2.83998700	-2.09317200
H	5.14141200	-3.68127800	0.45972100
H	5.16688800	-3.33113000	2.98430300
C	-0.65696000	1.36882400	2.58343700

O	-0.51112600	3.73897600	2.03528400
C	-1.15171800	2.80072300	2.85829600
C	-3.23474800	2.48436800	1.37309700
O	0.30361100	1.06456600	1.91117000
O	-1.35020900	0.36435000	3.16844700
O	-2.64161800	2.55583400	0.31280200
O	-4.45497000	1.99218600	1.52155900
H	-0.68250100	3.54925700	1.05714900
H	-2.23668400	0.64028100	3.66079400
H	-4.94190100	1.60576100	0.57101200
O	-3.99018800	1.54074600	-3.58711300
N	-1.17261100	-0.38319900	-2.00391300
H	-4.83267800	1.31395700	-3.12989800
C	-4.39642300	-3.22469600	-3.47413900
C	-5.10400900	-3.94061900	-2.30919000
C	-4.08016200	-2.52284700	-0.40417900
O	-5.20834700	-3.26269600	-4.57227100
O	-3.31006200	-2.69615200	-3.53028700
O	-3.15552500	-3.31238500	-0.24218300
O	-4.06315700	-1.30036800	0.02514800
H	-4.81312800	-2.81821000	-5.37276800
H	-5.06063100	-0.44050300	-0.45448100
Cl	-5.71509900	0.81556400	-0.67237400
C	-2.88472900	1.41115500	-2.69444000
H	-2.09361600	2.02085600	-3.17588800
H	-3.13000600	1.83705500	-1.69870800

C	-2.52944700	-0.08597400	-2.62693800
H	-3.32056600	-0.63248900	-2.05393700
H	-2.57361200	-0.51878800	-3.65753400
C	-1.15346600	0.08191800	-0.56836600
H	-1.98043700	-0.38274700	0.00745100
H	-0.19319500	-0.17572300	-0.07042600
H	-1.26246000	1.18434600	-0.49371300
C	-0.92802900	-1.87697500	-2.04119300
H	0.11337000	-2.11836000	-1.74481100
H	-1.60161200	-2.41901300	-1.34235900
H	-1.11385900	-2.29784800	-3.04580400
C	-0.07090700	0.30549100	-2.77607200
H	0.91929400	0.10057000	-2.30805300
H	-0.02959400	-0.04323900	-3.81931400
H	-0.20071400	1.40633000	-2.78267400
H	-6.05982400	-4.39564400	-2.68287800
H	-0.83620800	3.09643500	3.89658200
C	-5.36975000	-2.96496000	-1.13856300
H	-6.05741100	-3.44031600	-0.40133800
O	-5.91416700	-1.75073000	-1.65540800
H	-6.89860200	-1.75756400	-1.63235300
O	-3.20261200	2.08115700	3.77500600
H	-4.14295200	1.76424400	3.54859000
C	-2.68904600	2.92497100	2.73700900
H	-3.00897900	3.97699500	2.94511200
O	-4.34008900	-5.04946500	-1.90783100

H	-3.52611700	-4.76240600	-1.36602700
I	-0.29613800	4.19012500	-1.83135300
I	1.79863600	3.19239000	-0.66941800
I	-4.36837500	-1.01982400	3.36587000
H	-3.98463800	-1.13274300	1.70607200

Intermediary 4 (Int4)

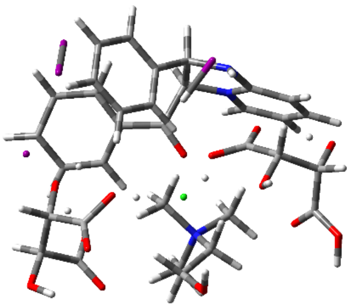

	(Hartree/Particle)
Zero-point correction=	0.684646
Thermal correction to Energy=	0.750358
Thermal correction to Enthalpy=	0.751302
Thermal correction to Gibbs Free Energy=	0.570206
Sum of electronic and zero-point Energies=	-0.274822
Sum of electronic and thermal Energies=	-0.209110
Sum of electronic and thermal Enthalpies=	-0.208166
Sum of electronic and thermal Free Energies=	-0.389262

Charge: 0 Multiplicity:1

C	-0.86535800	-0.07829100	-3.85240400
C	-0.04834300	0.93053700	-3.32880400
C	0.63001400	0.71888000	-2.12612300
C	0.48611500	-0.48601200	-1.43240900
C	-0.36876900	-1.47605200	-1.92945300
C	-1.02636700	-1.27721300	-3.15697300
C	-1.36961700	-2.65076400	0.18604500
C	-0.62387400	-2.76030200	-1.19280900
C	-2.90599700	-0.14438600	-0.55787900
C	-2.15654700	-0.22403400	0.61772500
C	-1.25710400	-1.28710800	0.94936300
C	-3.76027800	0.95497900	-0.81889000
C	-3.61368300	2.15420700	0.00898700
C	-3.04701800	1.93785200	1.34097700
C	-2.32027000	0.82764800	1.61081100
O	-0.42643600	-1.24276300	1.84797900

H	-1.38230300	0.07636500	-4.80013900
H	0.05225400	1.88024800	-3.85727100
H	1.26423100	1.51693900	-1.71096200
H	1.05299400	-0.64728500	-0.50358600
H	-1.66739000	-2.05496500	-3.57306100
H	-2.84331900	-0.93733500	-1.31022500
H	-4.11704300	1.11928700	-1.84700500
H	-4.43706300	2.88540200	-0.06007500
H	-3.21106300	2.71266500	2.08813700
H	-1.85255700	0.67124500	2.58422200
N	0.63804500	-3.52666500	-0.87918500
C	0.58685500	-4.01191100	0.40926600
C	1.49415400	-4.84688500	1.11046100
C	-1.01020400	-4.16928000	2.24717800
C	1.12209100	-5.34239900	2.34166000
H	2.47817100	-5.04166400	0.66367400
C	-0.14137400	-5.01563900	2.90512900
H	-1.97811500	-3.84974600	2.65856500
H	1.80201100	-5.99182700	2.90461200
H	-0.41688200	-5.42650200	3.87952200
N	-0.65245700	-3.68956800	0.99809900
H	-2.45067700	-2.91699000	0.07162500
H	-1.23231500	-3.45068700	-1.85128700
H	1.54209500	-3.22258500	-1.29881300
C	0.94035700	4.35860800	1.81929800
O	1.52246700	6.56950100	1.00061300

C	0.76417500	5.41909000	0.71904500
C	2.56233300	4.31034400	-0.69273700
O	1.86666400	4.29839700	2.60057300
O	0.00800400	3.37827500	1.88155700
O	3.56815000	4.94603900	-0.48524000
O	2.60268900	2.98167300	-0.96566300
H	2.49836400	6.35784600	1.05843700
H	-0.67012900	3.36554900	1.07589500
H	3.59962900	2.59043600	-1.07602500
O	5.73108600	2.47898500	2.22761200
N	2.47483100	0.64178300	2.65190900
H	6.09597500	2.31141700	1.32655100
C	5.80565800	-2.34465600	2.19172400
C	5.76713000	-3.08886700	0.84443700
C	3.78847000	-1.79949200	-0.21234000
O	7.09416500	-2.30007000	2.63642500
O	4.90790800	-1.85809800	2.84181900
O	2.98597100	-2.69857700	0.10119300
O	3.44650700	-0.61075100	-0.49042400
H	7.20090900	-1.81980900	3.50462800
H	4.52149800	0.36600500	-0.74093200
Cl	5.09774600	1.58852000	-1.08609700
C	4.30787100	2.40348600	2.21272700
H	3.99839500	3.03047900	3.07478300
H	3.89929100	2.83893500	1.28302100
C	3.94814700	0.91439300	2.38304300

H	4.26336000	0.34517300	1.46945400
H	4.56761100	0.48649600	3.20972600
C	1.65621600	0.97230800	1.42909600
H	1.91847700	0.29552900	0.57963200
H	0.56964000	0.85324900	1.62716900
H	1.81615100	2.01032300	1.08898000
C	2.30520200	-0.83017600	2.96671400
H	1.23990000	-1.08282500	3.13687300
H	2.65065100	-1.46614500	2.12370100
H	2.89833100	-1.13000300	3.84708200
C	1.98082600	1.44997500	3.82901200
H	0.95203600	1.16312500	4.10849000
H	2.63049800	1.32844700	4.70865400
H	1.93423700	2.54182200	3.58485600
H	6.78111300	-3.51664600	0.62605000
H	-0.29290800	5.80240000	0.70024700
C	5.29688800	-2.14435800	-0.28658100
H	5.51285300	-2.60131400	-1.28125800
O	5.94703500	-0.88167300	-0.13684600
H	6.74652000	-0.81736400	-0.71080700
O	0.15112400	3.73039100	-0.84583800
H	0.62978700	2.88942000	-1.23897100
C	1.12615700	4.80518600	-0.65364100
H	0.95711000	5.54687000	-1.47105800
O	4.94164300	-4.22263500	0.97037200
H	3.95431300	-3.95831400	0.94061900

I	-5.63983400	-0.26413200	-0.04450200
I	-7.70876600	-1.69051800	0.70771400
I	-1.94080300	3.46293900	-0.97489800
I	3.11365300	-2.09281200	-3.56681400
H	2.85453700	-0.68513600	-2.72829600

Intermediary 5 (Int5)

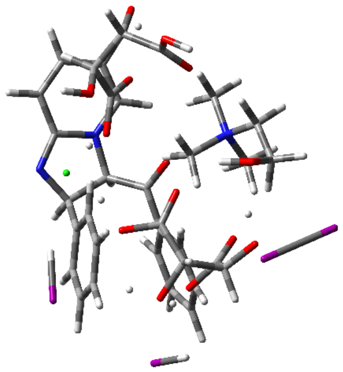

	(Hartree/Particle)
Zero-point correction=	0.679885
Thermal correction to Energy=	0.746199
Thermal correction to Enthalpy=	0.747143
Thermal correction to Gibbs Free Energy=	0.562854
Sum of electronic and zero-point Energies=	-0.262946
Sum of electronic and thermal Energies=	-0.196631
Sum of electronic and thermal Enthalpies=	-0.195687
Sum of electronic and thermal Free Energies=	-0.379976

Charge: 0 Multiplicity: 1

C	-1.76461900	0.86871700	3.76619300
C	-2.22496500	0.59859700	2.47391500
C	-1.71582600	-0.49515500	1.76710300
C	-0.74247100	-1.31593900	2.34227100
C	-0.26336300	-1.03207300	3.62890500
C	-0.78723400	0.05532900	4.34524800
C	2.23614400	-1.75513900	3.57342000
C	0.80910200	-1.87859300	4.27374900
C	2.44830200	1.29199100	3.77885100
C	2.01123800	0.63396700	2.62086700
C	2.30116600	-0.80896700	2.37360900
C	2.19140200	2.65634600	3.95669000
C	1.49375700	3.37285600	2.98229400
C	1.05817800	2.72714200	1.80341200
C	1.29992100	1.34627500	1.63964500
O	2.62997700	-1.19568700	1.27082100
H	-2.17305400	1.70762500	4.32586700

H	-2.98849900	1.23110400	2.02311700
H	-2.08671400	-0.71347800	0.76369100
H	-0.36910300	-2.18959400	1.79418800
H	-0.44392500	0.26437700	5.35619500
H	2.99597900	0.74658400	4.54963900
H	2.53599500	3.15947700	4.86069000
H	1.27251100	4.43329200	3.13008700
H	0.33733700	3.22200300	1.12363700
H	0.89962300	0.83317700	0.75227700
N	0.45011400	-3.32177500	4.23056200
C	1.38546000	-4.00043400	3.61229600
C	1.48996100	-5.40779800	3.29820900
C	3.57077700	-3.65630100	2.45685800
C	2.57392600	-5.87560200	2.62160100
H	0.66384100	-6.04068900	3.61869700
C	3.63103200	-4.99931300	2.19419600
H	4.32296100	-2.94016100	2.11622200
H	2.66449500	-6.93557000	2.37575700
H	4.46212800	-5.41742100	1.63538600
N	2.51434100	-3.16950100	3.21451600
H	2.99090500	-1.41443600	4.32819800
H	0.92764700	-1.59608600	5.35276600
C	-0.74354000	2.12664400	-1.46110200
O	-1.19059800	2.12571300	-3.86504000
C	-1.77111200	2.26074200	-2.59976600
C	-2.53446300	-0.14484500	-2.03614100

O	0.43006700	1.83968800	-1.53839400
O	-1.24098200	2.36651900	-0.21407900
O	-1.56292300	-0.72241500	-2.51727500
O	-3.32182400	-0.69150700	-1.15092100
H	-0.84382100	1.17859700	-4.04170100
H	-2.28016800	2.43626800	-0.18754400
H	-3.06898100	-1.89596100	-0.87961100
O	0.21839600	-0.16538800	-4.49648500
N	2.41577100	-1.09914000	-2.05561100
H	-0.25842000	-0.71367400	-3.79988000
C	0.59771900	-4.88781500	-3.54634000
C	0.52862200	-6.00219500	-2.48575900
C	0.46176900	-4.36670700	-0.50723700
O	-0.12091400	-5.25883200	-4.64156100
O	1.19173400	-3.83084500	-3.52625300
O	1.68533300	-4.37262800	-0.40436900
O	-0.27796300	-3.40415500	-0.04253900
H	-0.14266600	-4.56226900	-5.35730100
H	-1.47515600	-3.47563800	-0.25487200
Cl	-3.03638500	-3.24433800	-0.19207200
C	1.51243000	0.24658500	-4.04376100
H	1.95028900	0.70747200	-4.95314200
H	1.38789200	1.03424600	-3.26512400
C	2.34532000	-0.95119500	-3.57446000
H	1.90378400	-1.89236700	-4.00897900
H	3.37813400	-0.88603400	-3.97971300

C	1.03142400	-1.26952000	-1.48066500
H	0.46035500	-2.05970200	-2.01134100
H	1.07059500	-1.58316500	-0.41301000
H	0.43700800	-0.33886900	-1.54043400
C	3.22938100	-2.33054100	-1.72085200
H	3.19346900	-2.53717500	-0.62451700
H	2.78661100	-3.23656800	-2.19334800
H	4.27640900	-2.23547200	-2.03099100
C	3.07497700	0.11567700	-1.44811600
H	3.19019100	-0.00775000	-0.34833700
H	4.07072900	0.31031300	-1.87225600
H	2.45729100	1.03193300	-1.59571700
H	0.06361400	-6.91724700	-2.93701800
H	-2.15253500	3.31904000	-2.60963200
C	-0.25481500	-5.53156000	-1.23608000
H	-0.38702600	-6.38284400	-0.53097100
O	-1.49815800	-4.98891400	-1.66250800
H	-2.24868800	-5.60012400	-1.47275800
O	-3.70121400	1.87604700	-1.25880100
H	-4.20597500	1.07411200	-0.77258500
C	-2.94587800	1.28958000	-2.37307900
H	-3.61543500	1.28182700	-3.26396100
O	1.83633100	-6.40455300	-2.15532200
H	2.28298000	-5.72167100	-1.54463800
I	3.08227700	3.27552800	0.41902700
I	5.10310300	3.75542400	-1.14119400

I	-4.67699300	3.57827700	-0.26805100
H	-4.89309100	3.88832000	-1.87732300
I	-5.74309200	-0.03530600	0.96517000
H	-4.77362200	-1.36590900	0.77276600

Transition State 3 (TS3)

Imaginary frequency: -1580.04 cm^{-1}

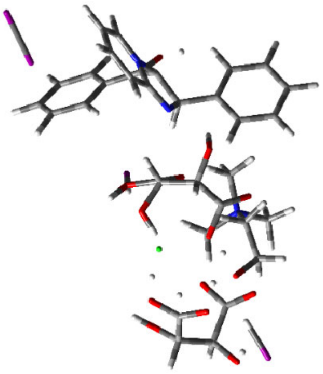

	(Hartree/Particle)
Zero-point correction=	0.677963
Thermal correction to Energy=	0.743678
Thermal correction to Enthalpy=	0.744622
Thermal correction to Gibbs Free Energy=	0.560144
Sum of electronic and zero-point Energies=	-0.197226
Sum of electronic and thermal Energies=	-0.131511
Sum of electronic and thermal Enthalpies=	-0.130567
Sum of electronic and thermal Free Energies=	-0.315045

Charge: 0 Multiplicity: 1

C	0.76492600	-4.39868900	2.63600300
C	0.26205800	-4.64389200	3.91923800
C	-0.90597000	-4.00261400	4.34095700
C	-1.56065600	-3.11782300	3.48024600
C	-1.06137700	-2.85619400	2.19979600
C	0.11140500	-3.50741900	1.78365800
C	-3.13913200	-1.42405800	1.66242200
C	-1.72376500	-1.86134400	1.26130800
C	-3.59475000	1.38070800	0.34059100
C	-3.72910800	1.16355600	1.72911400
C	-3.38455100	-0.13188500	2.28487200
C	-3.98719400	2.61369000	-0.21367900
C	-4.43202800	3.65129500	0.63925400
C	-4.52824200	3.44290100	2.01491300
C	-4.18397500	2.20224900	2.56173300
O	-3.33133800	-0.35456200	3.55202000

H	1.66064100	-4.91600800	2.28835100
H	0.77003600	-5.34344700	4.58034000
H	-1.31038900	-4.19892300	5.33271900
H	-2.50275500	-2.63556600	3.82075300
H	0.50632100	-3.33049800	0.77341200
H	-3.16711100	0.59491100	-0.29819200
H	-3.74972800	2.84408300	-1.26272000
H	-4.69626100	4.62193700	0.20852900
H	-4.87444000	4.24653900	2.66728000
H	-4.26431800	2.02822600	3.64072500
N	-1.87940300	-2.48184400	-0.09824100
C	-3.16877900	-2.54468900	-0.39799400
C	-3.84975900	-3.08841500	-1.53839200
C	-5.37161400	-1.89252200	0.54444400
C	-5.21578700	-3.02672400	-1.60586900
H	-3.24435500	-3.56037100	-2.30935000
C	-5.98779300	-2.42917800	-0.56520200
H	-5.92647100	-1.42906400	1.36547900
H	-5.74604500	-3.43980600	-2.46746200
H	-7.07138700	-2.38801300	-0.66233100
N	-3.99642500	-1.93066600	0.62185800
H	-1.04379800	-0.96982100	1.16552700
H	-3.14442900	-1.67291200	3.30189900
C	6.27460900	2.51287300	1.68178000
O	8.21331100	2.39178000	0.21666800
C	7.11891600	3.18055400	0.58182800

C	5.33791100	2.34171000	-1.09609200
O	6.46113100	1.45487700	2.23139000
O	5.14707400	3.20702700	2.04606300
O	5.72372900	1.16819900	-0.94648700
O	4.20072900	2.69657100	-1.54396300
H	7.91776000	1.49892300	-0.18302000
H	4.97621200	4.05209600	1.48871200
H	3.33083500	1.64158200	-1.88413400
O	5.03881900	-1.22269100	-0.98664500
N	3.31684100	-0.43645000	2.06938100
H	4.82105400	-0.23364600	-1.21682000
C	2.22977200	-3.20365200	-1.49481400
C	0.88898100	-3.42288500	-2.21015300
C	0.24101800	-1.04725400	-1.34387100
O	2.98324100	-2.36118500	-2.24037500
O	2.60918700	-3.64387200	-0.43401700
O	0.69921600	-1.12114500	-0.22955100
O	-0.28810700	0.17681600	-1.71449700
H	3.89153800	-2.07155000	-1.78777000
H	-0.31137300	0.30340300	-2.71731800
Cl	2.51127000	0.49017300	-1.89248000
C	5.06561600	-1.40787200	0.43806400
H	5.57836400	-2.38524700	0.55326300
H	5.69200800	-0.60378400	0.88136300
C	3.63544700	-1.46162800	0.98693000
H	2.90519500	-1.31079800	0.13964600

H	3.40349800	-2.49157500	1.35720000
C	3.32567600	0.95043800	1.47478300
H	2.44265000	1.11947000	0.82318600
H	3.33741800	1.74198300	2.24824100
H	4.22285000	1.11173000	0.84526000
C	1.93394700	-0.73139000	2.61192300
H	1.61124900	0.03276100	3.33750100
H	1.17777300	-0.74045200	1.78965700
H	1.88271200	-1.72168800	3.09765700
C	4.31815700	-0.51784300	3.19769600
H	4.00298000	0.08605500	4.06601000
H	4.47220300	-1.55467500	3.53380400
H	5.31094700	-0.10266900	2.89282900
H	1.07763600	-3.94121800	-3.19194000
H	7.58467500	4.12039700	0.98490400
C	0.15477200	-2.08780900	-2.46674400
H	-0.93049400	-2.30914100	-2.67758100
O	0.62003800	-1.43552300	-3.63733100
H	1.62660100	-1.35554500	-3.61752300
O	5.37646700	4.58207100	-0.18647700
H	4.55565600	4.61504400	-0.76768600
C	6.22793300	3.51301500	-0.63909200
H	6.85302300	3.88511200	-1.48523300
O	0.08294700	-4.32744000	-1.50645700
H	-0.28937400	-3.91387700	-0.65762900
I	-0.31011700	2.21564300	0.94973700

H	0.12944900	1.41039800	-0.46914200
I	8.42558700	-0.65875600	-1.52541800
H	6.74856300	-0.79910800	-1.54472700
I	-6.23023600	1.53495300	-0.73997800
I	-8.52084400	0.50257300	-1.39609800

Intermediary 6 (Int6)

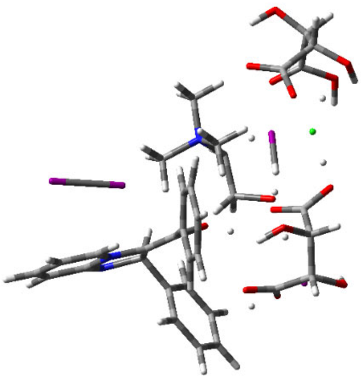

	(Hartree/Particle)
Zero-point correction=	0.681921
Thermal correction to Energy=	0.746762
Thermal correction to Enthalpy=	0.747706
Thermal correction to Gibbs Free Energy=	0.570899
Sum of electronic and zero-point Energies=	-0.284714
Sum of electronic and thermal Energies=	-0.219873
Sum of electronic and thermal Enthalpies=	-0.218928
Sum of electronic and thermal Free Energies=	-0.395735

Charge: 0 Multiplicity: 1

C	-0.19475000	5.82870100	0.04730200
C	1.04461700	6.17110500	0.59692700
C	1.52222200	5.48489400	1.71844800
C	0.76982900	4.45142200	2.28339000
C	-0.46326800	4.09302700	1.71887800
C	-0.94908500	4.79254000	0.60545200
C	-1.95734800	2.08429200	1.25881100
C	-1.24919700	2.94751900	2.32327300
C	-1.92562500	-0.82751800	-0.75187700
C	-1.89625900	0.57151400	-0.79467700
C	-1.32916500	1.33891400	0.32809700
C	-2.46427200	-1.55748800	-1.83473500
C	-2.91778300	-0.86488500	-2.97950600
C	-2.87087100	0.53130100	-3.02037000
C	-2.36492900	1.25101600	-1.93472900
O	0.04612500	1.28656000	0.46473100
H	-0.57974700	6.37586700	-0.81271600

H	1.63558600	6.97389100	0.15775500
H	2.48349500	5.75942700	2.15213500
H	1.13234600	3.94555600	3.17433700
H	-1.91731100	4.53423400	0.17740300
H	-1.51889100	-1.36163800	0.11053100
H	-2.32376600	-2.64930200	-1.87457900
H	-3.28968500	-1.42738800	-3.84073000
H	-3.22203700	1.06366600	-3.90660900
H	-2.29868000	2.34652800	-1.97815800
N	-2.34061500	3.45949900	3.20654000
C	-3.49656500	3.05462500	2.73099300
C	-4.84524800	3.28224000	3.19600500
C	-4.45163000	1.65414200	0.88871200
C	-5.89722100	2.72016900	2.54214200
H	-4.95710900	3.91700700	4.07495800
C	-5.70471100	1.89487500	1.37967900
H	-4.26545000	1.03225100	0.00435000
H	-6.92146200	2.88269300	2.88537400
H	-6.57669500	1.45095400	0.89940400
N	-3.35372600	2.21170300	1.53919800
H	-0.57450500	2.29070300	2.93892800
H	1.23364300	4.11111100	-1.02331500
C	1.09621200	2.84014300	-2.55245100
O	3.15227300	2.59863300	-3.89280600
C	1.85978400	2.10733700	-3.65917900
C	2.53541000	0.20900400	-2.03487100

O	1.86659400	3.57869500	-1.68413000
O	-0.10796200	2.70246900	-2.39549200
O	2.02257000	0.53641600	-0.93537200
O	3.63501000	-0.43768300	-2.11626400
H	3.63731700	2.80042700	-3.02564400
H	0.51454800	1.24645000	-0.45557600
H	4.08394300	-0.92285700	-1.00984700
O	3.48940100	1.15651100	1.08121200
N	1.44403300	-0.90686000	2.91988800
H	3.03399800	0.53324300	0.36143100
C	5.40972000	-3.13792500	2.35790800
C	5.26771800	-4.61625100	1.96570400
C	3.17061700	-4.15943500	0.54212000
O	6.56357100	-2.65423900	1.82684800
O	4.67223600	-2.46270300	3.03964300
O	2.38257600	-4.11031000	1.46166100
O	2.80394800	-3.67748700	-0.68825900
H	6.66126400	-1.65730500	1.92002900
H	3.62683400	-3.63865600	-1.34710600
Cl	4.49565300	-1.57684100	0.25984100
C	2.61778500	1.28061500	2.22362100
H	3.05858600	2.11925500	2.79945700
H	1.59777300	1.56093700	1.88133600
C	2.68822900	-0.02706300	3.01451200
H	3.58021600	-0.62091300	2.63755300
H	2.91026400	0.17513100	4.08525000

C	1.17053100	-1.31436300	1.49586800
H	0.97995800	-0.44051700	0.82824800
H	2.03390200	-1.85927000	1.05323100
H	0.30823700	-1.99539800	1.41869300
C	1.68855500	-2.15598400	3.74230900
H	2.59568800	-2.69458800	3.36785900
H	1.85290400	-1.92885900	4.80590000
H	0.85107700	-2.86923000	3.65571600
C	0.24492700	-0.17293900	3.47595100
H	-0.66210300	-0.80264700	3.45215900
H	0.40463500	0.14668700	4.51846900
H	0.01043800	0.73837600	2.87116500
H	6.27873700	-5.10879100	1.95472300
H	1.33277500	2.28414400	-4.63784900
C	4.56675900	-4.80039400	0.60031400
H	4.45177700	-5.90417400	0.39902900
O	5.31019000	-4.33939600	-0.50992100
H	5.82490700	-3.50183800	-0.29645100
O	0.59173900	0.01605500	-3.45479300
H	-0.06588700	0.57890200	-2.95141000
C	1.89665700	0.57923900	-3.38611000
H	2.45754400	0.06294000	-4.21102900
O	4.56381700	-5.30870000	2.97310600
H	3.62282400	-4.96986800	3.04165900
I	1.11180100	-2.92003700	-1.75034300
H	0.47304400	-1.99640500	-2.97391200

I	3.80179800	3.83576300	-0.70471400
H	3.57552600	2.36678500	0.29167400
I	-4.75630700	-1.72468900	-0.80545100
I	-7.08391500	-1.94153800	0.32580600

Intermediary 7 (Int7)

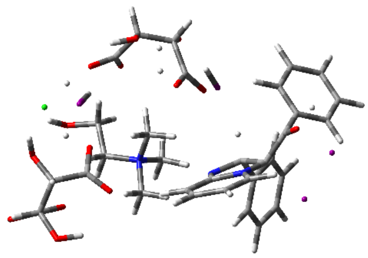

	(Hartree/Particle)
Zero-point correction=	0.677127
Thermal correction to Energy=	0.744153
Thermal correction to Enthalpy=	0.745097
Thermal correction to Gibbs Free Energy=	0.556609
Sum of electronic and zero-point Energies=	-0.229711
Sum of electronic and thermal Energies=	-0.162685
Sum of electronic and thermal Enthalpies=	-0.161741
Sum of electronic and thermal Free Energies=	-0.350229

Charge: 0 Multiplicity: 1

C	3.95932500	-3.13262200	2.77527100
C	3.81169400	-2.75493800	4.11331800
C	3.13794900	-1.57171400	4.43288600
C	2.61408700	-0.76564900	3.41908900
C	2.76327800	-1.14250700	2.07529900
C	3.43519500	-2.33221800	1.75685100
C	3.12803200	0.86098400	0.43754700
C	2.18544600	-0.30924900	0.96033100
C	5.57765200	-1.22468500	-0.76201000
C	4.30523800	-0.78692600	-1.16259500
C	3.84068200	0.57019200	-0.82428100
C	6.01997900	-2.49504100	-1.13823400
C	5.20238600	-3.32365800	-1.91352400
C	3.93715100	-2.88268000	-2.31720500
C	3.48471000	-1.61543100	-1.94450500
O	4.30714900	1.58649600	-1.55867200
H	4.48651000	-4.05108900	2.52347900

H	4.22425200	-3.37784100	4.90468200
H	3.02932600	-1.27170800	5.47354400
H	2.09704500	0.16072700	3.67260500
H	3.55861400	-2.63629900	0.71509400
H	6.21889200	-0.57585000	-0.15480300
H	7.00560900	-2.83901800	-0.82365200
H	5.54998900	-4.31566700	-2.20341600
H	3.30085300	-3.53321100	-2.91920700
H	2.49057700	-1.27397700	-2.25002900
N	0.91833600	0.35146500	1.37180000
C	0.95048100	1.62251800	1.03160300
C	-0.05659000	2.64922200	1.14849100
C	2.50039400	3.33411400	0.08975800
C	0.22555700	3.92126900	0.75969000
H	-1.03803300	2.35189400	1.53450900
C	1.52010500	4.27158000	0.23579300
H	3.51675200	3.56056500	-0.30034500
H	-0.52654100	4.71007800	0.83625700
H	1.70786200	5.30635400	-0.04627700
N	2.22372000	2.00618500	0.43838700
H	1.92897300	-0.97612700	0.07877500
H	0.43264500	-3.10151000	-1.77119200
I	4.88709200	1.23151600	1.80019500
C	-0.46088400	-0.67122700	-2.62783400
O	-0.83169300	-2.96722900	-3.14880200
C	-1.07020500	-1.66019500	-3.64366400

C	-3.44110400	-1.67775400	-2.68121100
O	0.35631400	-1.01299800	-1.79683900
O	-0.82324900	0.62344000	-2.68668800
O	-3.15471900	-2.58810300	-1.89784100
O	-4.43053500	-0.86931500	-2.56381800
H	-1.59491300	-3.27110100	-2.52894300
H	-1.55250400	0.84131800	-3.38701700
H	-5.34895200	-1.19984600	-1.54899600
O	-5.15622300	-3.76961000	1.57433700
N	-2.07994700	-1.62648600	1.92255100
H	-5.88338600	-3.23486700	1.17821700
C	-6.03999700	0.96603100	3.43887900
C	-6.10505800	2.37197200	2.81113700
C	-4.26410800	1.73908300	1.16120700
O	-7.23765000	0.67045800	4.00879900
O	-5.11327800	0.18685700	3.47935800
O	-3.26149200	2.22795200	1.64506000
O	-4.21564300	0.60066200	0.47647600
H	-7.26047700	-0.23303700	4.43736300
H	-5.23491600	0.14809300	0.23270100
Cl	-6.20747800	-1.08566000	-0.43297200
C	-3.88732900	-3.28418500	1.13962300
H	-3.21669000	-4.15349200	1.27710100
H	-3.92127400	-3.00191000	0.06507300
C	-3.52141400	-2.09929500	2.05353300
H	-4.20351100	-1.23254500	1.84077200

H	-3.73744100	-2.37802300	3.11139400
C	-1.79917900	-1.20021000	0.50406600
H	-2.42658300	-0.32838900	0.21815800
H	-0.74016500	-0.88425600	0.37196000
H	-1.99582600	-2.01528600	-0.21599000
C	-1.87926300	-0.45122900	2.85485200
H	-0.83470400	-0.07504700	2.79540300
H	-2.53380300	0.40150200	2.57377400
H	-2.10010700	-0.71333400	3.89945900
C	-1.11217600	-2.72070200	2.31310700
H	-0.06658000	-2.33997200	2.29847700
H	-1.31846300	-3.10783500	3.32226500
H	-1.14388800	-3.56757800	1.60057500
H	-7.14231400	2.79050900	2.91911100
H	-0.47541700	-1.60866000	-4.59381900
C	-5.66920300	2.35090700	1.32803100
H	-5.68938000	3.38581300	0.91086600
O	-6.57807800	1.48660900	0.64982800
H	-6.77719300	1.83324300	-0.26628700
O	-2.67537200	-0.06197400	-4.35590200
H	-3.62238100	0.25675600	-4.22850400
C	-2.56502000	-1.42570400	-3.92603600
H	-2.91259500	-2.08035700	-4.76419500
O	-5.29734200	3.25196100	3.56114700
H	-4.32205600	3.07500900	3.40386800
I	-4.82872900	2.48128900	-2.35688100

I	0.98094400	-4.11353600	-0.53027100
I	5.83370800	3.04415000	-1.27031100
H	-4.46534900	0.86389400	-2.09044000
H	4.88769900	1.22696700	-2.42295200

Transition State 4 (TS4)

Imaginary frequency: -299.63 cm^{-1}

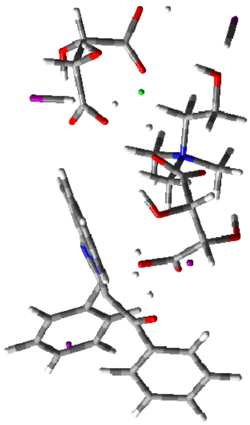

	(Hartree/Particle)
Zero-point correction=	0.671329
Thermal correction to Energy=	0.737594
Thermal correction to Enthalpy=	0.738539
Thermal correction to Gibbs Free Energy=	0.553909
Sum of electronic and zero-point Energies=	-0.206631
Sum of electronic and thermal Energies=	-0.140366
Sum of electronic and thermal Enthalpies=	-0.139422
Sum of electronic and thermal Free Energies=	-0.324051

Charge: 0 Multiplicity: 1

C	6.20826400	-2.73077200	3.27267800
C	6.76577300	-1.80943600	4.16530900
C	6.26383500	-0.50573300	4.22769900
C	5.20989900	-0.11728100	3.39626800
C	4.66669400	-1.03554400	2.48415800
C	5.15849800	-2.34922600	2.43348300
C	3.71318600	0.38783000	0.49299400
C	3.53639400	-0.63470400	1.60184700
C	6.58396700	-1.11534600	0.07726500
C	5.53302800	-0.96707600	-0.84518800
C	4.47871700	0.05672200	-0.74513100
C	7.51871200	-2.13722400	-0.08873700
C	7.42375700	-3.01254800	-1.17628600
C	6.39400000	-2.85990000	-2.11096400
C	5.45423700	-1.84251300	-1.95553500
O	4.09107500	0.44373000	-1.93620600

H	6.58670200	-3.75096500	3.23467800
H	7.58355500	-2.10965600	4.81801900
H	6.68843100	0.20892600	4.93119300
H	4.80338800	0.89318900	3.46508800
H	4.72280400	-3.07544100	1.74264800
H	6.68713700	-0.42562600	0.92656900
H	8.32534000	-2.25084900	0.63583700
H	8.15481700	-3.81338200	-1.29713700
H	6.31652700	-3.54252800	-2.95761200
H	4.64458500	-1.72659900	-2.68644700
N	2.32961500	-0.26906800	2.33379000
C	1.70831900	0.67796900	1.67242600
C	0.45322000	1.35837500	1.90548400
C	2.05983100	2.16739800	-0.31870900
C	0.05559300	2.35464000	1.08002300
H	-0.13860900	1.01809200	2.76881600
C	0.87645400	2.77263500	-0.03880600
H	2.71760100	2.47525900	-1.14430400
H	-0.89938500	2.86899400	1.23279300
H	0.52473300	3.60173800	-0.65490500
N	2.47144900	1.07244600	0.47484900
H	3.15832300	-1.70585100	0.91799500
H	3.15289400	0.94594500	-2.03690500
I	5.47762100	2.02161500	0.62170500
C	1.44238500	1.81514400	-3.69398000
O	0.35562100	0.44925300	-5.38161600

C	0.56512500	1.76760000	-4.95506000
C	-1.63470700	1.82490800	-3.63469800
O	1.96804100	0.86138300	-3.15014000
O	1.67252900	3.03590600	-3.14580500
O	-1.67324800	0.60113200	-3.51263900
O	-2.25769100	2.68390000	-2.90190900
H	-0.21819300	-0.05728100	-4.71897300
H	1.10136800	3.79870900	-3.55398800
H	-3.14574400	2.18328100	-2.08521300
O	-4.89400200	-1.40795500	-2.05904000
N	-1.52061400	-1.95407900	-0.41891500
H	-5.17577200	-0.49170400	-1.79366900
C	-5.17543300	-1.35045700	2.41565800
C	-5.15274700	-0.11123500	3.32969800
C	-3.01699400	0.80176700	2.24812800
O	-6.44008500	-1.78515400	2.25485400
O	-4.23304500	-1.89907900	1.87979700
O	-2.19579600	0.38160600	3.04070600
O	-2.66730100	1.01696200	0.97814200
H	-6.51717300	-2.59513100	1.61360400
H	-3.48642400	1.36692500	0.31480200
Cl	-4.25878700	1.80433700	-1.20126500
C	-3.46012300	-1.52174200	-2.04830800
H	-3.27416000	-2.41264200	-2.67722500
H	-3.01916300	-0.61930300	-2.53032800
C	-3.01128900	-1.70210400	-0.59231700

H	-3.29589800	-0.79533900	0.00527600
H	-3.58560200	-2.53902000	-0.11427600
C	-0.72260100	-0.76811200	-0.90058400
H	-0.95576100	0.13704200	-0.30363200
H	0.37375600	-0.96884700	-0.81838200
H	-0.92865000	-0.52984700	-1.96276300
C	-1.23057900	-2.16247000	1.05417800
H	-0.17424700	-2.46144900	1.21503700
H	-1.39939000	-1.23154800	1.63851000
H	-1.88643100	-2.93319900	1.49636100
C	-1.11051000	-3.19198700	-1.17906400
H	-0.02030400	-3.42317600	-1.00031300
H	-1.68882600	-4.07468100	-0.86637800
H	-1.21780700	-3.06493900	-2.26742500
H	-6.19917700	0.14553900	3.64652600
H	1.13188100	2.23776500	-5.80585700
C	-4.47986700	1.09278200	2.62503600
H	-4.52162200	1.98664600	3.29356800
O	-5.16310100	1.31432500	1.39354300
H	-5.52608800	2.23833500	1.34993500
O	-0.44444000	3.84182300	-4.34908300
H	-1.16882300	4.20654800	-3.75071800
C	-0.77743100	2.49589700	-4.72453600
H	-1.35734100	2.54549300	-5.68032600
O	-4.49770500	-0.41703200	4.53827700
H	-3.51069500	-0.55597700	4.39562100

I	2.45951000	-2.79047800	-0.65570700
I	-3.63732500	4.58636700	1.55851200
H	-3.87056200	4.28154800	-0.04811600
I	-6.50815800	-3.90886300	-0.50381400
H	-5.76553800	-2.61769700	-1.32904400

Intermediary 8 (Int8)

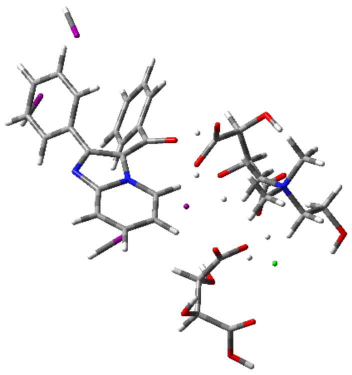

	(Hartree/Particle)
Zero-point correction=	0.671438
Thermal correction to Energy=	0.739284
Thermal correction to Enthalpy=	0.740229
Thermal correction to Gibbs Free Energy=	0.547792
Sum of electronic and zero-point Energies=	-0.281553
Sum of electronic and thermal Energies=	-0.213707
Sum of electronic and thermal Enthalpies=	-0.212762
Sum of electronic and thermal Free Energies=	-0.405199

Charge: 0 Multiplicity: 1

C	-5.51318000	1.31583900	-2.36317700
C	-6.23060700	1.87932000	-1.30206800
C	-5.58376500	2.70720800	-0.37911500
C	-4.22222900	2.98675500	-0.52063800
C	-3.50318000	2.41779000	-1.58360100
C	-4.14874300	1.57821300	-2.50540400
C	-0.98317400	1.81251700	-1.86099500
C	-2.08034600	2.73762300	-1.75289800
C	-2.59175900	-1.47038600	-1.49372200
C	-1.98001300	-0.32458900	-0.95751500
C	-0.93516500	0.39041000	-1.72957600
C	-3.51933200	-2.17924400	-0.73145500
C	-3.83449200	-1.75160600	0.56589100
C	-3.23276400	-0.60725800	1.09434000
C	-2.30485000	0.11253700	0.33510000
O	0.00330700	-0.25694800	-2.27719200
H	-6.02133700	0.68104900	-3.08840000

H	-7.29765200	1.67961700	-1.19660800
H	-6.14370500	3.13847300	0.45036300
H	-3.71714200	3.65008500	0.18263400
H	-3.59030300	1.14511200	-3.33344600
H	-2.33822800	-1.80494800	-2.50039400
H	-3.99596400	-3.06920800	-1.14214500
H	-4.55076900	-2.31603500	1.16707600
H	-3.48822500	-0.27008800	2.10001800
H	-1.81972600	1.00352600	0.75271100
N	-1.65267500	4.02885100	-1.87850500
C	-0.27594300	3.98242000	-2.05645500
C	0.68205800	5.01403000	-2.19791000
C	1.49915500	2.28706700	-2.17890100
C	2.01114500	4.67033100	-2.32086600
H	0.33465400	6.04870300	-2.19057200
C	2.41689900	3.30867200	-2.30612900
H	1.76389300	1.21786800	-2.13161000
H	2.77591200	5.44393600	-2.42018000
H	3.48611500	3.06327200	-2.36968400
N	0.15926300	2.62709900	-2.07985800
H	0.16048300	-1.54297200	-1.85349500
C	0.26188700	-2.88763400	-0.28027400
O	0.82637800	-5.23640500	-0.66632300
C	0.33865900	-4.31206300	0.27636400
C	2.66472700	-3.87002100	1.25791100
O	0.27141300	-2.70364400	-1.54759400

O	0.36808500	-1.81168300	0.50591600
O	3.20826800	-4.16903600	0.18825900
O	3.14094200	-3.16745900	2.20828000
H	1.81262100	-5.07788100	-0.83379300
H	0.33025300	-2.08075800	1.60445800
H	4.32691800	-2.52064500	2.02055900
O	7.21629800	-3.74609400	-0.42429500
N	4.77782400	-2.00121200	-2.70837700
H	7.23325100	-3.24701800	0.42994100
C	6.96152800	2.37375200	1.53071600
C	5.55576000	2.98726600	1.43408900
C	4.76223200	1.21024500	-0.24154300
O	7.43802300	2.53892100	2.79994300
O	7.62825500	1.84672100	0.67398100
O	4.77408000	1.75158700	-1.33995600
O	4.98089900	-0.09049900	-0.14903400
H	8.34142000	2.13691200	2.93152500
H	5.04636100	-0.50421700	0.94467600
Cl	5.53758000	-1.76308400	1.94090100
C	5.94735700	-3.60323200	-1.05587700
H	5.88784800	-4.48020500	-1.72541100
H	5.12476100	-3.64519000	-0.30621200
C	5.98641400	-2.26550100	-1.82098000
H	6.07760900	-1.42455100	-1.07262400
H	6.92042300	-2.22220500	-2.42471700
C	3.50392800	-1.98601500	-1.90489100

H	3.53875100	-1.20360900	-1.09929300
H	2.61660100	-1.77471700	-2.52063600
H	3.33308500	-2.94768900	-1.37456500
C	4.95698700	-0.65195200	-3.37095000
H	4.10750900	-0.39940100	-4.02366000
H	5.01552100	0.16651300	-2.59887200
H	5.88366900	-0.59960000	-3.96113600
C	4.67832700	-3.06639400	-3.78051600
H	3.85185400	-2.85608000	-4.48123900
H	5.61102400	-3.14082900	-4.36465600
H	4.48209400	-4.06068600	-3.33642900
H	5.29606300	3.51096200	2.39278500
H	-0.70226600	-4.66981300	0.49701300
C	4.48344700	1.93570600	1.08300800
H	3.47344800	2.43283100	1.03942200
O	4.52683700	0.93448300	2.09694000
H	3.58513000	0.60708200	2.29316400
O	0.61460300	-3.41196900	2.48298500
H	1.34719200	-3.04833000	3.09264500
C	1.21737200	-4.33314200	1.54916200
H	1.21204400	-5.34447900	2.01655800
O	5.57424800	4.02186200	0.46846200
H	5.68671100	3.64606900	-0.45198100
I	0.64077800	2.55693700	1.68182400
I	1.58010400	-0.05528200	0.68121800
I	-7.05122000	-1.56603200	-0.85775200

I	-6.93342600	0.06695100	2.08979500
H	-8.46754700	-2.32530800	-0.50320900
H	-6.98387900	0.76789500	3.57956800
H	0.30009300	4.09870900	2.19241600

Product (P)

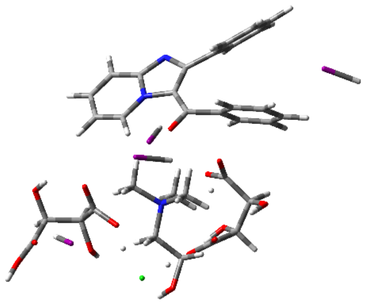

	(Hartree/Particle)
Zero-point correction=	0.668626
Thermal correction to Energy=	0.738228
Thermal correction to Enthalpy=	0.739172
Thermal correction to Gibbs Free Energy=	0.539743
Sum of electronic and zero-point Energies=	-0.278507
Sum of electronic and thermal Energies=	-0.208907
Sum of electronic and thermal Enthalpies=	-0.207963
Sum of electronic and thermal Free Energies=	-0.407392

Charge: 0 Multiplicity: 1

C	-6.63625500	2.04366800	1.57666600
C	-7.44149800	0.91661100	1.77528100
C	-6.87641400	-0.27288800	2.24690900
C	-5.50959100	-0.33632900	2.53104400
C	-4.70269000	0.79704400	2.33806500
C	-5.27003800	1.99010400	1.86058100
C	-2.14955800	1.21698000	1.95132400
C	-3.28073300	0.74705600	2.69962700
C	-3.64256300	2.74119500	-1.00002100
C	-3.11948200	1.60826300	-0.35900200
C	-2.01334800	1.75972900	0.62635800
C	-4.64758200	2.58992500	-1.95735700
C	-5.11224300	1.31157600	-2.28985000
C	-4.57345200	0.18301700	-1.66457100
C	-3.58295200	0.32749700	-0.68949800
O	-0.96676900	2.32182400	0.26636800
H	-7.07710400	2.96835000	1.20688600

H	-8.51330600	0.97385300	1.58041200
H	-7.50418000	-1.14902700	2.41244200
H	-5.06804300	-1.25680900	2.91772000
H	-4.64758500	2.87290000	1.72066100
H	-3.26863400	3.73362600	-0.74946900
H	-5.06786800	3.46668300	-2.44773300
H	-5.89756900	1.19510100	-3.03729800
H	-4.92598900	-0.81463800	-1.93359600
H	-3.16517900	-0.55750500	-0.19660200
N	-2.89472700	0.24495300	3.91592600
C	-1.51832800	0.37827500	3.99152300
C	-0.59301100	0.01200200	5.00095100
C	0.31603400	1.24159400	2.60570100
C	0.74674900	0.25188100	4.79323300
H	-0.97782500	-0.46220900	5.90620300
C	1.20202300	0.86294400	3.59084800
H	0.61800900	1.72180900	1.65802100
H	1.48366200	-0.02996800	5.54861200
H	2.27591900	1.01649400	3.43409100
N	-1.03518800	1.00246700	2.80637700
H	-0.56444900	4.07951500	-0.07767700
C	-1.56079100	-3.40013200	-0.86700800
O	-2.14809500	-3.12667500	-3.20386700
C	-1.72126100	-4.06349400	-2.24594600
C	0.76129400	-3.72419900	-2.74126000
O	-1.80069700	-2.26341400	-0.55675500

O	-1.06978500	-4.22669700	0.11726500
O	0.59976600	-2.58429500	-3.17228700
O	1.85577800	-4.21227100	-2.25986000
H	-1.42346400	-2.44537100	-3.37917600
H	-0.75101200	-5.14974900	-0.21720200
H	2.89357700	-3.44423400	-2.31611600
O	3.49456400	-0.13290100	-4.67105000
N	1.47395500	1.05912400	-1.71535200
H	4.14316200	-0.78448600	-4.31151000
C	6.88092200	-1.12629800	1.71066500
C	5.76975300	-1.88991500	2.45070800
C	3.86467600	-0.98079300	0.98379100
O	8.02268500	-1.86927400	1.72012100
O	6.85759700	-0.03199200	1.19342100
O	3.56662900	-0.05334800	1.72914200
O	3.56386200	-0.96540600	-0.28637000
H	8.78555900	-1.42357700	1.25086900
H	3.99029800	-1.88838600	-0.97094500
Cl	4.17629900	-2.70243800	-2.30989100
C	2.32399100	-0.09703200	-3.85791600
H	1.57364800	0.38820800	-4.50930200
H	1.99498100	-1.12861400	-3.60588700
C	2.66268900	0.73851900	-2.61014100
H	3.43869100	0.19775400	-2.00094000
H	3.15959100	1.68467100	-2.93228200
C	0.80182900	-0.20434200	-1.23418000

H	1.51078100	-0.83063000	-0.64673800
H	-0.06111700	0.01548700	-0.57932200
H	0.43593600	-0.82732800	-2.07394600
C	1.98178400	1.82679800	-0.51424200
H	1.15358800	2.06707300	0.18955700
H	2.73230900	1.22744000	0.05528600
H	2.46659900	2.77624300	-0.80230800
C	0.46487900	1.90601600	-2.45654100
H	-0.39278100	2.16529600	-1.79277800
H	0.90682800	2.85791200	-2.80145400
H	0.05537300	1.37830400	-3.33283600
H	6.19725100	-2.81319200	2.92369900
H	-2.55637900	-4.81207100	-2.20742000
C	4.59900100	-2.23553900	1.50220800
H	3.86781800	-2.89787200	2.02977800
O	5.10948600	-2.84971700	0.32060200
H	5.03782200	-3.83104000	0.36124900
O	-0.13220300	-5.76201200	-1.72344400
H	0.86489800	-5.88913100	-1.64521800
C	-0.40123800	-4.73388100	-2.69013000
H	-0.52830300	-5.22670400	-3.68478500
O	5.32837200	-1.12204200	3.54638800
H	4.79573700	-0.31903600	3.23541900
I	0.91412600	-2.27846100	2.16053500
I	0.22041400	5.37030300	-0.78535300
I	5.71578300	2.47140400	-0.95320700

I	-7.96792700	-0.93281400	-0.91250500
H	6.00971200	1.32448300	0.22231900
H	-8.77965500	-1.73206900	-2.09427900
H	-0.17869700	-3.30413700	1.40683300

IRC Graphs of Transition State in DES

IRC Transition state 1 (TS1)

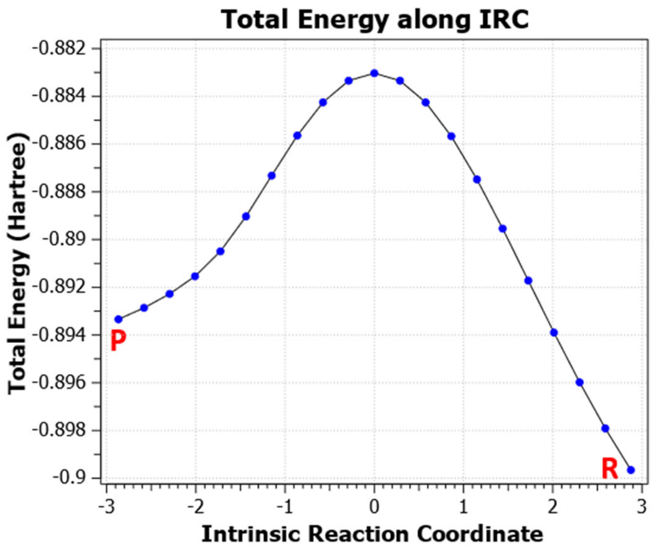

Charge: 0 Multiplicity: 1

C	6.25137123	-2.74381709	3.24976553
C	6.80888023	-1.82248109	4.14239653
C	6.30694223	-0.51877809	4.20478653
C	5.25300623	-0.13032609	3.37335553
C	4.70980123	-1.04858909	2.46124553
C	5.20160523	-2.36227109	2.41057053
C	3.75629323	0.37478491	0.47008153
C	3.57950123	-0.64774909	1.57893453
C	6.62707423	-1.12839109	0.05435253
C	5.57613523	-0.98012109	-0.86810047
C	4.52182423	0.04367691	-0.76804347
C	7.56181923	-2.15026909	-0.11164947
C	7.46686423	-3.02559309	-1.19919847
C	6.43710723	-2.87294509	-2.13387647

C	5.49734423	-1.85555809	-1.97844747
O	4.13418223	0.43068491	-1.95911847
H	6.62980923	-3.76401009	3.21176553
H	7.62666223	-2.12270109	4.79510653
H	6.73153823	0.19588091	4.90828053
H	4.84649523	0.88014391	3.44217553
H	4.76591123	-3.08848609	1.71973553
H	6.73024423	-0.43867109	0.90365653
H	8.36844723	-2.26389409	0.61292453
H	8.19792423	-3.82642709	-1.32004947
H	6.35963423	-3.55557309	-2.98052447
H	4.68769223	-1.73964409	-2.70935947
N	2.37272223	-0.28211309	2.31087753
C	1.75142623	0.66492391	1.64951353
C	0.49632723	1.34532991	1.88257153
C	2.10293823	2.15435291	-0.34162147
C	0.09870023	2.34159491	1.05711053
H	-0.09550177	1.00504691	2.74590353
C	0.91956123	2.75958991	-0.06171847
H	2.76070823	2.46221391	-1.16721647
H	-0.85627777	2.85594891	1.20988053
H	0.56784023	3.58869291	-0.67781747
N	2.51455623	1.05940091	0.45193653
H	3.20143023	-1.71889609	0.89508253
H	3.19600123	0.93289991	-2.05981747
I	5.52072823	2.00856991	0.59879253

C	1.48549223	1.80209891	-3.71689247
O	0.39872823	0.43620791	-5.40452847
C	0.60823223	1.75455491	-4.97797247
C	-1.59159977	1.81186291	-3.65761047
O	2.01114823	0.84833791	-3.17305247
O	1.71563623	3.02286091	-3.16871747
O	-1.63014077	0.58808691	-3.53555147
O	-2.21458377	2.67085491	-2.92482147
H	-0.17508577	-0.07032609	-4.74188547
H	1.14447523	3.78566391	-3.57690047
H	-3.10263677	2.17023591	-2.10812547
O	-4.85089477	-1.42100009	-2.08195247
N	-1.47750677	-1.96712409	-0.44182747
H	-5.13266477	-0.50474909	-1.81658147
C	-5.13232577	-1.36350209	2.39274553
C	-5.10963977	-0.12428009	3.30678553
C	-2.97388677	0.78872191	2.22521553
O	-6.39697777	-1.79819909	2.23194153
O	-4.18993777	-1.91212409	1.85688453
O	-2.15268877	0.36856091	3.01779353
O	-2.62419377	1.00391691	0.95522953
H	-6.47406577	-2.60817609	1.59069153
H	-3.44331677	1.35387991	0.29188953
Cl	-4.21567977	1.79129191	-1.22417747
C	-3.41701577	-1.53478709	-2.07122047
H	-3.23105277	-2.42568709	-2.70013747

H	-2.97605577	-0.63234809	-2.55324047
C	-2.96818177	-1.71514909	-0.61522947
H	-3.25279077	-0.80838409	-0.01763647
H	-3.54249477	-2.55206509	-0.13718847
C	-0.67949377	-0.78115709	-0.92349647
H	-0.91265377	0.12399691	-0.32654447
H	0.41686323	-0.98189209	-0.84129447
H	-0.88554277	-0.54289209	-1.98567547
C	-1.18747177	-2.17551509	1.03126553
H	-0.13113977	-2.47449409	1.19212453
H	-1.35628277	-1.24459309	1.61559753
H	-1.84332377	-2.94624409	1.47344853
C	-1.06740277	-3.20503209	-1.20197647
H	0.02280323	-3.43622109	-1.02322547
H	-1.64571877	-4.08772609	-0.88929047
H	-1.17469977	-3.07798409	-2.29033747
H	-6.15606977	0.13249391	3.62361353
H	1.17498823	2.22471991	-5.82876947
C	-4.43675977	1.07973691	2.60212353
H	-4.47851477	1.97360091	3.27065553
O	-5.11999377	1.30127991	1.37063053
H	-5.48298077	2.22528991	1.32702253
O	-0.40133277	3.82877791	-4.37199547
H	-1.12571577	4.19350291	-3.77363047
C	-0.73432377	2.48285191	-4.74744847
H	-1.31423377	2.53244791	-5.70323847

O	-4.45459777	-0.43007709	4.51536453
H	-3.46758777	-0.56902209	4.37270853
I	2.50261723	-2.80352309	-0.67861947
I	-3.59421777	4.57332191	1.53559953
H	-3.82745477	4.26850291	-0.07102847
I	-6.46505077	-3.92190809	-0.52672647
H	-5.72243077	-2.63074209	-1.35195647

IRC Transition state 2 (TS2)

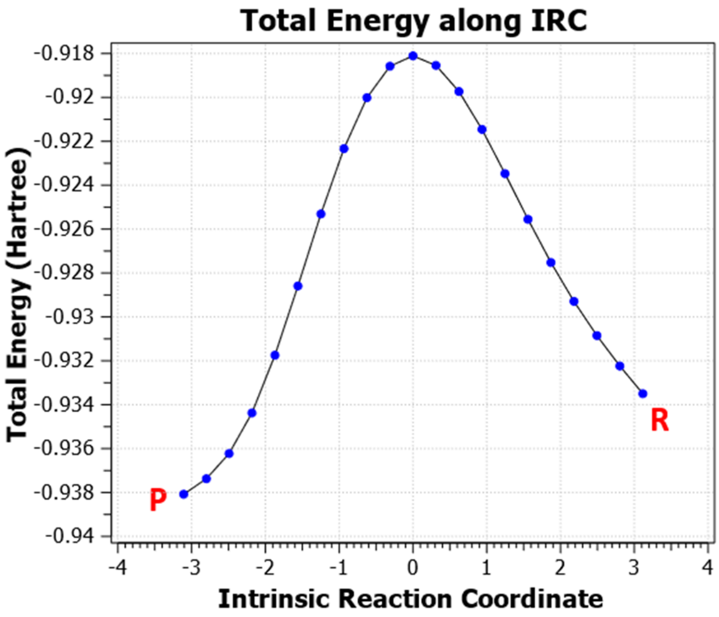

Charge: 0 Multiplicity: 1

C	7.79984958	-0.82474333	0.23185163
C	7.71008758	0.21321967	1.16418763
C	6.61212458	0.27945567	2.02818063
C	5.60796658	-0.69008633	1.96324963
C	5.69975658	-1.73695633	1.03420163
C	6.80103358	-1.80025333	0.16776063
C	3.34934458	-2.52851233	0.25279063
C	4.66584758	-2.84321633	0.98461163
C	4.64561458	0.38904667	-1.08972037
C	3.59471058	0.06646067	-0.22874737
C	2.83362458	-1.21940933	-0.31254337
C	5.24346858	1.65914267	-1.02981837
C	4.78688458	2.60777767	-0.12261437
C	3.70584558	2.30266367	0.74219463

C	3.12609358	1.01803267	0.69741063
O	1.67457158	-1.18701033	-0.70458037
H	8.65168158	-0.87786633	-0.44593137
H	8.49350958	0.96792867	1.21820363
H	6.53773858	1.08907767	2.75290763
H	4.74906358	-0.61640733	2.62888663
H	6.87904058	-2.60577233	-0.56848137
H	5.01179358	-0.35082233	-1.81992437
H	6.06850658	1.89362367	-1.70365337
H	5.25288658	3.59416067	-0.06762737
H	3.47182358	2.98003767	1.57721563
H	2.31991158	0.75694467	1.40189863
N	4.34669358	-3.33272033	2.37316663
C	3.14050058	-2.83583233	2.88645663
C	2.66261358	-3.00520433	4.20134463
C	1.11224158	-1.81966733	2.18987063
C	1.36748558	-2.57133333	4.47793163
H	3.27677258	-3.48062033	4.96081763
C	0.58938258	-1.97505233	3.47383563
H	0.54175658	-1.33974533	1.37229163
H	0.95423258	-2.69479633	5.48235463
H	-0.42581242	-1.61391233	3.68521063
N	2.38438058	-2.23131733	1.89978563
H	2.68486958	-3.40729433	0.08844663
I	4.18360758	-2.90980433	-2.07473637
H	5.12423558	-3.75453633	0.47809263

H	5.14622358	-3.40829333	3.00325063
C	-0.67475942	1.29288967	2.60560363
O	-0.52715942	3.66332067	2.05915463
C	-1.16866442	2.72492567	2.88129263
C	-3.25119542	2.41054667	1.39506463
O	0.28559358	0.98840367	1.93312963
O	-1.36869042	0.28855367	3.19002663
O	-2.65766842	2.48278267	0.33503963
O	-4.47147242	1.91830967	1.54274163
H	-0.69844542	3.47446867	1.08084263
H	-2.25494342	0.56483667	3.68255563
H	-4.95824142	1.53273967	0.59165063
O	-4.00628842	1.47197367	-3.56576737
N	-1.18864642	-0.45378533	-1.98488737
H	-4.84876742	1.24473067	-3.10876837
C	-4.41325142	-3.29460633	-3.45501937
C	-5.11969042	-4.01181333	-2.29016337
C	-4.09543842	-2.59434733	-0.38521937
O	-5.22567442	-3.33253933	-4.55277337
O	-3.32731842	-2.76519233	-3.51134737
O	-3.17014542	-3.38332433	-0.22418337
O	-4.07894242	-1.37197333	0.04433963
H	-4.83118942	-2.88723533	-5.35318037
H	-5.07710742	-0.51265033	-0.43480737
Cl	-5.73154842	0.74358067	-0.65210737
C	-2.90078142	1.34133967	-2.67330937

H	-2.10969042	1.95158667	-3.15411237
H	-3.14596242	1.76611867	-1.67707637
C	-2.54548742	-0.15586133	-2.60757837
H	-3.33655642	-0.70313633	-2.03523937
H	-2.58963842	-0.58744733	-3.63868137
C	-1.16961742	0.00950467	-0.54874837
H	-1.99697842	-0.45551133	0.02626963
H	-0.20962242	-0.24928933	-0.05087737
H	-1.27816542	1.11188167	-0.47272937
C	-0.94400042	-1.94751033	-2.02406137
H	0.09742058	-2.18922533	-1.72802037
H	-1.61755442	-2.49042833	-1.32586337
H	-1.12988442	-2.36713033	-3.02917237
C	-0.08695542	0.23593367	-2.75613737
H	0.90323858	0.03054567	-2.28830037
H	-0.04553442	-0.11152433	-3.79979737
H	-0.21685342	1.33677267	-2.76142437
H	-6.07542442	-4.46720633	-2.66359737
H	-0.85337742	3.01973667	3.91989563
C	-5.38532742	-3.03720033	-1.11864937
H	-6.07182442	-3.51373133	-0.38110437
O	-5.93140742	-1.82316033	-1.63418437
H	-6.91580142	-1.83082733	-1.60992437
O	-3.22033342	2.00611567	3.79684063
H	-4.16067242	1.68967667	3.56980763
C	-2.70587442	2.85014167	2.75945963

H	-3.02528942	3.90223967	2.96796363
O	-4.35476542	-5.12042333	-1.89002737
H	-3.54069442	-4.83316533	-1.34849137
I	-0.31153742	4.11939767	-1.80832737
I	1.78196258	3.12015467	-0.64536137
I	-4.38682542	-1.09455533	3.38547963
H	-4.00232242	-1.20462133	1.72577263

IRC Transition state 3 (TS3)

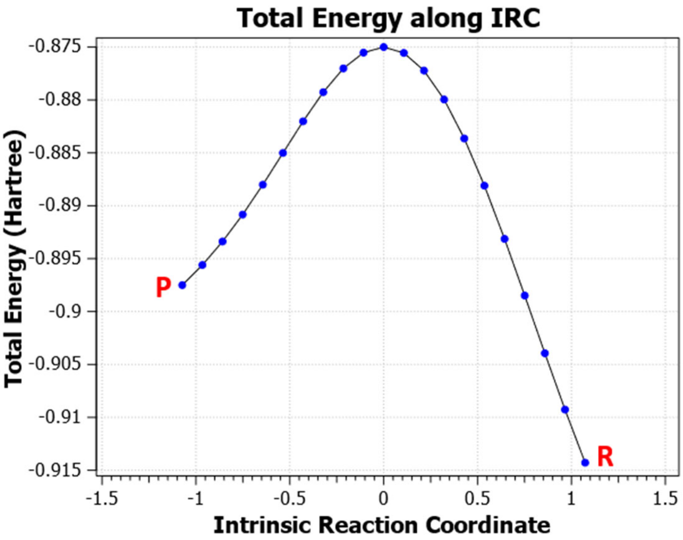

Charge: 0 Multiplicity: 1

C	0.91410873	-4.47467150	2.70505813
C	0.41124073	-4.71987450	3.98829313
C	-0.75678727	-4.07859650	4.41001213
C	-1.41147327	-3.19380550	3.54930113
C	-0.91219427	-2.93217650	2.26885113
C	0.26058773	-3.58340150	1.85271313
C	-2.98994927	-1.50004050	1.73147713
C	-1.57458227	-1.93732650	1.33036313
C	-3.44556727	1.30472550	0.40964613
C	-3.57992527	1.08757350	1.79816913
C	-3.23536827	-0.20786750	2.35392713
C	-3.83801127	2.53770750	-0.14462387
C	-4.28284527	3.57531250	0.70830913
C	-4.37905927	3.36691850	2.08396813
C	-4.03479227	2.12626650	2.63078813

O	-3.18215527	-0.43054450	3.62107513
H	1.80982373	-4.99199050	2.35740613
H	0.91921873	-5.41942950	4.64939513
H	-1.16120627	-4.27490550	5.40177413
H	-2.35357227	-2.71154850	3.88980813
H	0.65550373	-3.40648050	0.84246713
H	-3.01792827	0.51892850	-0.22913687
H	-3.60054527	2.76810050	-1.19366487
H	-4.54707827	4.54595450	0.27758413
H	-4.72525727	4.17055650	2.73633513
H	-4.11513527	1.95224350	3.70978013
N	-1.73022027	-2.55782650	-0.02918587
C	-3.01959627	-2.62067150	-0.32893887
C	-3.70057627	-3.16439750	-1.46933687
C	-5.22243127	-1.96850450	0.61349913
C	-5.06660427	-3.10270650	-1.53681387
H	-3.09517227	-3.63635350	-2.24029487
C	-5.83861027	-2.50516050	-0.49614687
H	-5.77728827	-1.50504650	1.43453413
H	-5.59686227	-3.51578850	-2.39840687
H	-6.92220427	-2.46399550	-0.59327587
N	-3.84724227	-2.00664850	0.69091313
H	-0.89461527	-1.04580350	1.23458213
H	-2.99524627	-1.74889450	3.37095413
C	6.42379173	2.43689050	1.75083513
O	8.36249373	2.31579750	0.28572313

C	7.26809873	3.10457150	0.65088313
C	5.48709373	2.26572750	-1.02703687
O	6.61031373	1.37889450	2.30044513
O	5.29625673	3.13104450	2.11511813
O	5.87291173	1.09221650	-0.87743187
O	4.34991173	2.62058850	-1.47490787
H	8.06694273	1.42294050	-0.11396487
H	5.12539473	3.97611350	1.55776713
H	3.48001773	1.56559950	-1.81507887
O	5.18800173	-1.29867350	-0.91758987
N	3.46602373	-0.51243250	2.13843613
H	4.97023673	-0.30962850	-1.14776487
C	2.37895473	-3.27963450	-1.42575887
C	1.03816373	-3.49886750	-2.14109787
C	0.39020073	-1.12323650	-1.27481587
O	3.13242373	-2.43716750	-2.17131987
O	2.75836973	-3.71985450	-0.36496187
O	0.84839873	-1.19712750	-0.16049587
O	-0.13892427	0.10083350	-1.64544187
H	4.04072073	-2.14753250	-1.71871487
H	-0.16219027	0.22742050	-2.64826287
Cl	2.66045273	0.41419050	-1.82342487
C	5.21479873	-1.48385450	0.50711913
H	5.72754673	-2.46122950	0.62231813
H	5.84119073	-0.67976650	0.95041813
C	3.78462973	-1.53761050	1.05598513

H	3.05437773	-1.38678050	0.20870113
H	3.55268073	-2.56755750	1.42625513
C	3.47485873	0.87445550	1.54383813
H	2.59183273	1.04348750	0.89224113
H	3.48660073	1.66600050	2.31729613
H	4.37203273	1.03574750	0.91431513
C	2.08312973	-0.80737250	2.68097813
H	1.76043173	-0.04322150	3.40655613
H	1.32695573	-0.81643450	1.85871213
H	2.03189473	-1.79767050	3.16671213
C	4.46733973	-0.59382550	3.26675113
H	4.15216273	0.01007250	4.13506513
H	4.62138573	-1.63065750	3.60285913
H	5.46012973	-0.17865150	2.96188413
H	1.22681873	-4.01720050	-3.12288487
H	7.73385773	4.04441450	1.05395913
C	0.30395473	-2.16379150	-2.39768887
H	-0.78131127	-2.38512350	-2.60852587
O	0.76922073	-1.51150550	-3.56827587
H	1.77578373	-1.43152750	-3.54846787
O	5.52564973	4.50608850	-0.11742187
H	4.70483873	4.53906150	-0.69863087
C	6.37711573	3.43703250	-0.57003687
H	7.00220573	3.80912950	-1.41617787
O	0.23212973	-4.40342250	-1.43740187
H	-0.14019127	-3.98985950	-0.58857387

I	-0.16093427	2.13966050	1.01879213
H	0.27863173	1.33441550	-0.40008687
I	8.57476973	-0.73473850	-1.45636287
H	6.89774573	-0.87509050	-1.47567187
I	-6.08105327	1.45897050	-0.67092287
I	-8.37166127	0.42659050	-1.32704287

IRC Transition state 4 (TS4)

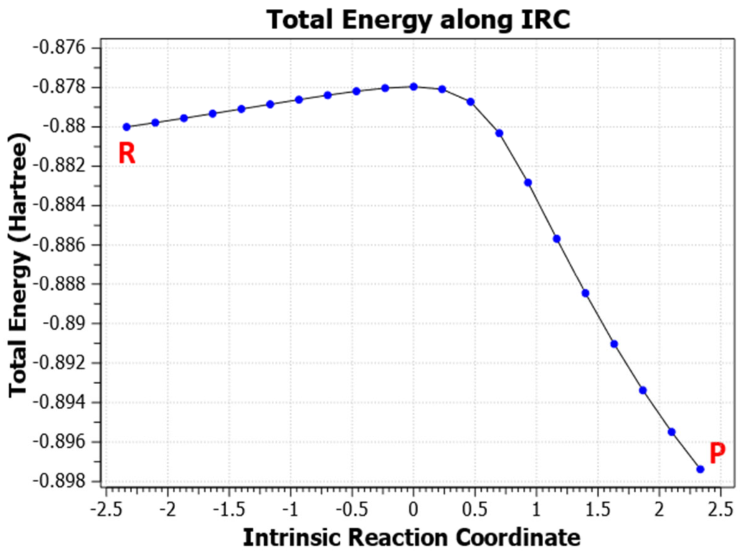

Charge: 0 Multiplicity: 1

C	6.25137123	-2.74381709	3.24976553
C	6.80888023	-1.82248109	4.14239653
C	6.30694223	-0.51877809	4.20478653
C	5.25300623	-0.13032609	3.37335553
C	4.70980123	-1.04858909	2.46124553
C	5.20160523	-2.36227109	2.41057053
C	3.75629323	0.37478491	0.47008153
C	3.57950123	-0.64774909	1.57893453
C	6.62707423	-1.12839109	0.05435253
C	5.57613523	-0.98012109	-0.86810047
C	4.52182423	0.04367691	-0.76804347
C	7.56181923	-2.15026909	-0.11164947
C	7.46686423	-3.02559309	-1.19919847
C	6.43710723	-2.87294509	-2.13387647
C	5.49734423	-1.85555809	-1.97844747

O	4.13418223	0.43068491	-1.95911847
H	6.62980923	-3.76401009	3.21176553
H	7.62666223	-2.12270109	4.79510653
H	6.73153823	0.19588091	4.90828053
H	4.84649523	0.88014391	3.44217553
H	4.76591123	-3.08848609	1.71973553
H	6.73024423	-0.43867109	0.90365653
H	8.36844723	-2.26389409	0.61292453
H	8.19792423	-3.82642709	-1.32004947
H	6.35963423	-3.55557309	-2.98052447
H	4.68769223	-1.73964409	-2.70935947
N	2.37272223	-0.28211309	2.31087753
C	1.75142623	0.66492391	1.64951353
C	0.49632723	1.34532991	1.88257153
C	2.10293823	2.15435291	-0.34162147
C	0.09870023	2.34159491	1.05711053
H	-0.09550177	1.00504691	2.74590353
C	0.91956123	2.75958991	-0.06171847
H	2.76070823	2.46221391	-1.16721647
H	-0.85627777	2.85594891	1.20988053
H	0.56784023	3.58869291	-0.67781747
N	2.51455623	1.05940091	0.45193653
H	3.20143023	-1.71889609	0.89508253
H	3.19600123	0.93289991	-2.05981747
I	5.52072823	2.00856991	0.59879253
C	1.48549223	1.80209891	-3.71689247

O	0.39872823	0.43620791	-5.40452847
C	0.60823223	1.75455491	-4.97797247
C	-1.59159977	1.81186291	-3.65761047
O	2.01114823	0.84833791	-3.17305247
O	1.71563623	3.02286091	-3.16871747
O	-1.63014077	0.58808691	-3.53555147
O	-2.21458377	2.67085491	-2.92482147
H	-0.17508577	-0.07032609	-4.74188547
H	1.14447523	3.78566391	-3.57690047
H	-3.10263677	2.17023591	-2.10812547
O	-4.85089477	-1.42100009	-2.08195247
N	-1.47750677	-1.96712409	-0.44182747
H	-5.13266477	-0.50474909	-1.81658147
C	-5.13232577	-1.36350209	2.39274553
C	-5.10963977	-0.12428009	3.30678553
C	-2.97388677	0.78872191	2.22521553
O	-6.39697777	-1.79819909	2.23194153
O	-4.18993777	-1.91212409	1.85688453
O	-2.15268877	0.36856091	3.01779353
O	-2.62419377	1.00391691	0.95522953
H	-6.47406577	-2.60817609	1.59069153
H	-3.44331677	1.35387991	0.29188953
Cl	-4.21567977	1.79129191	-1.22417747
C	-3.41701577	-1.53478709	-2.07122047
H	-3.23105277	-2.42568709	-2.70013747
H	-2.97605577	-0.63234809	-2.55324047

C	-2.96818177	-1.71514909	-0.61522947
H	-3.25279077	-0.80838409	-0.01763647
H	-3.54249477	-2.55206509	-0.13718847
C	-0.67949377	-0.78115709	-0.92349647
H	-0.91265377	0.12399691	-0.32654447
H	0.41686323	-0.98189209	-0.84129447
H	-0.88554277	-0.54289209	-1.98567547
C	-1.18747177	-2.17551509	1.03126553
H	-0.13113977	-2.47449409	1.19212453
H	-1.35628277	-1.24459309	1.61559753
H	-1.84332377	-2.94624409	1.47344853
C	-1.06740277	-3.20503209	-1.20197647
H	0.02280323	-3.43622109	-1.02322547
H	-1.64571877	-4.08772609	-0.88929047
H	-1.17469977	-3.07798409	-2.29033747
H	-6.15606977	0.13249391	3.62361353
H	1.17498823	2.22471991	-5.82876947
C	-4.43675977	1.07973691	2.60212353
H	-4.47851477	1.97360091	3.27065553
O	-5.11999377	1.30127991	1.37063053
H	-5.48298077	2.22528991	1.32702253
O	-0.40133277	3.82877791	-4.37199547
H	-1.12571577	4.19350291	-3.77363047
C	-0.73432377	2.48285191	-4.74744847
H	-1.31423377	2.53244791	-5.70323847
O	-4.45459777	-0.43007709	4.51536453

H	-3.46758777	-0.56902209	4.37270853
I	2.50261723	-2.80352309	-0.67861947
I	-3.59421777	4.57332191	1.53559953
H	-3.82745477	4.26850291	-0.07102847
I	-6.46505077	-3.92190809	-0.52672647
H	-5.72243077	-2.63074209	-1.35195647
